# Supplementary material for: Synthesis and In Vitro Antibacterial Activity of Quaternized 10-Methoxycanthin-6-one Derivatives
Source: Molecules. 2019 Apr 19;24(8):1553. doi: 10.3390/molecules24081553 (PMC6514585; doi:10.3390/molecules24081553)
Supplement: Supplementary file 1 [file molecules-24-01553-s001.pdf]

Supporting information

Synthesis and *In Vitro* Antibacterial Activity of Quaternized 10-Methoxycanthin-6-one Derivatives

Na Li [1], Dan Liu [2], Jiang-Kun Dai [2], Jin-Yi Wang [1,2,*] and Jun-Ru Wang [2,*]

[1] Colleges of Veterinary Medicine, Northwest A&F University, Yangling, Shaanxi 712100, China; lnuk@nwsuaf.edu.cn (N. L.); jywang@nwsuaf.edu.cn (J.-Y. W.);

[2] Colleges of Chemistry and Pharmacy, Northwest A&F University, Yangling, Shaanxi 712100, China; ld127222@126.com (D. L.); daijkun@hotmail.com (J.-K. D.) wangjunru@nwafu.edu.cn (J.-R. W.);

* Correspondence: wangjunru@nwafu.edu.cn (J.-R. W.); jywang@nwsuaf.edu.cn (J.-Y. W.);

Contents

The NMR, HRMS and HPLC Spectra of Compounds **5** and **6a–6v**.

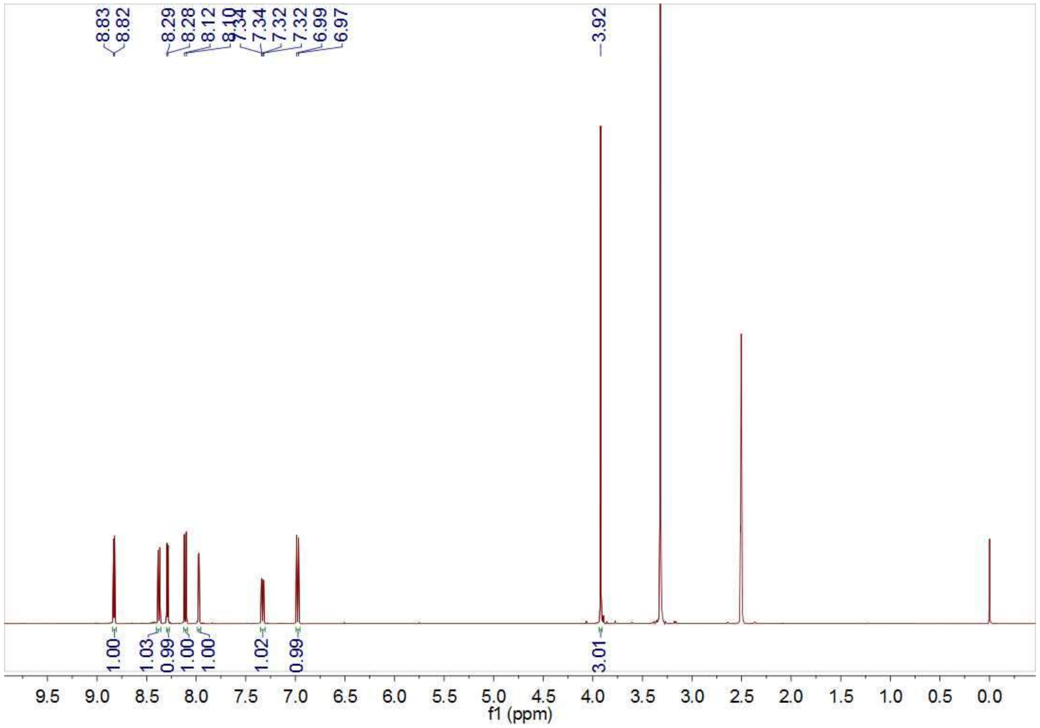

8.83
8.82
8.29
8.28
8.12
8.10
7.34
7.34
7.32
7.32
6.99
6.97
3.92
1.00
1.03
0.99
1.00
1.00
1.02
0.99
3.01
9.5
9.0
8.5
8.0
7.5
7.0
6.5
6.0
5.5
5.0
4.5
4.0
3.5
3.0
2.5
2.0
1.5
1.0
0.5
0.0
f1 (ppm)

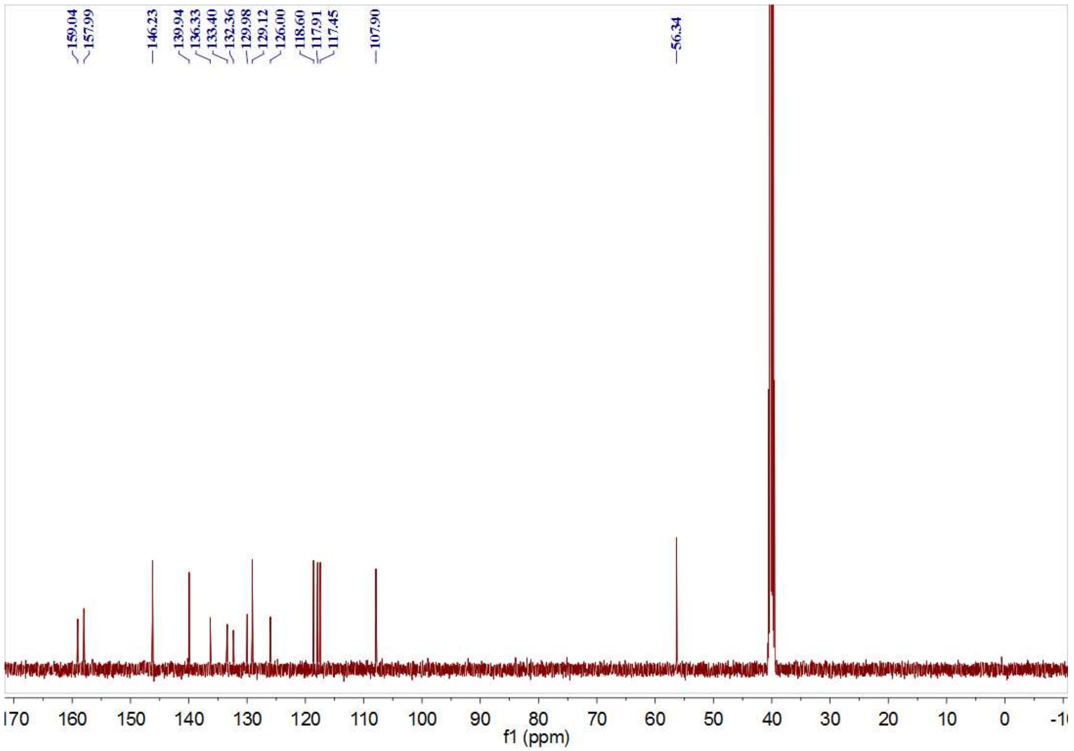
159.04
157.99
146.23
139.94
136.33
133.40
132.36
129.98
129.12
126.00
118.60
117.91
117.45
107.90
56.34
f1 (ppm)

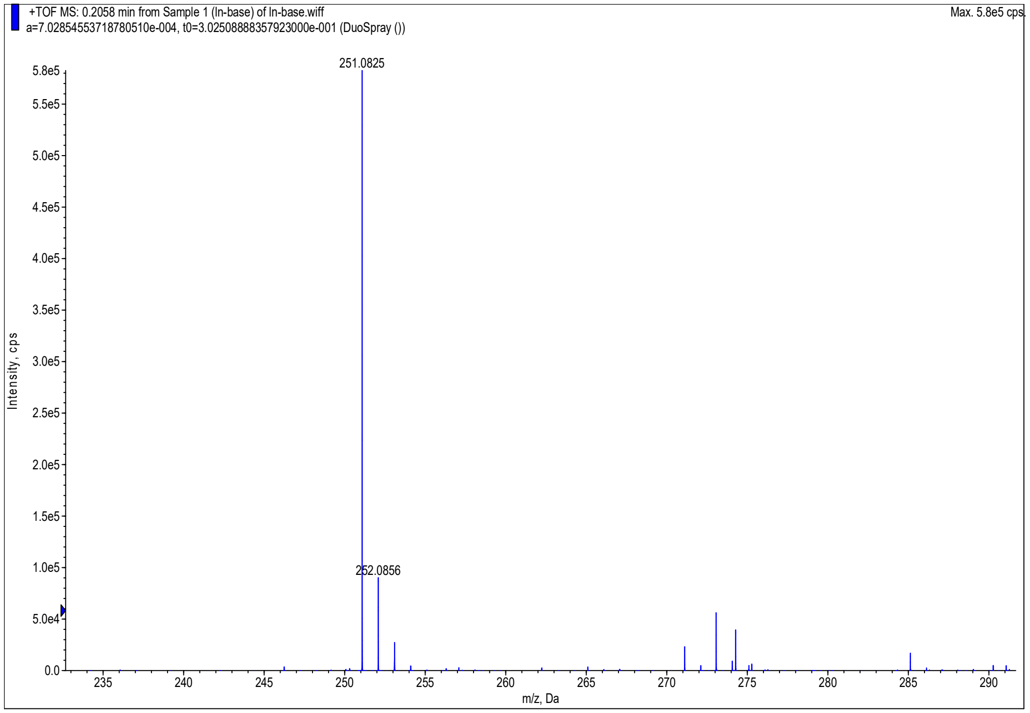
+TOF MS: 0.2058 min from Sample 1 (In-base) of In-base.wiff
a=7.02854553718780510e-004, t0=3.02508888357923000e-001 (DuoSpray ())
Max. 5.8e5 cps.
251.0825
252.0856
Intensity, cps
m/z, Da

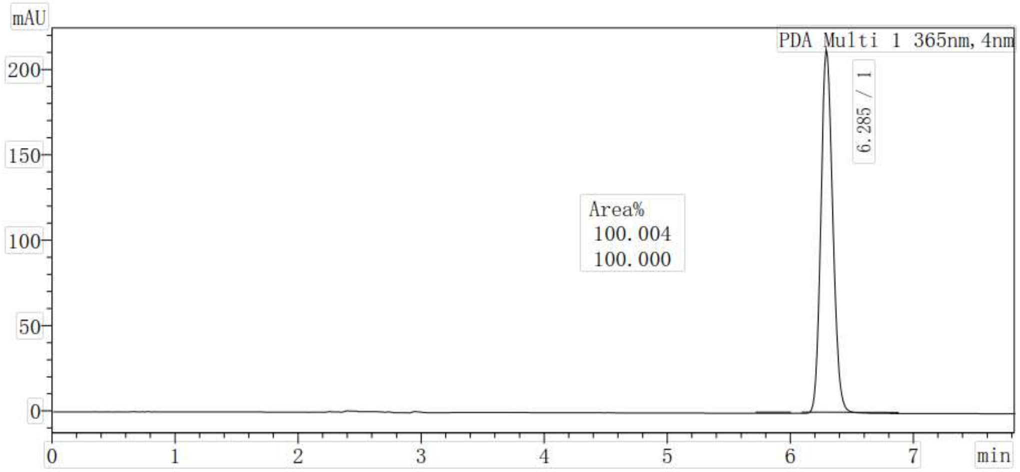

Figure S1. The NMR, HRMS and HPLC spectra of compound **5**.

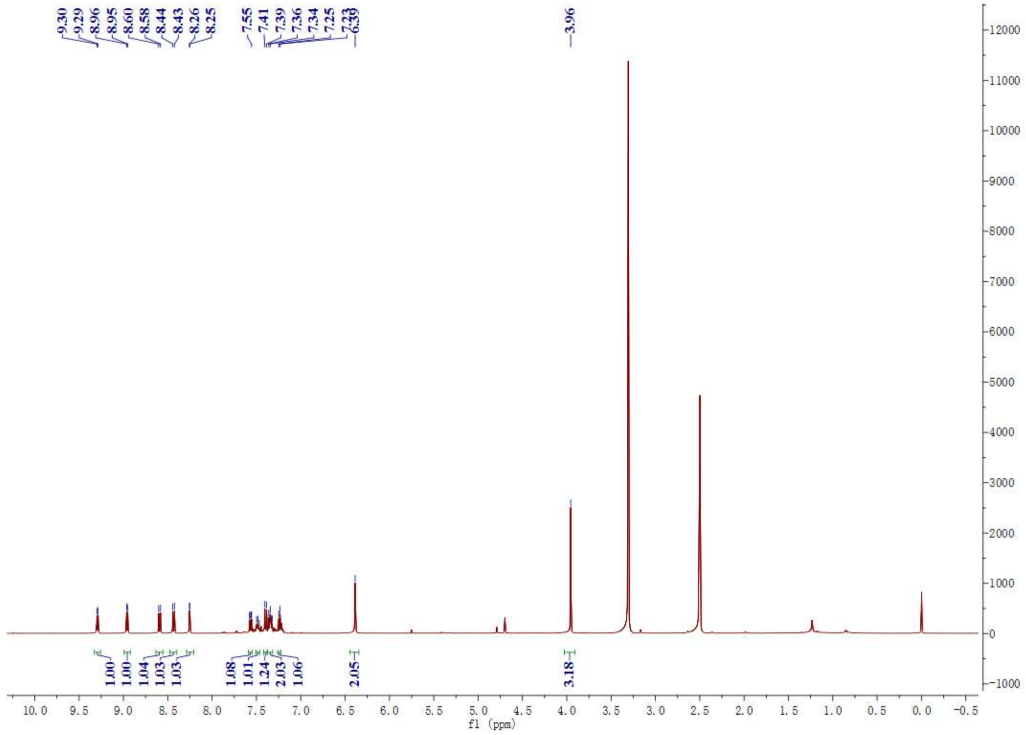

9.30
9.29
8.96
8.95
8.60
8.58
8.44
8.43
8.26
8.25
7.55
7.41
7.39
7.36
7.34
7.25
7.23
6.39
3.96
1.00
1.00
1.04
1.03
1.03
1.08
1.01
1.24
2.03
1.06
2.05
3.18
f1 (ppm)

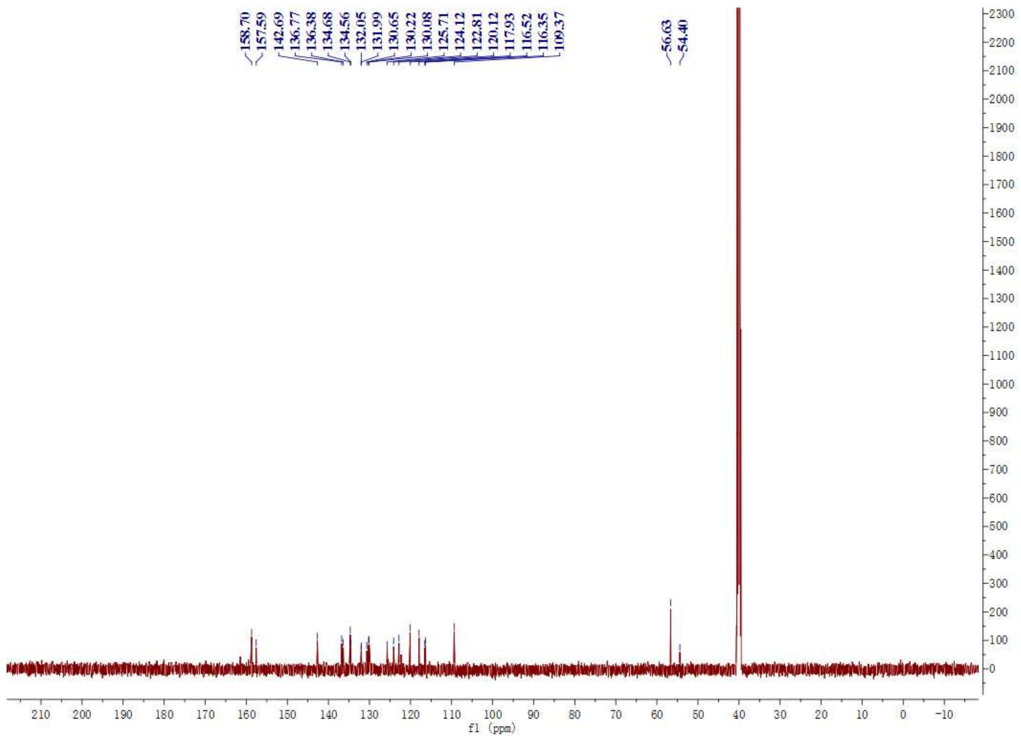

158.70
157.59
142.69
136.77
136.38
134.68
134.56
132.05
131.99
130.65
130.22
130.08
125.71
124.12
122.81
120.12
117.93
116.52
116.35
109.37
56.63
54.40
f1 (ppm)

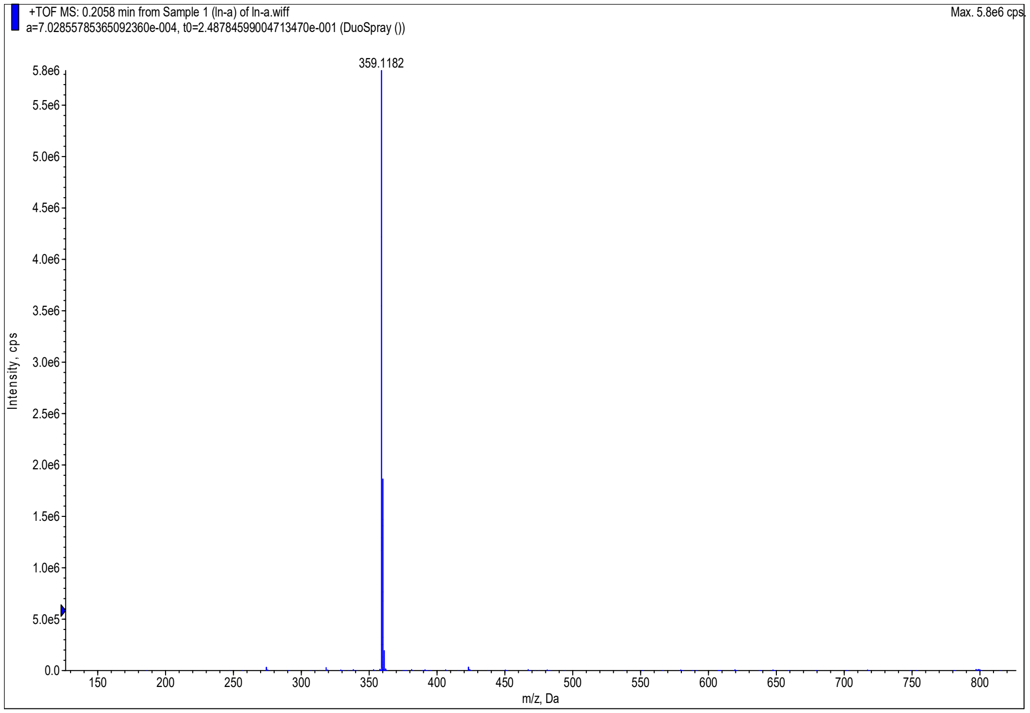
+TOF MS: 0.2058 min from Sample 1 (In-a) of In-a.wiff
a=7.02855785365092360e-004, t0=2.48784599004713470e-001 (DuoSpray ())
Max. 5.8e6 cps.
359.1182
Intensity, cps
m/z, Da
5.8e6
5.5e6
5.0e6
4.5e6
4.0e6
3.5e6
3.0e6
2.5e6
2.0e6
1.5e6
1.0e6
5.0e5
0.0
150
200
250
300
350
400
450
500
550
600
650
700
750
800

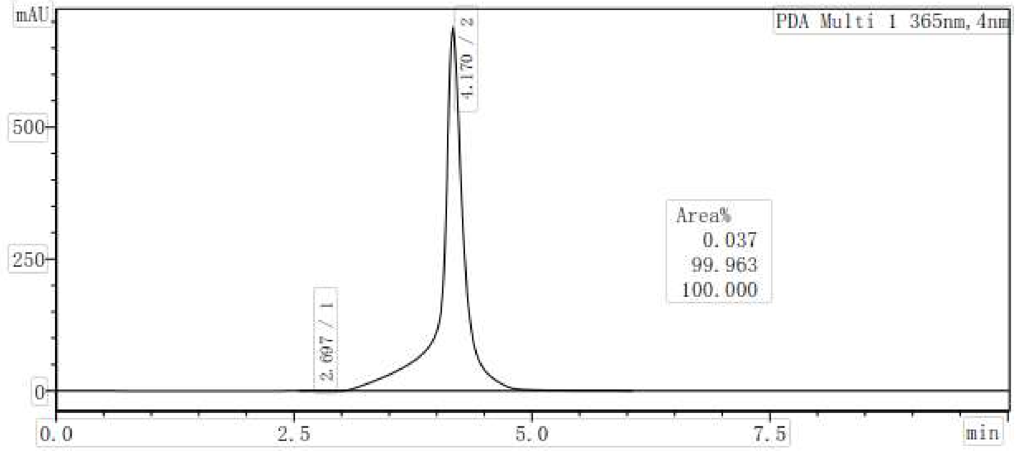

Figure S2. The NMR, HRMS and HPLC spectra of compound **6a**.

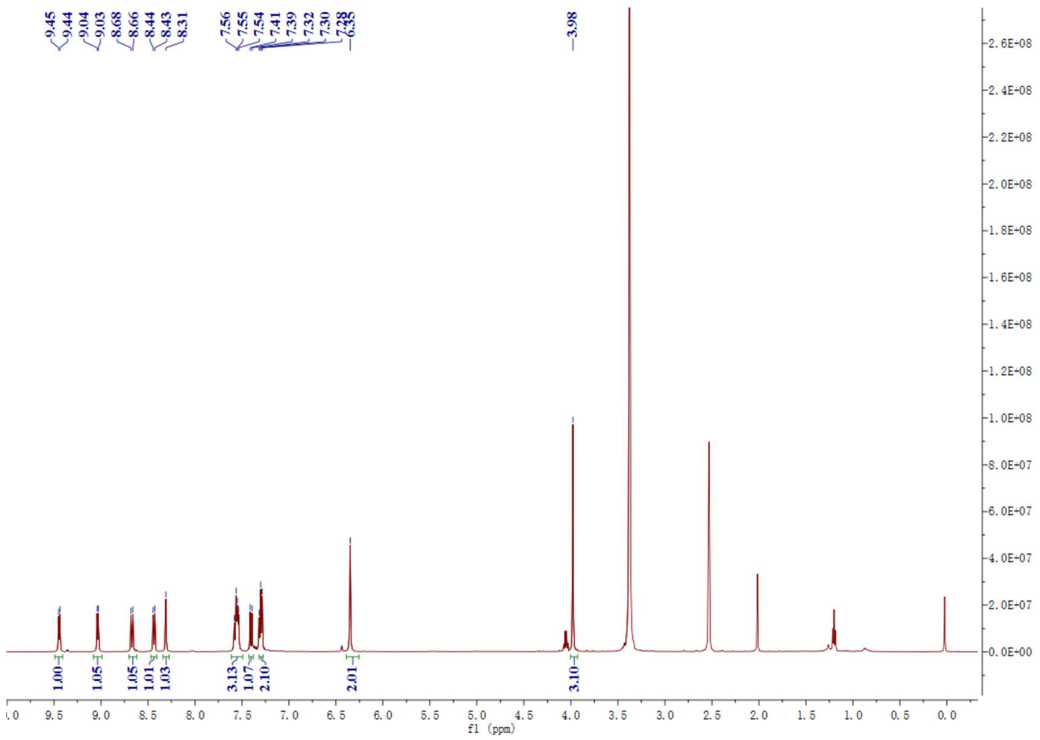
9.45
9.44
9.04
9.03
8.68
8.66
8.44
8.43
8.31
7.56
7.55
7.54
7.41
7.39
7.32
7.30
7.28
6.35
3.98
1.00
1.05
1.05
1.01
1.03
3.13
1.07
2.10
2.01
3.10
f1 (ppm)

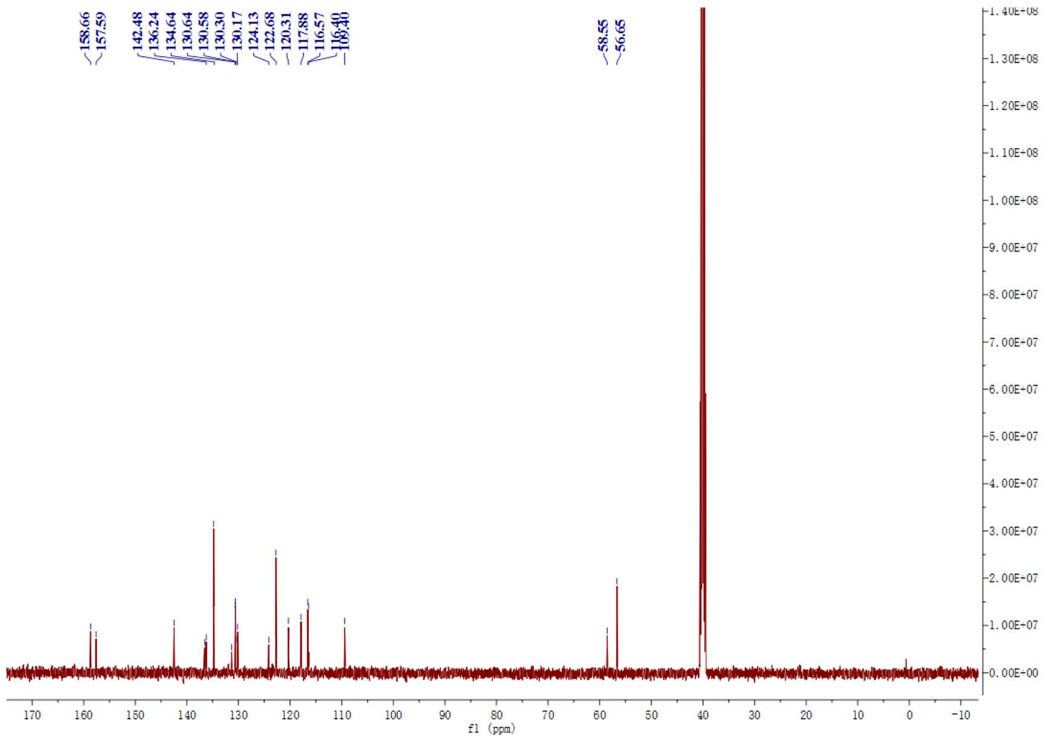

158.66
157.59
142.48
136.24
134.64
130.64
130.58
130.30
130.17
124.13
122.68
120.31
117.88
116.57
116.40
109.40
58.55
56.65
1.40E+08
1.30E+08
1.20E+08
1.10E+08
1.00E+08
9.00E+07
8.00E+07
7.00E+07
6.00E+07
5.00E+07
4.00E+07
3.00E+07
2.00E+07
1.00E+07
0.00E+00
170 160 150 140 130 120 110 100 90 80 70 60 50 40 30 20 10 0 -10
f1 (ppm)

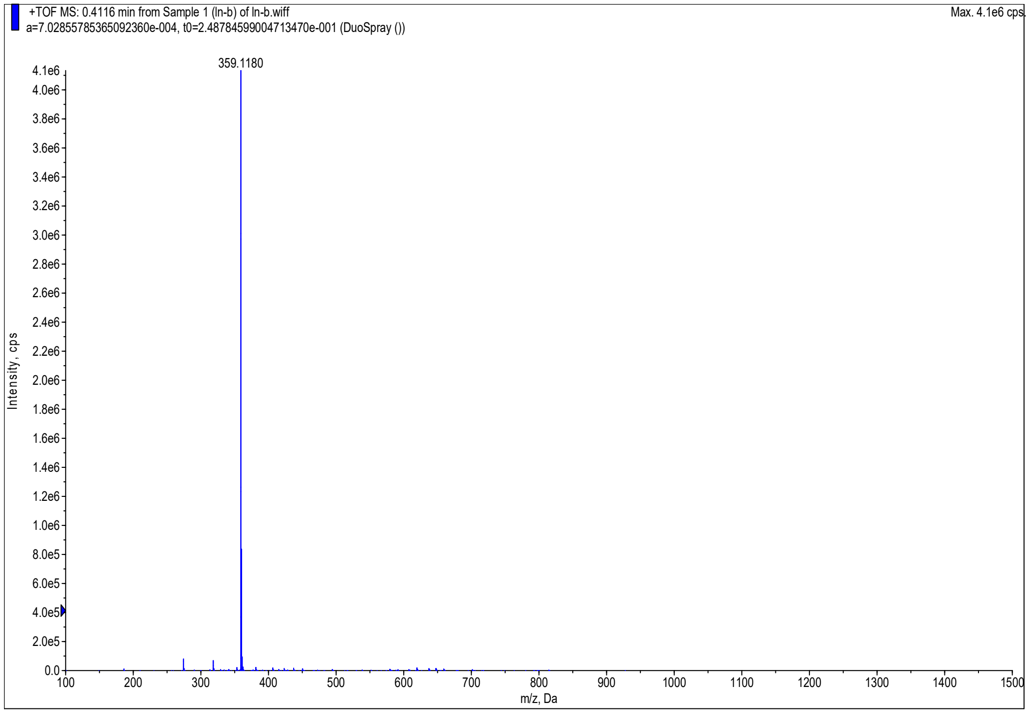
+TOF MS: 0.4116 min from Sample 1 (ln-b) of ln-b.wiff
a=7.02855785365092360e-004, t0=2.48784599004713470e-001 (DuoSpray ())
Max. 4.1e6 cps.
359.1180
Intensity, cps
m/z, Da

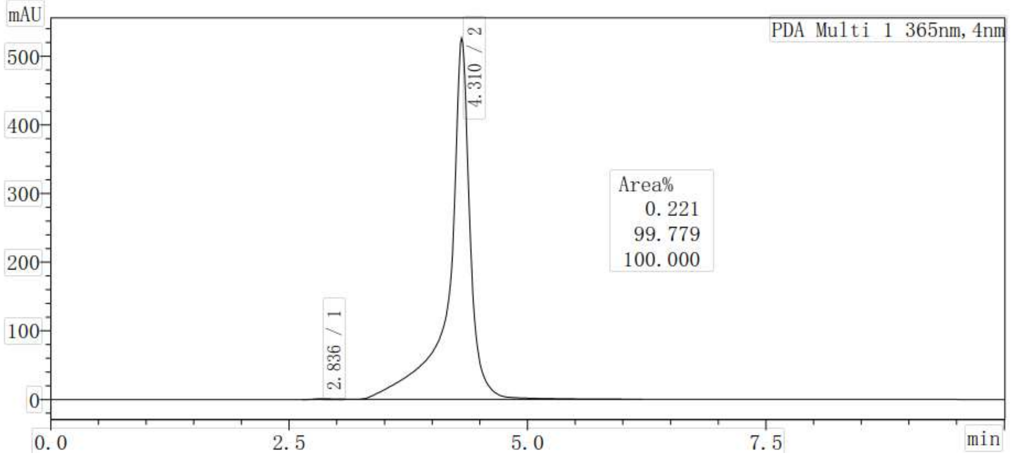

Figure S3. The NMR, HRMS and HPLC spectra of compound **6b**.

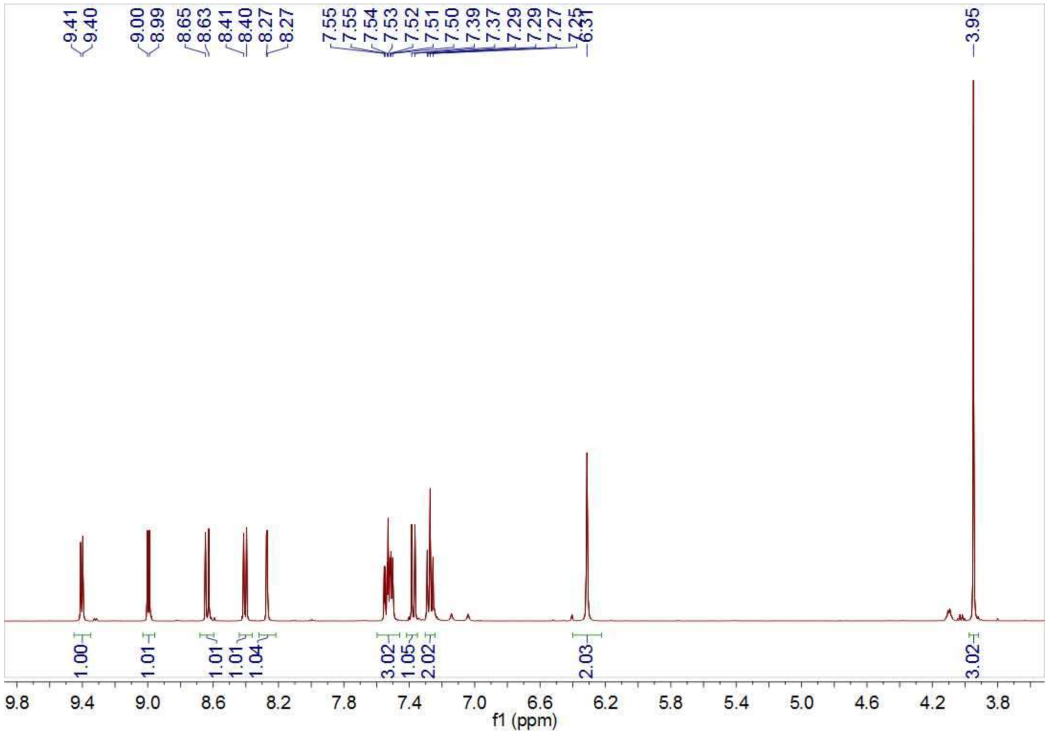

9.41
9.40
9.00
8.99
8.65
8.63
8.41
8.40
8.27
8.27
7.55
7.55
7.54
7.53
7.52
7.51
7.50
7.39
7.37
7.29
7.29
7.27
7.25
6.31
3.95
1.00
1.01
1.01
1.01
1.04
3.02
1.05
2.02
2.03
3.02
9.8 9.4 9.0 8.6 8.2 7.8 7.4 7.0 6.6 6.2 5.8 5.4 5.0 4.6 4.2 3.8
f1 (ppm)

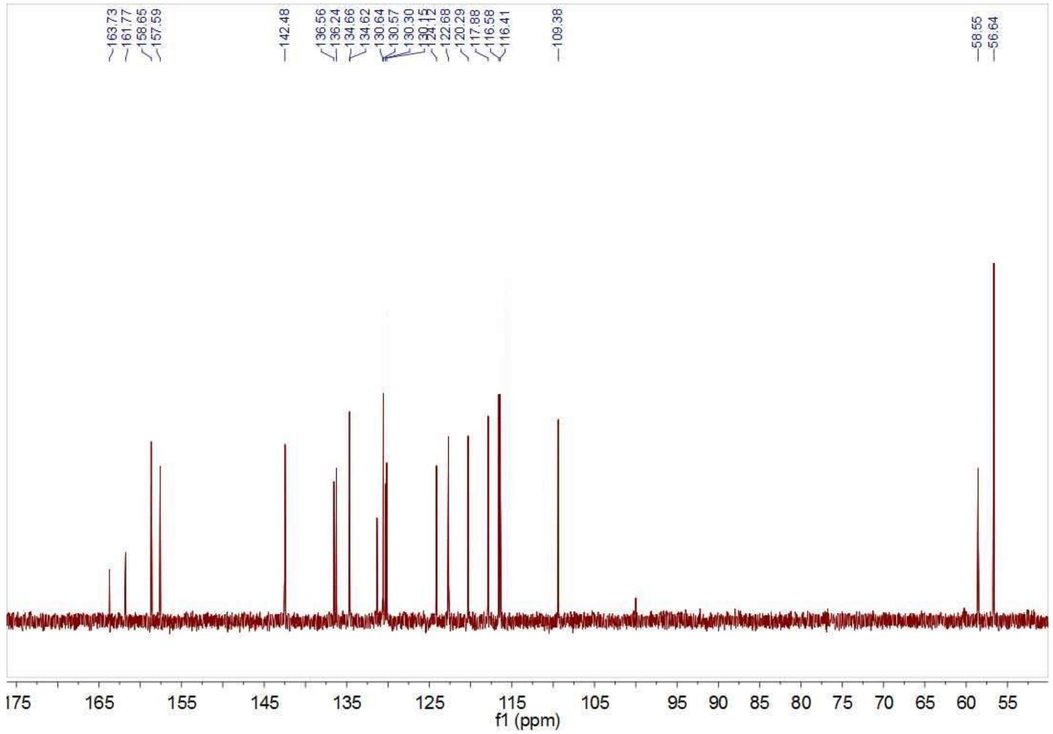

163.73
161.77
158.65
157.59
142.48
136.56
136.24
134.66
134.62
130.64
130.57
130.30
130.15
124.12
122.68
120.29
117.88
116.58
116.41
109.38
58.55
56.64
175
165
155
145
135
125
115
105
95
90
85
80
75
70
65
60
55
f1 (ppm)

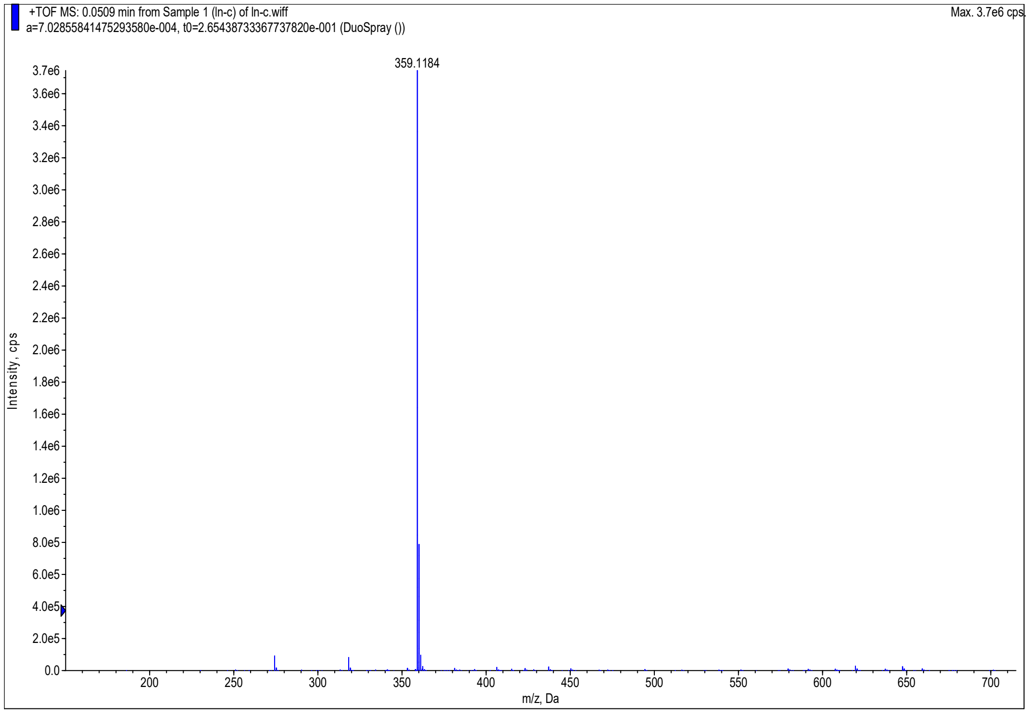

+TOF MS: 0.0509 min from Sample 1 (ln-c) of ln-c.wiff
a=7.02855841475293580e-004, t0=2.65438733367737820e-001 (DuoSpray ())
Max. 3.7e6 cps.
359.1184
Intensity, cps
m/z, Da

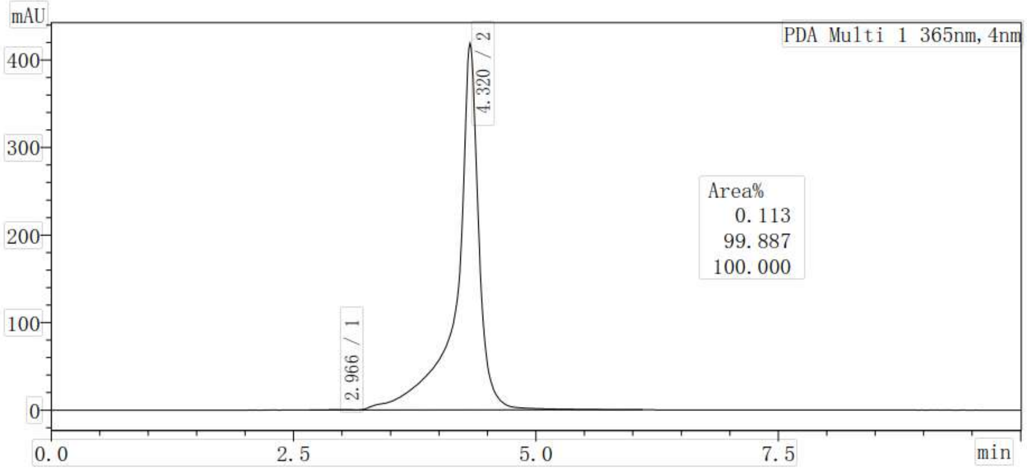

Figure S4. The NMR, HRMS and HPLC spectra of compound **6c**.

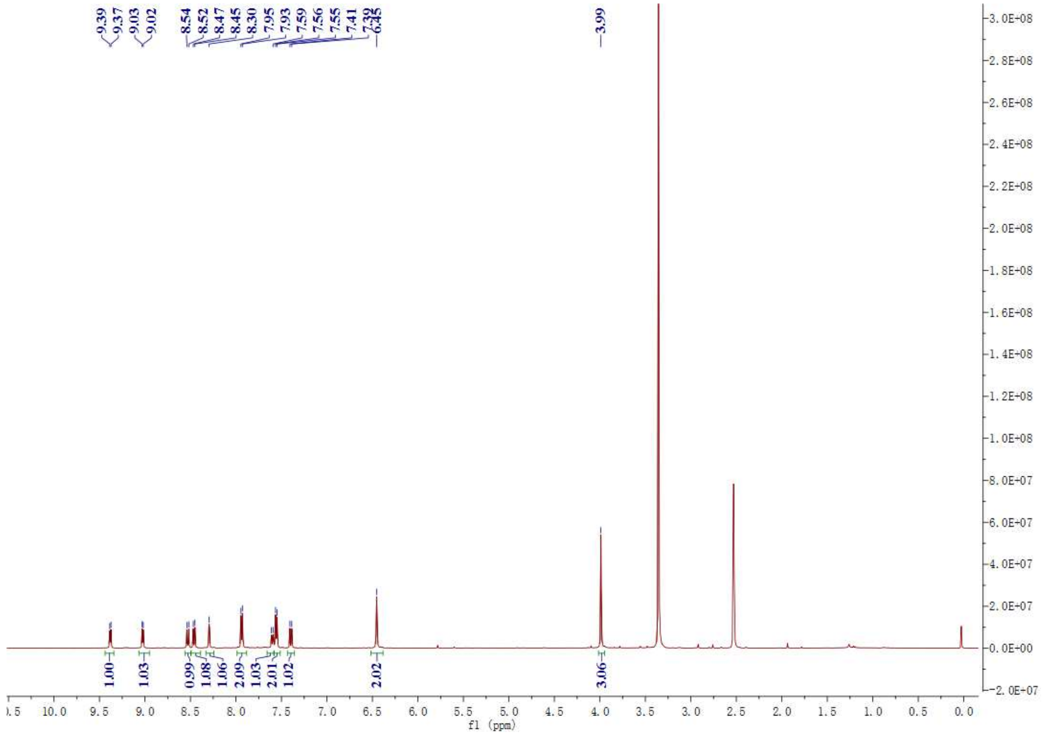

9.39
9.37
9.03
9.02
8.54
8.52
8.47
8.45
8.30
7.95
7.93
7.59
7.56
7.55
7.41
7.39
6.45
3.99
1.00
1.03
0.99
1.08
1.06
2.09
1.03
2.01
1.02
2.02
3.06
f1 (ppm)

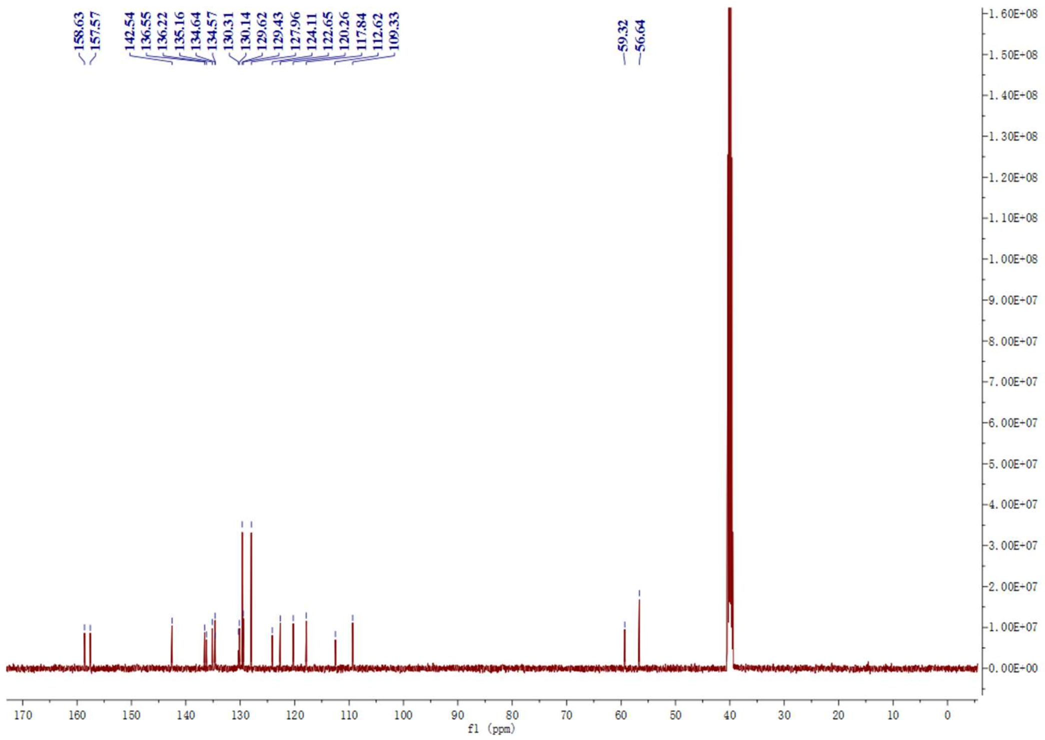

158.63
157.57
142.54
136.55
136.22
135.16
134.64
134.57
130.31
130.14
129.62
129.43
127.96
124.11
122.65
120.26
117.84
112.62
109.33
59.32
56.64
1.60E+08
1.50E+08
1.40E+08
1.30E+08
1.20E+08
1.10E+08
1.00E+08
9.00E+07
8.00E+07
7.00E+07
6.00E+07
5.00E+07
4.00E+07
3.00E+07
2.00E+07
1.00E+07
0.00E+00
170 160 150 140 130 120 110 100 90 80 70 60 50 40 30 20 10 0
f1 (ppm)

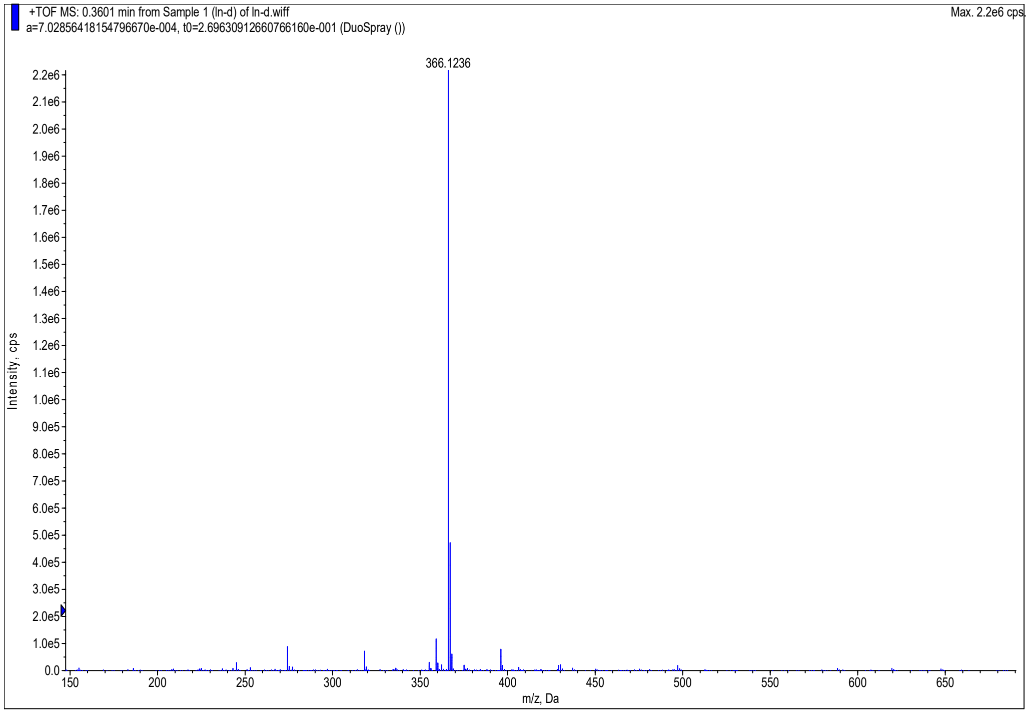

+TOF MS: 0.3601 min from Sample 1 (ln-d) of ln-d.wiff
a=7.02856418154796670e-004, t0=2.69630912660766160e-001 (DuoSpray ())
Max. 2.2e6 cps.
366.1236
Intensity, cps
m/z, Da

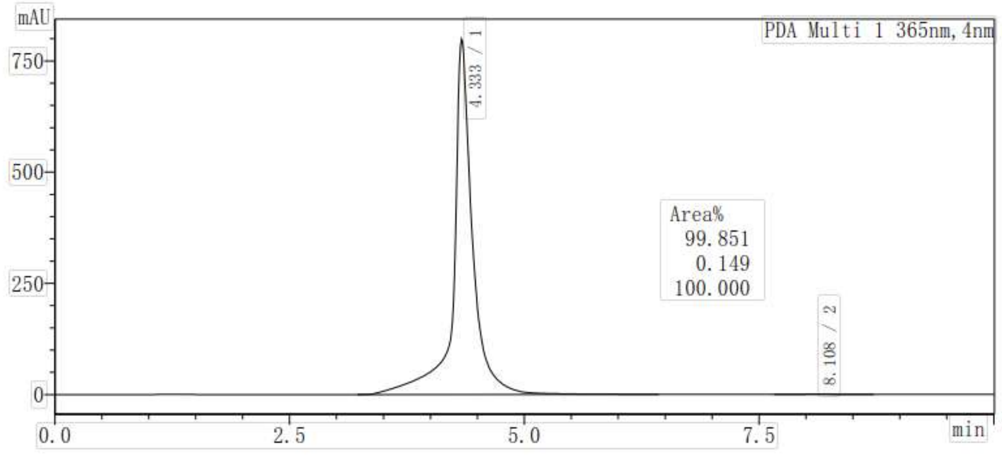

Figure S5. The NMR, HRMS and HPLC spectra of compound **6d**.

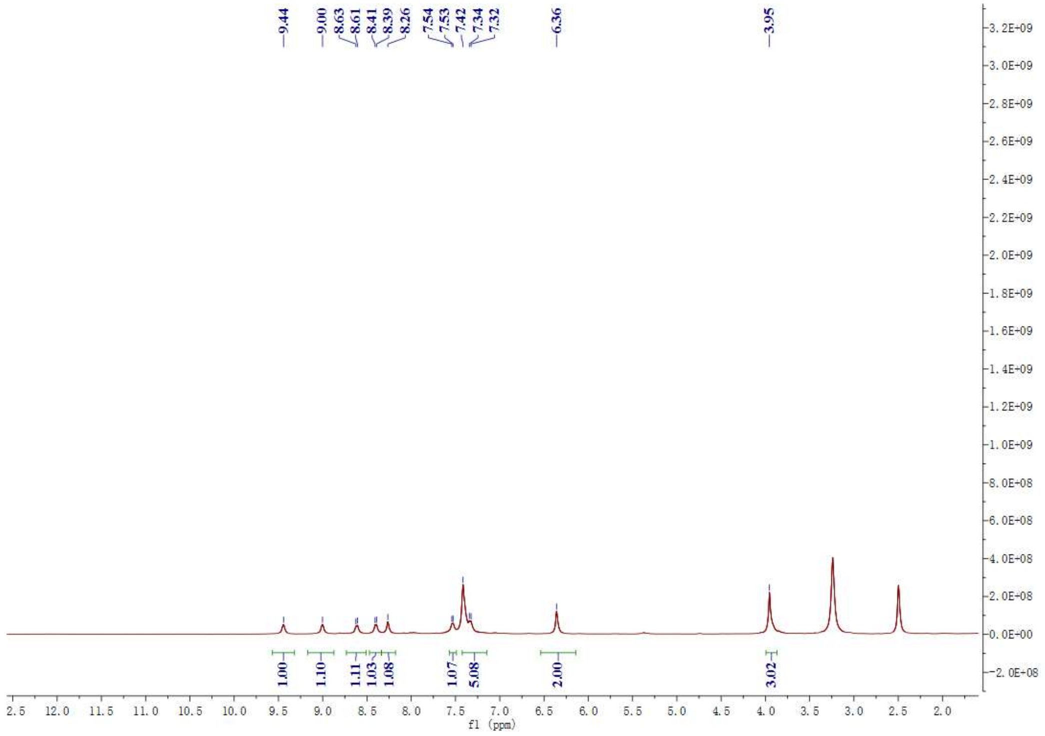
9.44
9.00
8.63
8.61
8.41
8.39
8.26
7.54
7.53
7.42
7.34
7.32
6.36
3.95
1.00
1.10
1.11
1.03
1.08
1.07
5.08
2.00
3.02
f1 (ppm)

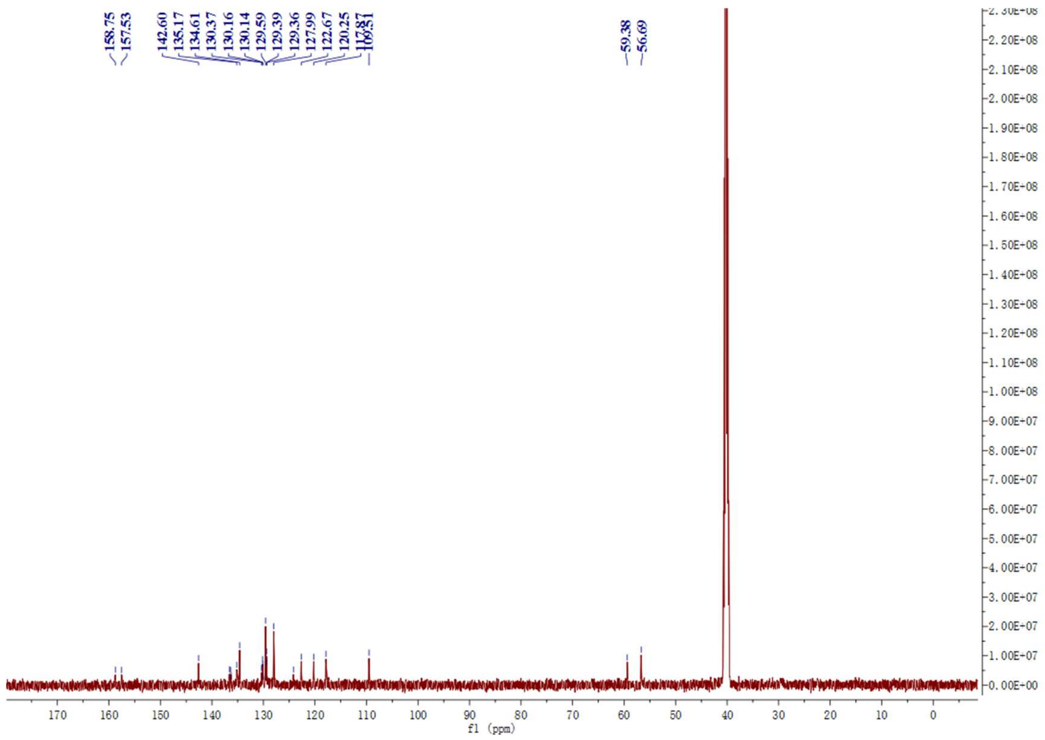

158.75
157.53
142.60
135.17
134.61
130.37
130.16
130.14
129.59
129.39
129.36
127.99
122.67
120.25
117.87
109.51
59.38
56.69
2.30E+08
2.20E+08
2.10E+08
2.00E+08
1.90E+08
1.80E+08
1.70E+08
1.60E+08
1.50E+08
1.40E+08
1.30E+08
1.20E+08
1.10E+08
1.00E+08
9.00E+07
8.00E+07
7.00E+07
6.00E+07
5.00E+07
4.00E+07
3.00E+07
2.00E+07
1.00E+07
0.00E+00
170
160
150
140
130
120
110
100
90
80
70
60
50
40
30
20
10
0
f1 (ppm)

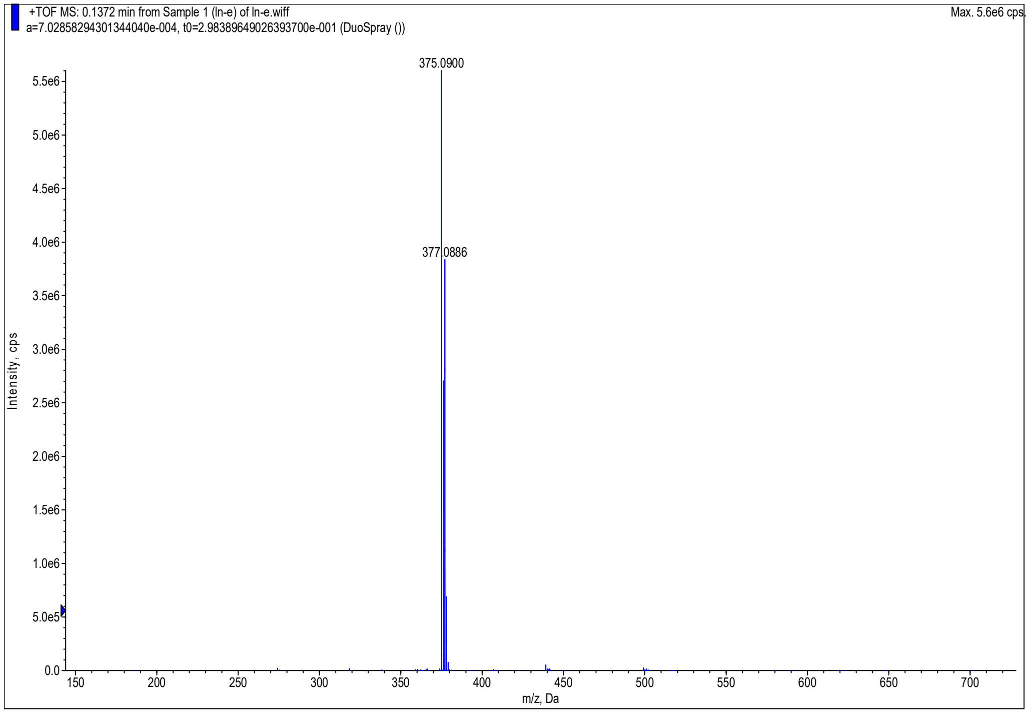
+TOF MS: 0.1372 min from Sample 1 (In-e) of In-e.wiff
a=7.02858294301344040e-004, t0=2.98389649026393700e-001 (DuoSpray ())
Max. 5.6e6 cps.
375.0900
377.0886
Intensity, cps
m/z, Da

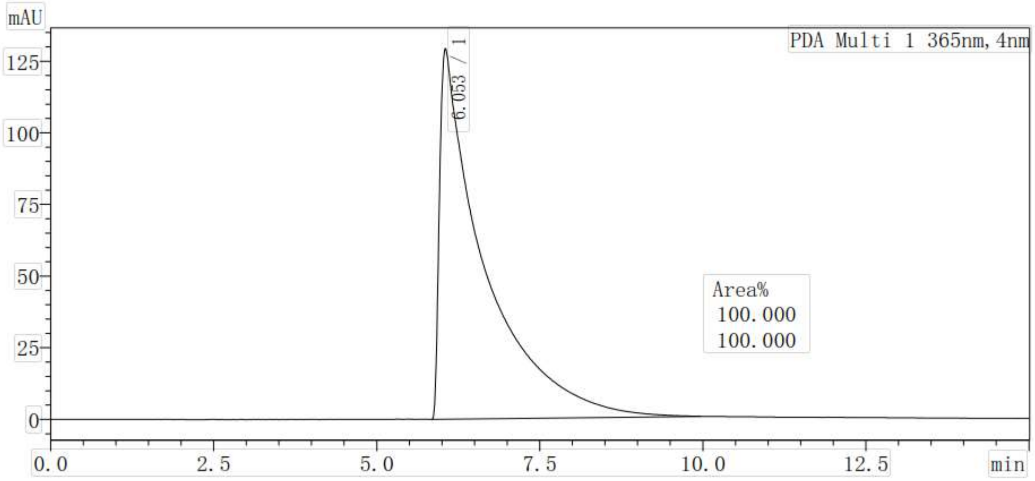

Figure S6. The NMR, HRMS and HPLC spectra of compound **6e**.

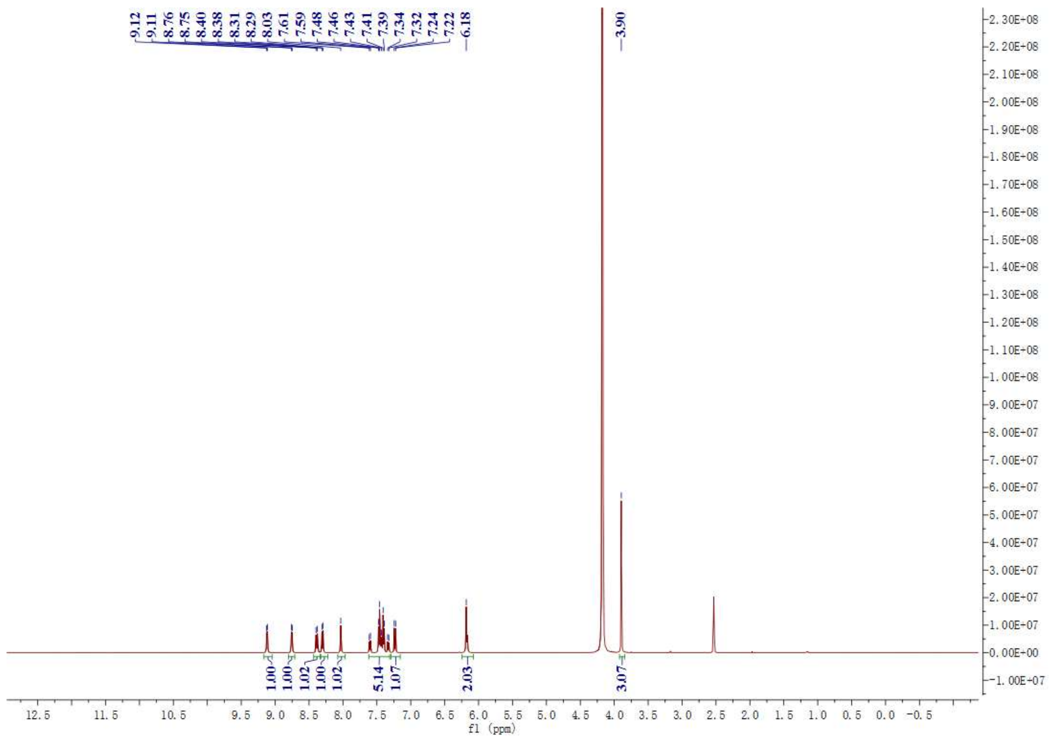

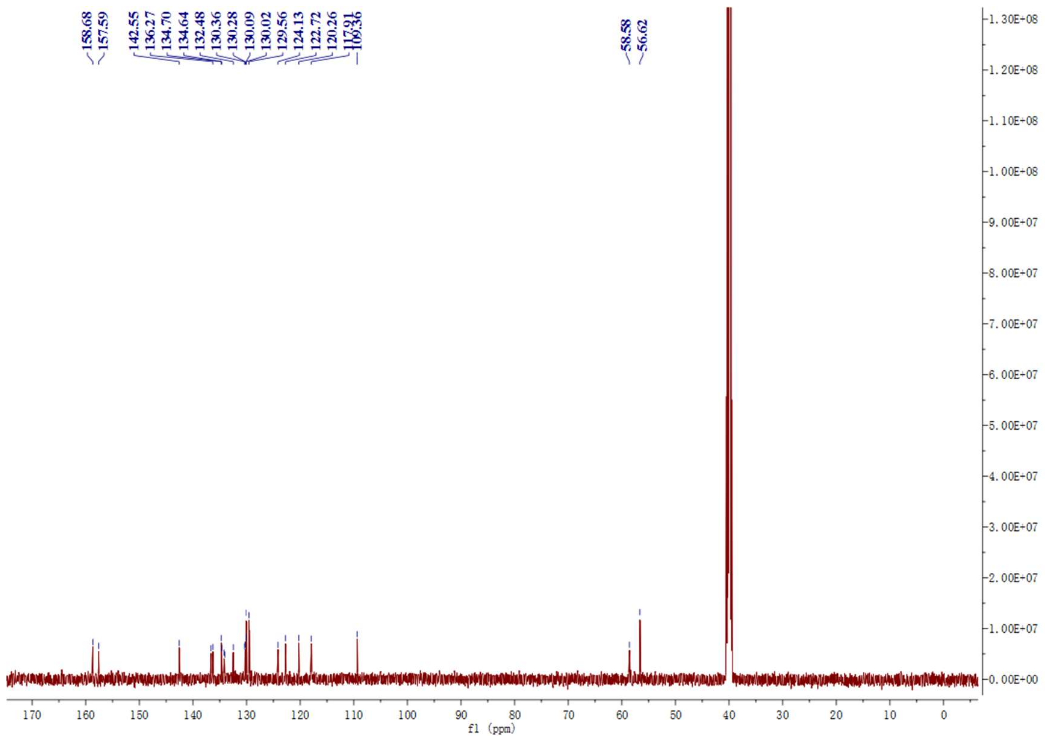
158.68
157.59
142.55
136.27
134.70
134.64
132.48
130.36
130.28
130.09
130.02
129.56
124.13
122.72
120.26
109.36
58.58
56.62
f1 (ppm)

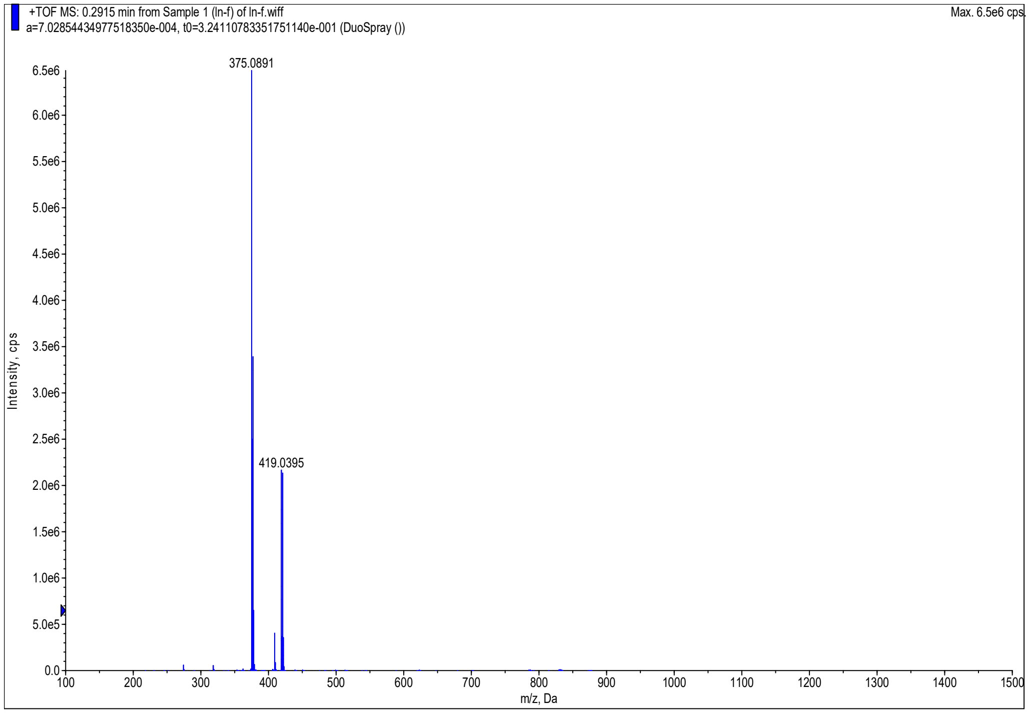
+TOF MS: 0.2915 min from Sample 1 (ln-f) of ln-f.wiff
a=7.02854434977518350e-004, t0=3.24110783351751140e-001 (DuoSpray ())
Max. 6.5e6 cps.
375.0891
419.0395
Intensity, cps
m/z, Da
6.5e6
6.0e6
5.5e6
5.0e6
4.5e6
4.0e6
3.5e6
3.0e6
2.5e6
2.0e6
1.5e6
1.0e6
5.0e5
0.0
100
200
300
400
500
600
700
800
900
1000
1100
1200
1300
1400
1500

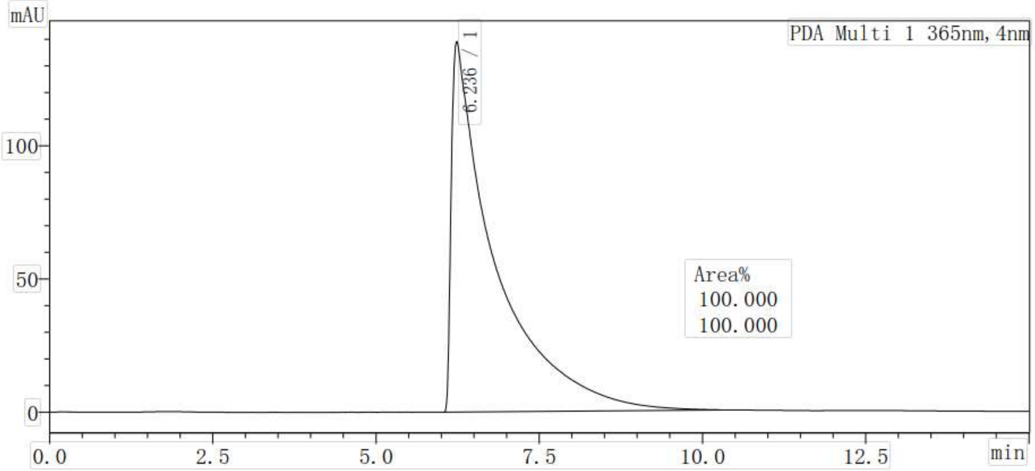

Figure S7. The NMR, HRMS and HPLC spectra of compound **6f**.

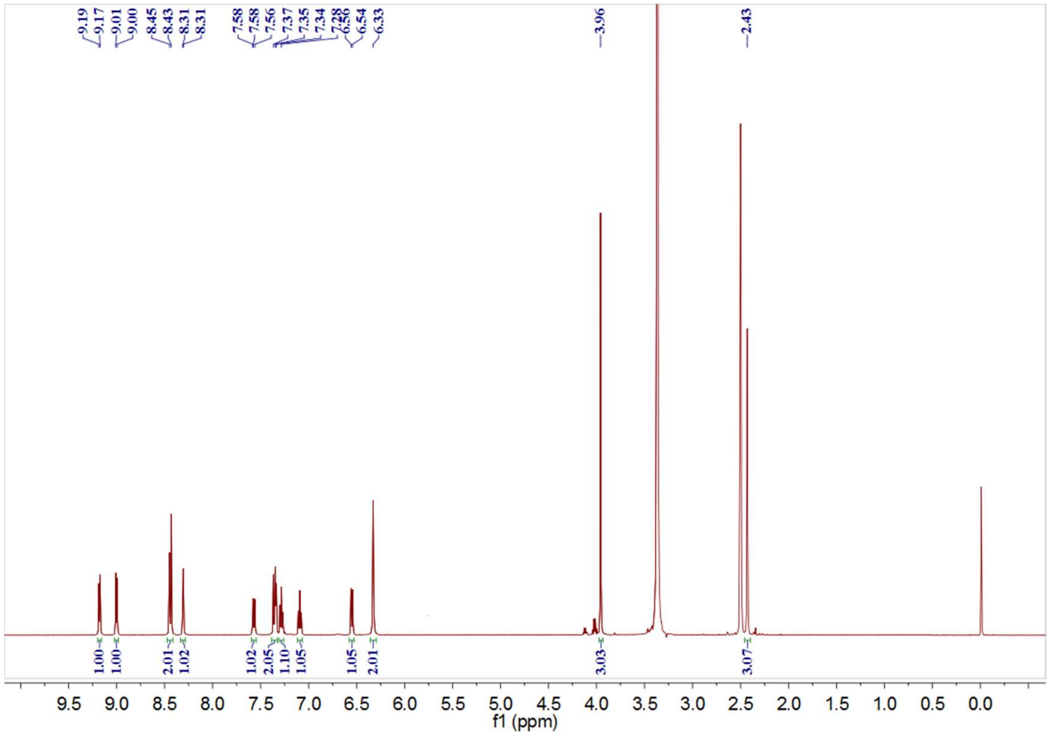

9.19
9.17
9.01
9.00
8.45
8.43
8.31
8.31
7.58
7.58
7.56
7.37
7.35
7.34
7.28
6.56
6.54
6.33
3.96
2.43
1.00
1.00
2.01
1.02
1.02
2.05
1.10
1.05
1.05
2.01
3.03
3.07
9.5 9.0 8.5 8.0 7.5 7.0 6.5 6.0 5.5 5.0 4.5 4.0 3.5 3.0 2.5 2.0 1.5 1.0 0.5 0.0
f1 (ppm)

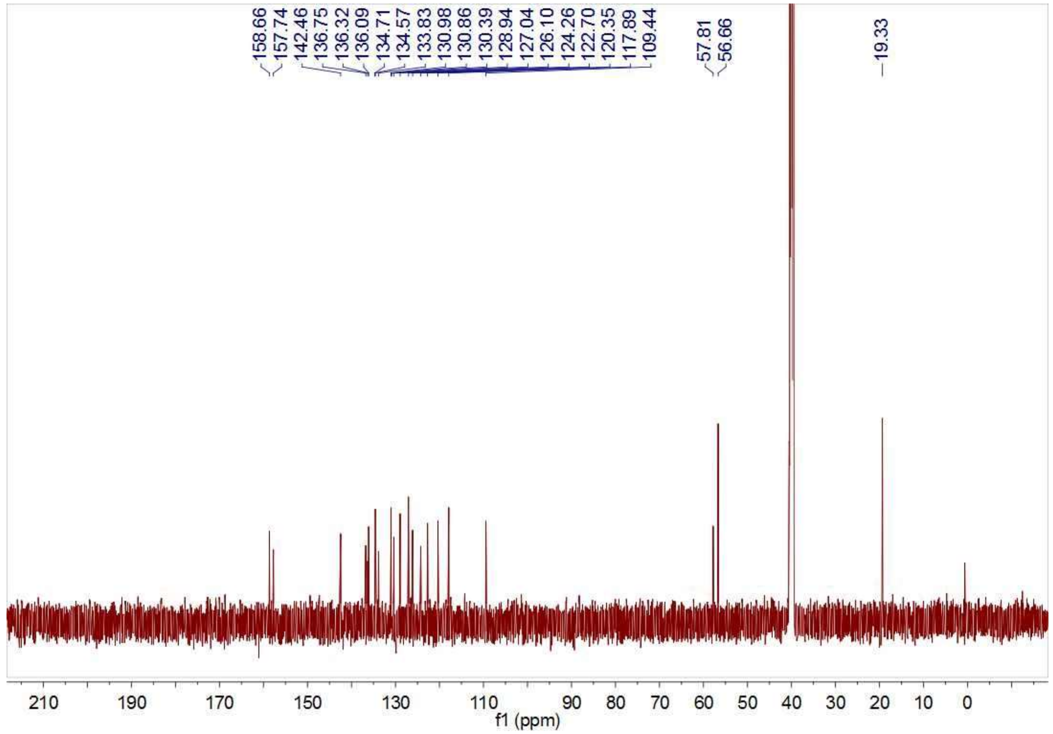

158.66
157.74
142.46
136.75
136.32
136.09
134.71
134.57
133.83
130.98
130.86
130.39
128.94
127.04
126.10
124.26
122.70
120.35
117.89
109.44
57.81
56.66
19.33
210
190
170
150
130
110
90
80
70
60
50
40
30
20
10
0
f1 (ppm)

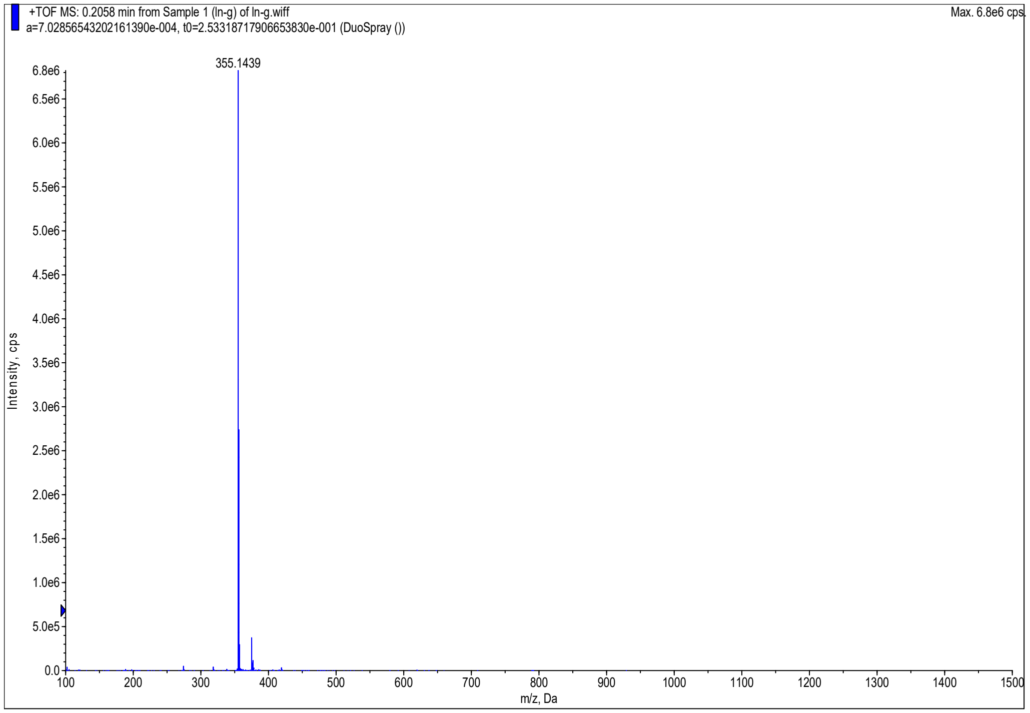
+TOF MS: 0.2058 min from Sample 1 (In-g) of In-g.wiff
a=7.02856543202161390e-004, t0=2.53318717906653830e-001 (DuoSpray ())
Max. 6.8e6 cps.
355.1439
Intensity, cps
m/z, Da

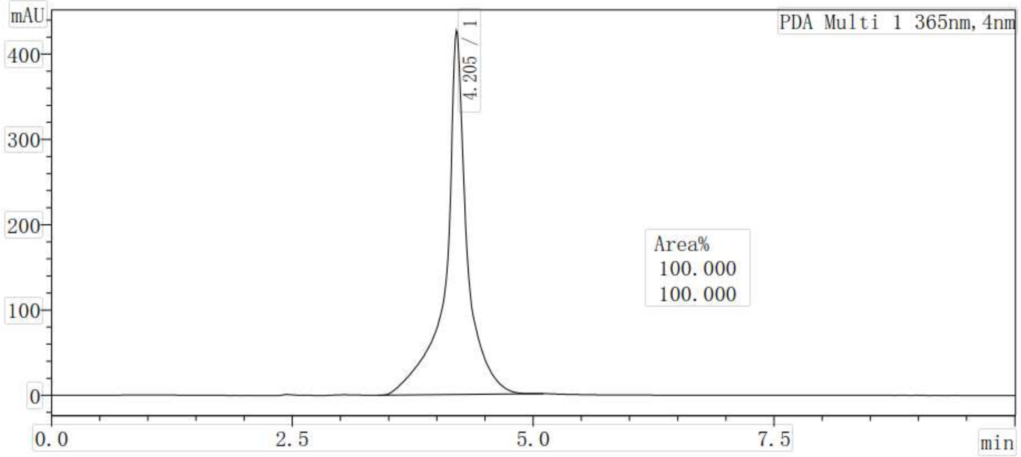

Figure S8. The NMR, HRMS and HPLC spectra of compound **6g**.

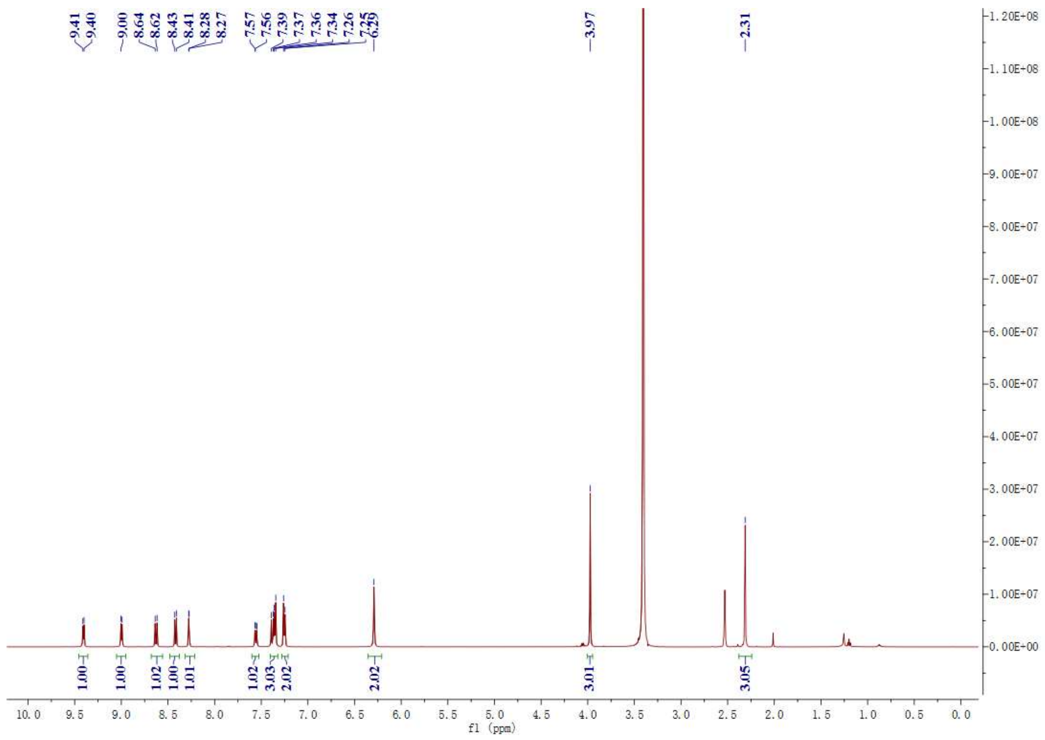

9.41
9.40
9.00
8.64
8.62
8.43
8.41
8.28
8.27
7.57
7.56
7.39
7.37
7.36
7.34
7.26
7.25
6.29
3.97
2.31
1.00
1.00
1.02
1.00
1.01
1.02
3.03
2.02
2.02
3.01
3.05
f1 (ppm)

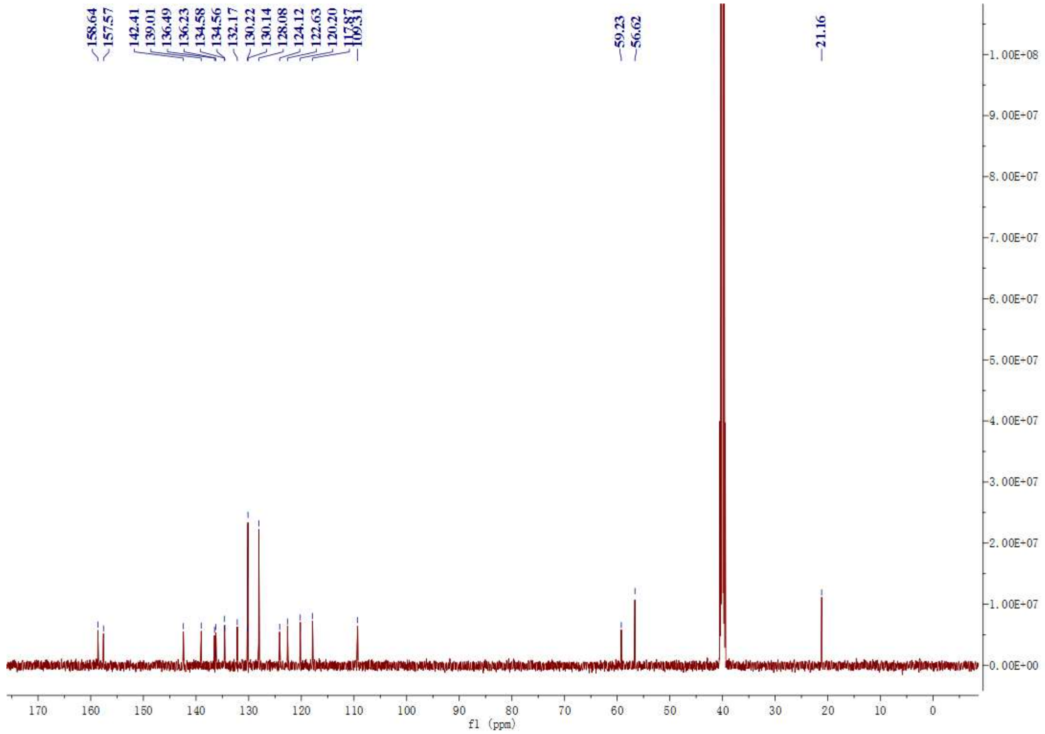

158.64
157.57
142.41
139.01
136.49
136.23
134.58
134.56
132.17
130.22
130.14
128.08
124.12
122.63
120.20
117.87
109.31
59.23
56.62
21.16
1.00E+08
9.00E+07
8.00E+07
7.00E+07
6.00E+07
5.00E+07
4.00E+07
3.00E+07
2.00E+07
1.00E+07
0.00E+00
170
160
150
140
130
120
110
100
90
80
70
60
50
40
30
20
10
0
f1 (ppm)

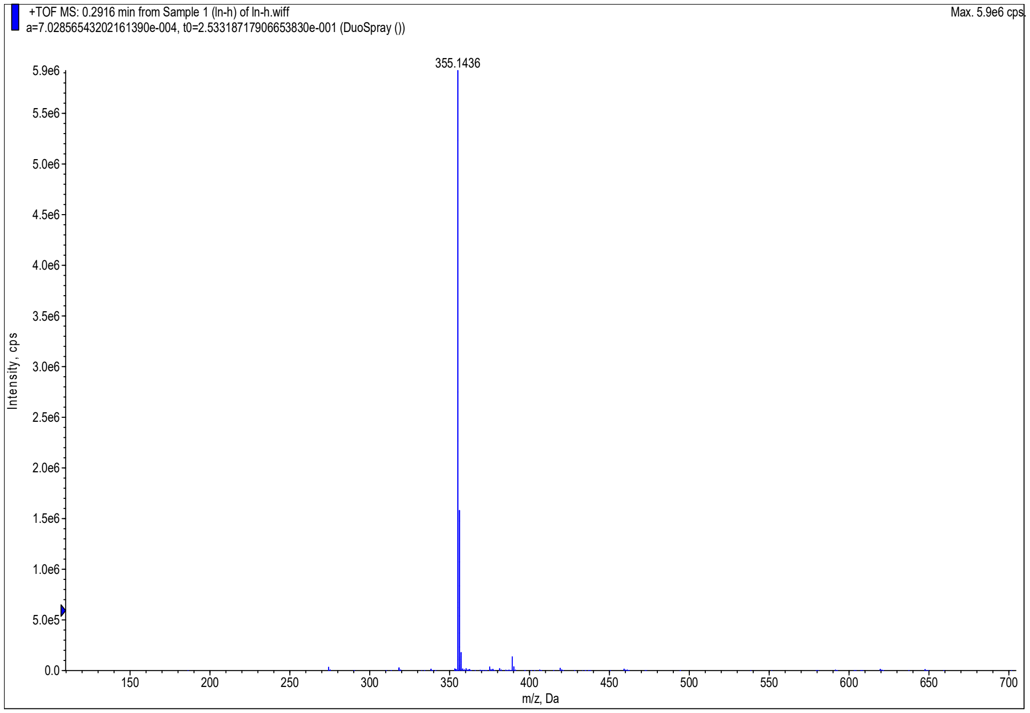
+TOF MS: 0.2916 min from Sample 1 (ln-h) of ln-h.wiff
a=7.02856543202161390e-004, t0=2.53318717906653830e-001 (DuoSpray ())
Max. 5.9e6 cps.
355.1436
Intensity, cps
5.9e6
5.5e6
5.0e6
4.5e6
4.0e6
3.5e6
3.0e6
2.5e6
2.0e6
1.5e6
1.0e6
5.0e5
0.0
150
200
250
300
350
400
450
500
550
600
650
700
m/z, Da

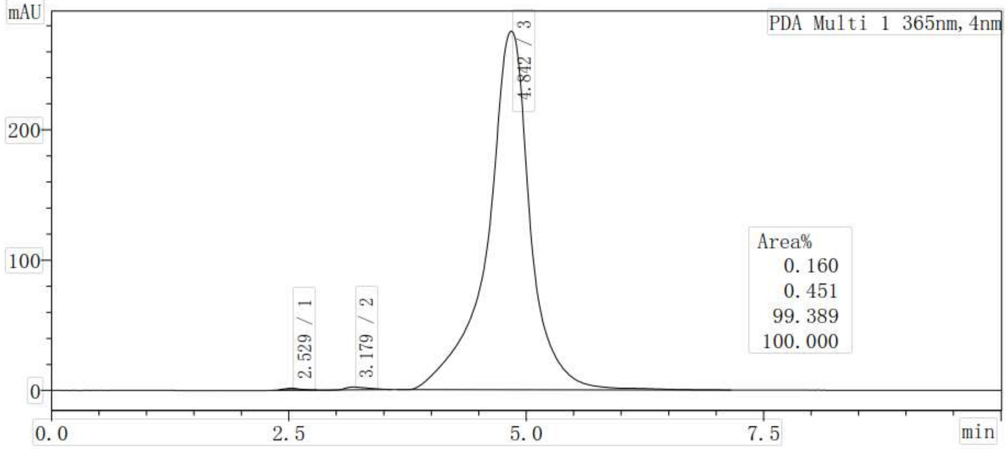

Figure S9. The NMR, HRMS and HPLC spectra of compound **6h**.

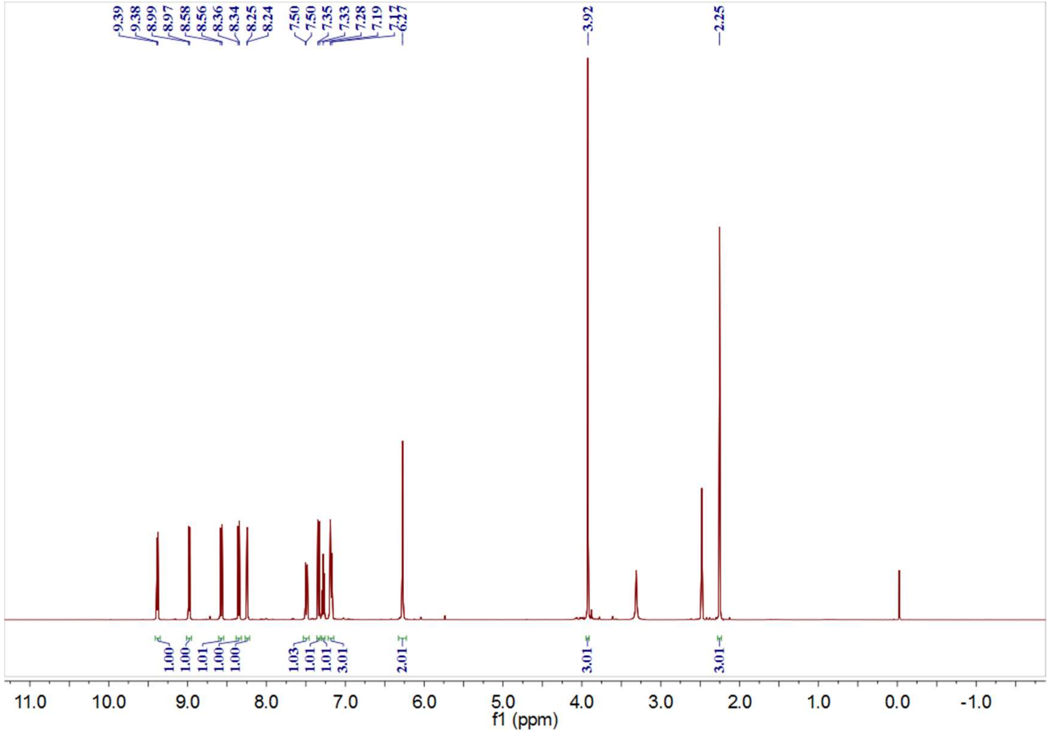
9.39
9.38
8.99
8.97
8.58
8.56
8.36
8.34
8.25
8.24
7.50
7.50
7.35
7.33
7.28
7.19
7.17
6.27
3.92
2.25
1.00
1.00
1.01
1.00
1.00
1.03
1.01
1.01
3.01
2.01
3.01
3.01
11.0
10.0
9.0
8.0
7.0
6.0
5.0
4.0
3.0
2.0
1.0
0.0
-1.0
f1 (ppm)

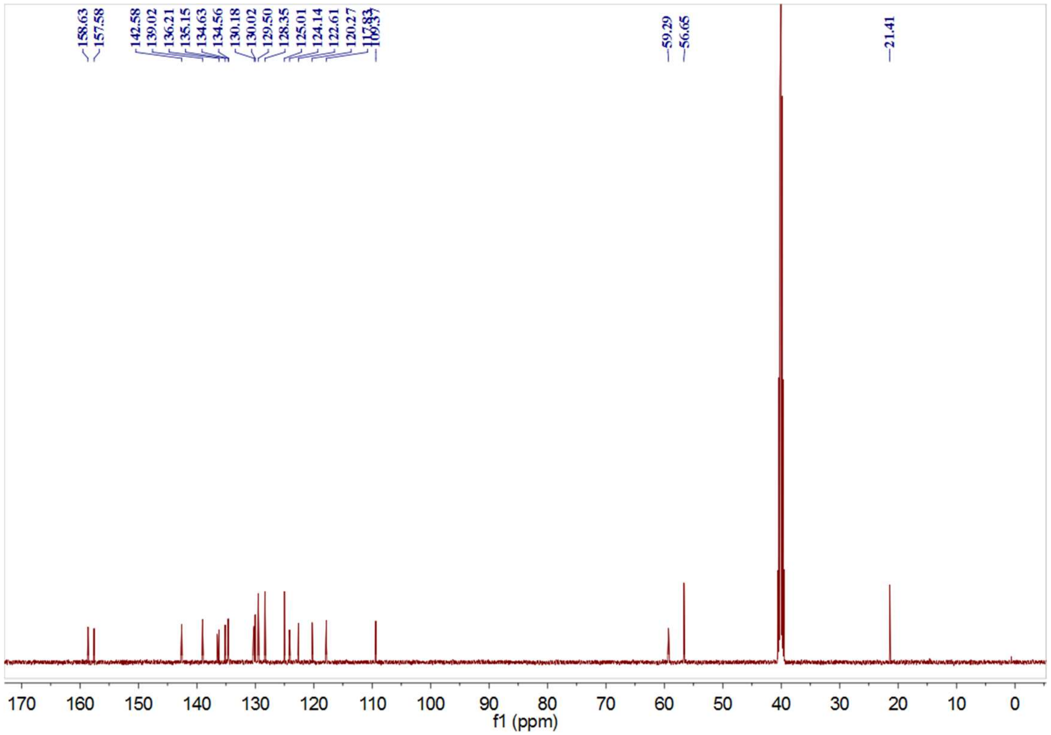
158.63
157.58
142.58
139.02
136.21
135.15
134.63
134.56
130.18
130.02
129.50
128.35
125.01
124.14
122.61
120.27
117.83
109.37
59.29
56.65
21.41
170 160 150 140 130 120 110 100 90 80 70 60 50 40 30 20 10 0
f1 (ppm)

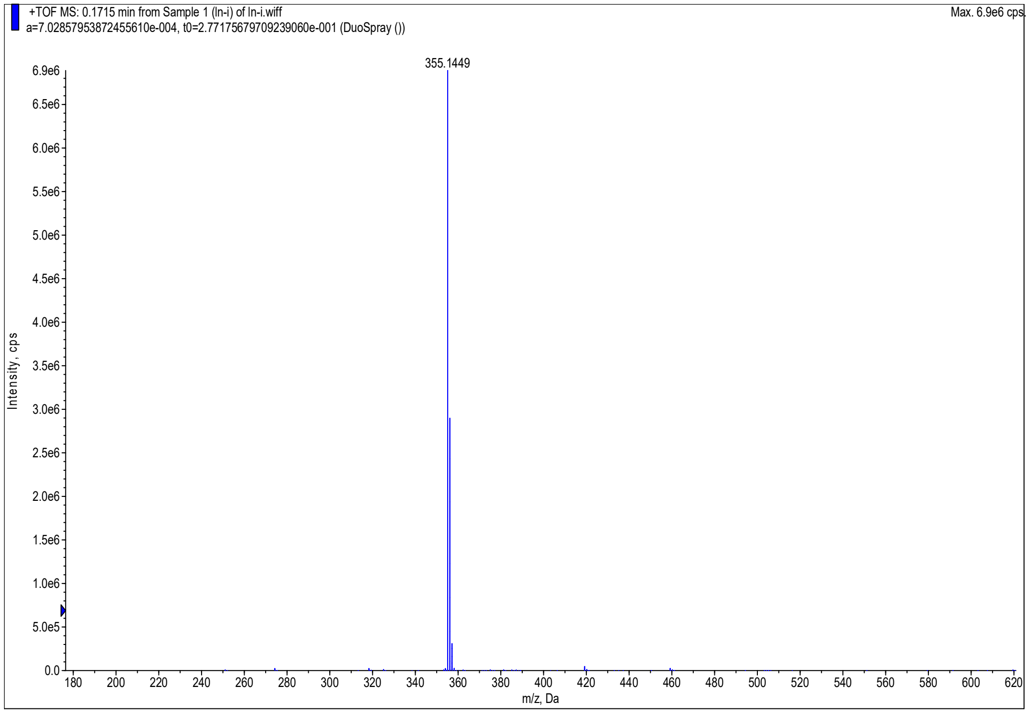

+TOF MS: 0.1715 min from Sample 1 (In-i) of In-i.wiff
a=7.02857953872455610e-004, t0=2.77175679709239060e-001 (DuoSpray ())
Max. 6.9e6 cps.
355.1449
Intensity, cps
m/z, Da

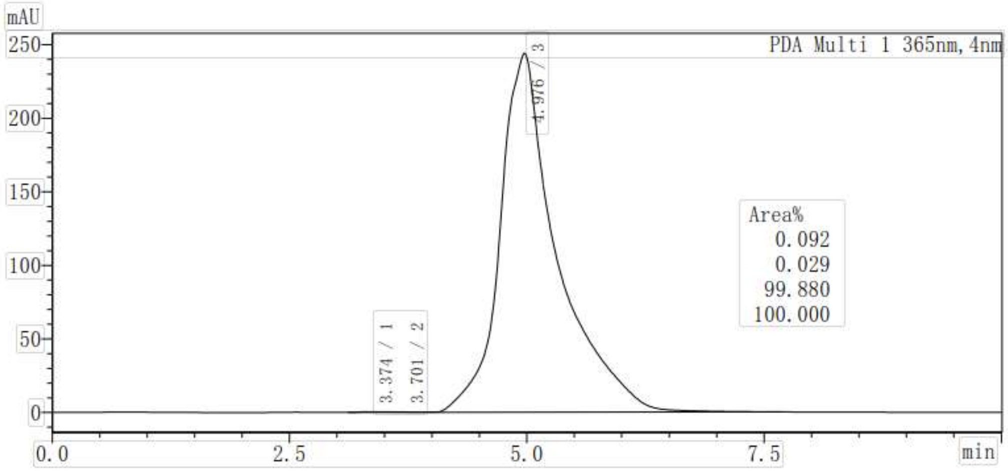

Figure S10. The NMR, HRMS and HPLC spectra of compound **6i**.

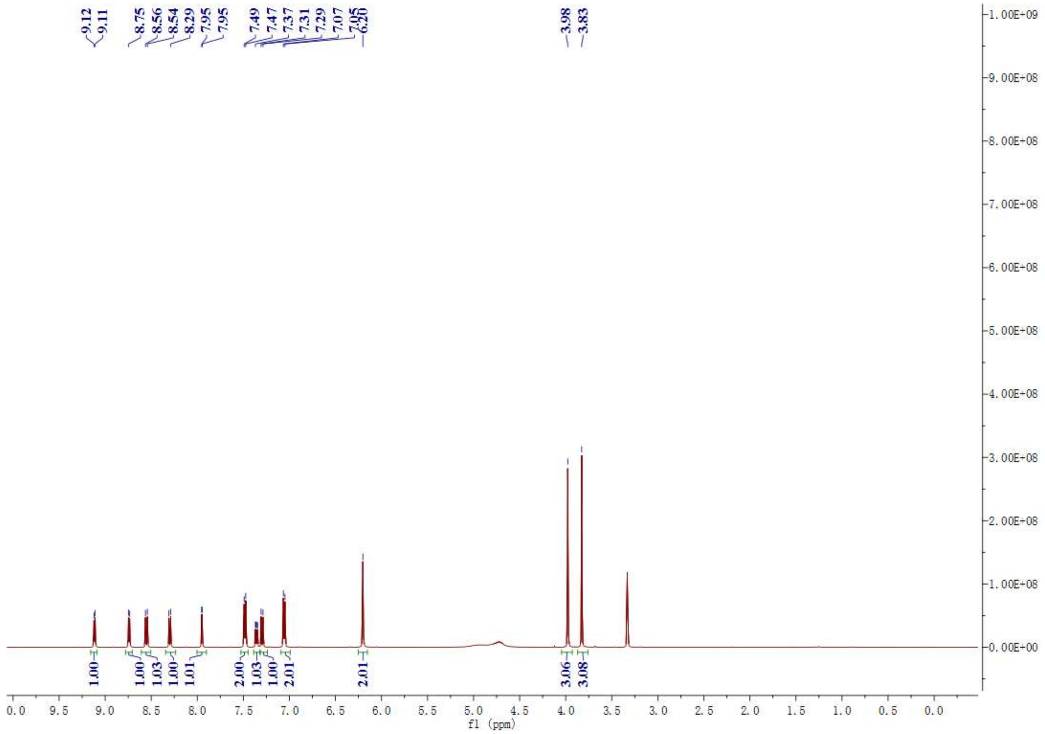

9.12
9.11
8.75
8.56
8.54
8.29
7.95
7.95
7.49
7.47
7.37
7.31
7.29
7.07
7.05
6.20
3.98
3.83
1.00
1.00
1.03
1.00
1.01
2.00
1.03
1.00
2.01
2.01
3.06
3.08
1.00E+09
9.00E+08
8.00E+08
7.00E+08
6.00E+08
5.00E+08
4.00E+08
3.00E+08
2.00E+08
1.00E+08
0.00E+00
f1 (ppm)

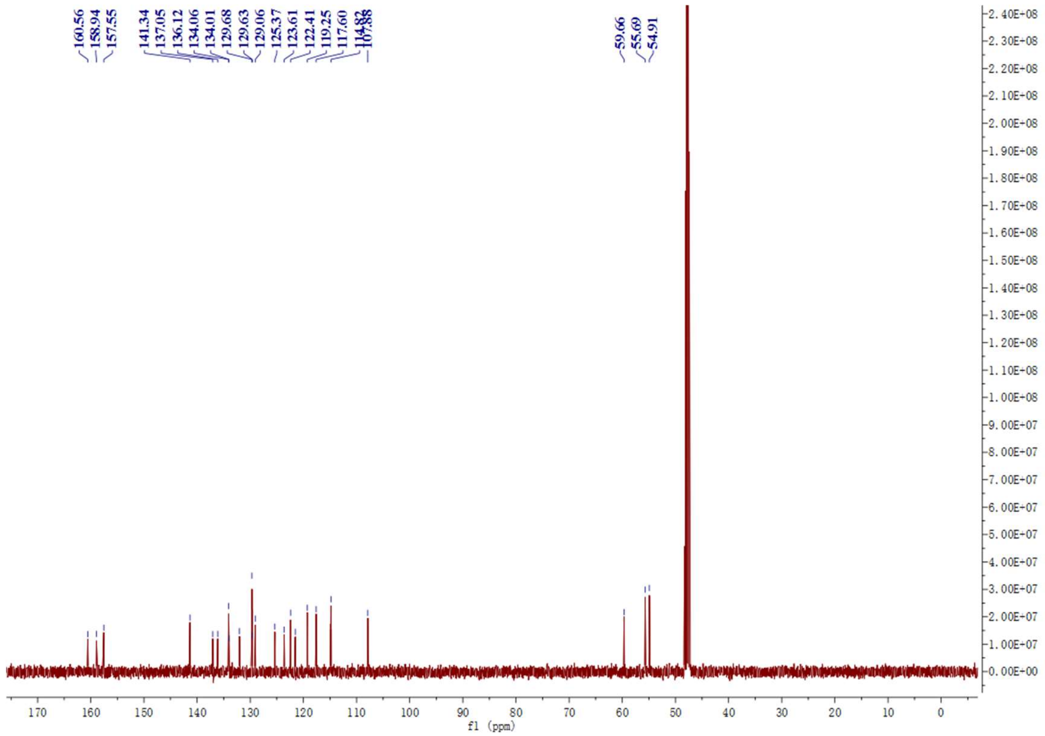

160.56
158.94
157.55
141.34
137.05
136.12
134.06
134.01
129.68
129.63
129.06
125.37
123.61
122.41
119.25
117.60
114.82
107.88
59.66
55.69
54.91
f1 (ppm)

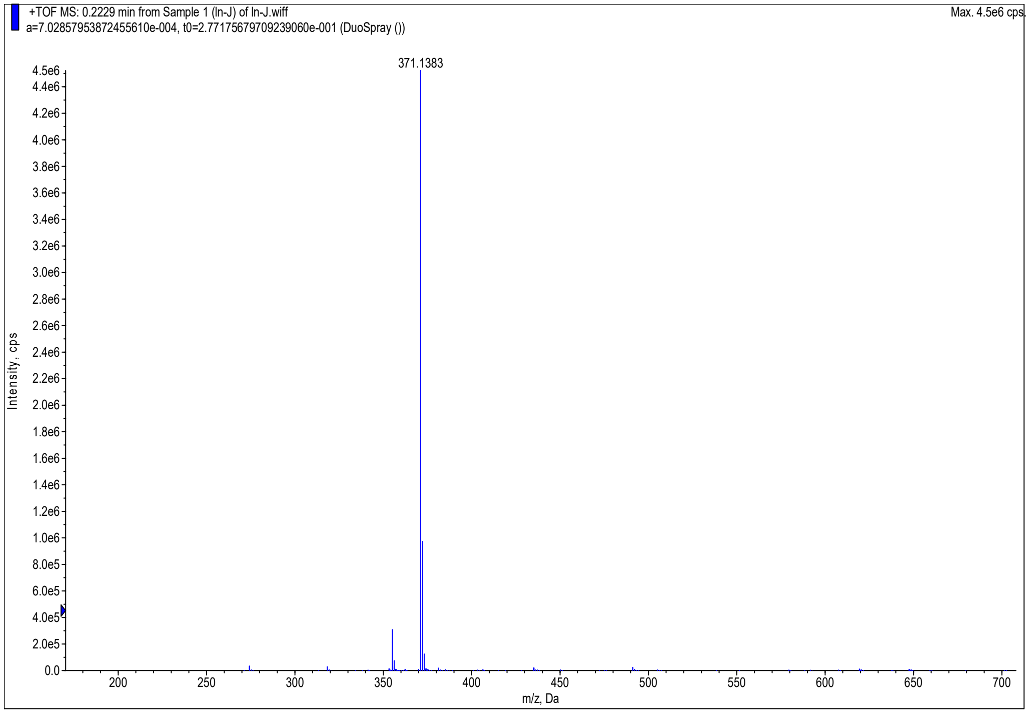

+TOF MS: 0.2229 min from Sample 1 (In-J) of In-J.wiff
a=7.02857953872455610e-004, t0=2.77175679709239060e-001 (DuoSpray ())
Max. 4.5e6 cps.
371.1383
Intensity, cps
m/z, Da

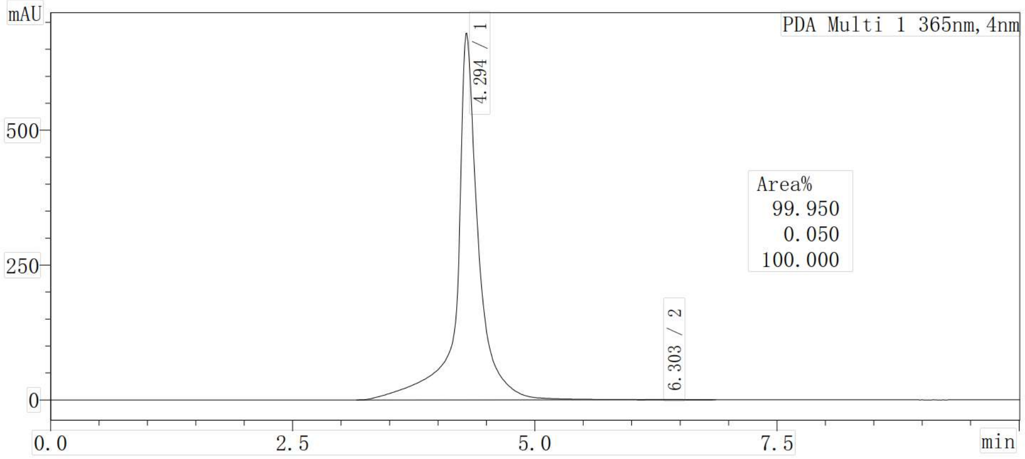

Figure S11. The NMR, HRMS and HPLC spectra of compound **6j**.

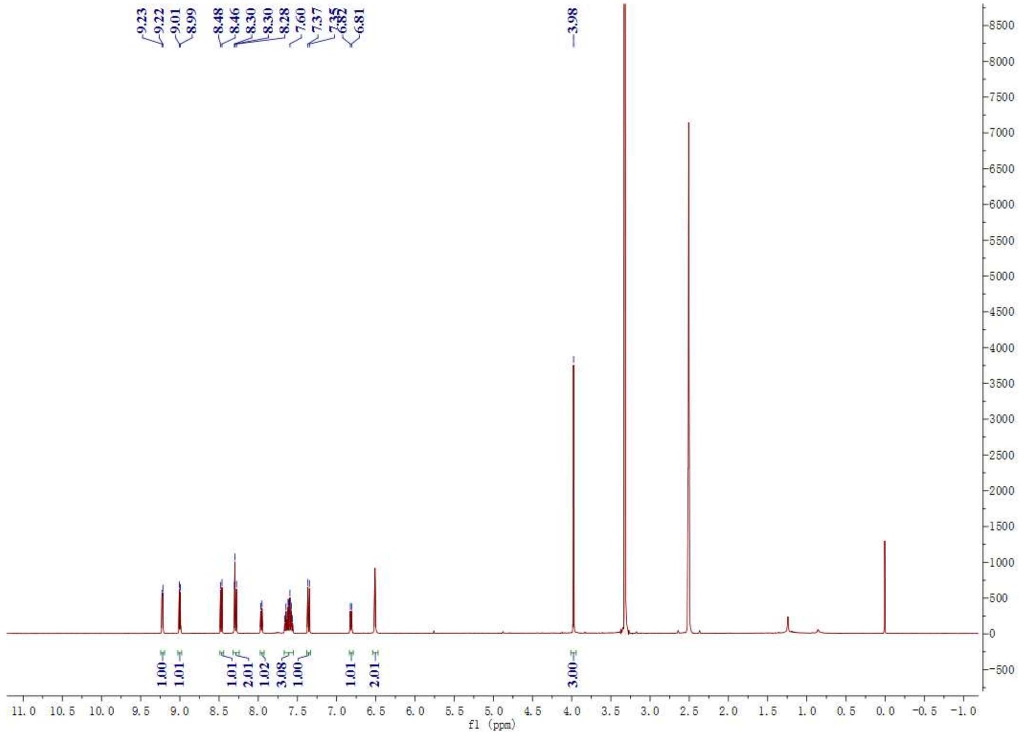

9.23
9.22
9.01
8.99
8.48
8.46
8.30
8.30
8.28
7.60
7.37
7.35
6.82
6.81
3.98
1.00
1.01
1.01
2.01
1.02
3.08
1.00
1.01
2.01
3.00
f1 (ppm)

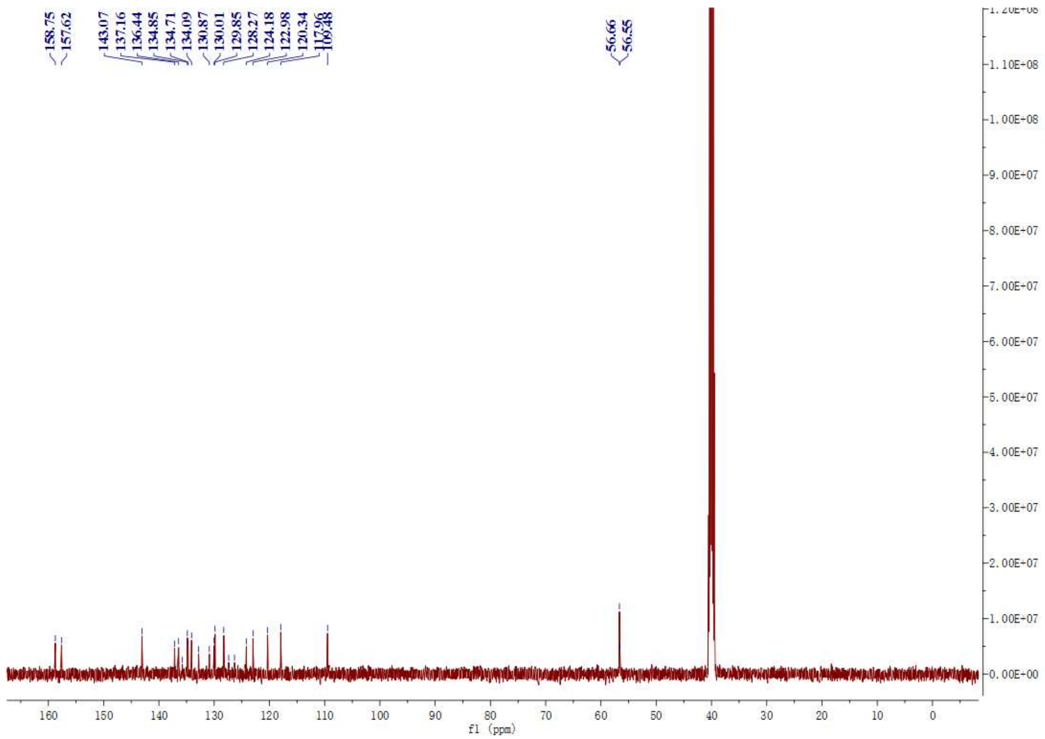

158.75
157.62
143.07
137.16
136.44
134.85
134.71
134.09
130.87
130.01
129.85
128.27
124.18
122.98
120.34
117.96
109.48
56.66
56.55
1.10E+08
1.00E+08
9.00E+07
8.00E+07
7.00E+07
6.00E+07
5.00E+07
4.00E+07
3.00E+07
2.00E+07
1.00E+07
0.00E+00
160
150
140
130
120
110
100
90
80
70
60
50
40
30
20
10
0
f1 (ppm)

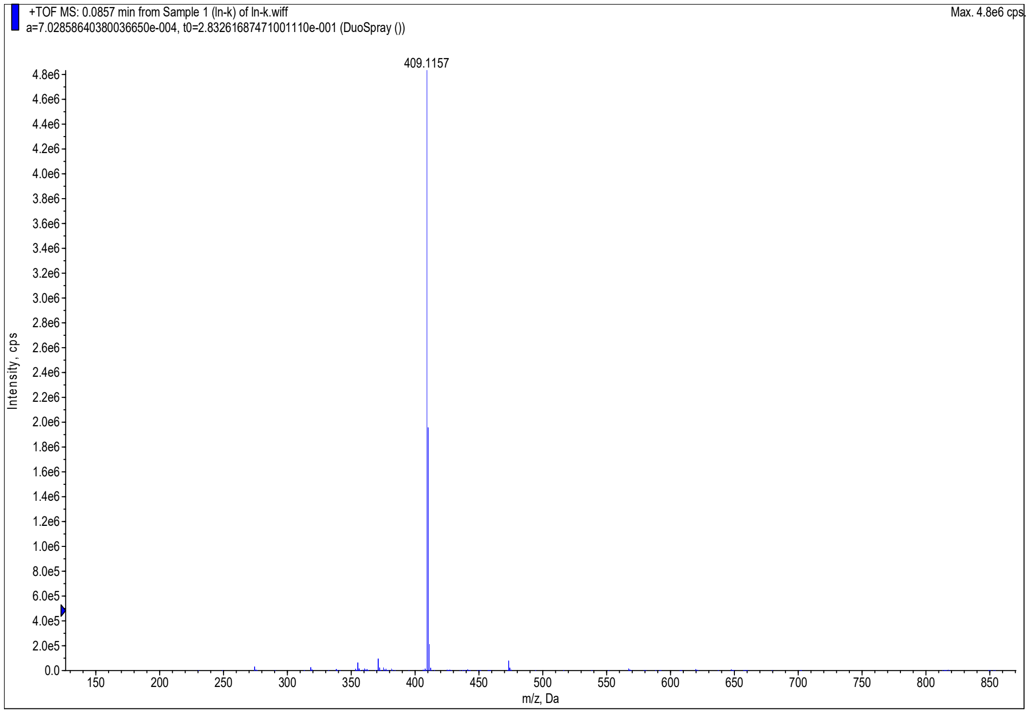

+TOF MS: 0.0857 min from Sample 1 (ln-k) of ln-k.wiff
a=7.02858640380036650e-004, t0=2.83261687471001110e-001 (DuoSpray ())
Max. 4.8e6 cps.
409.1157
Intensity, cps
m/z, Da

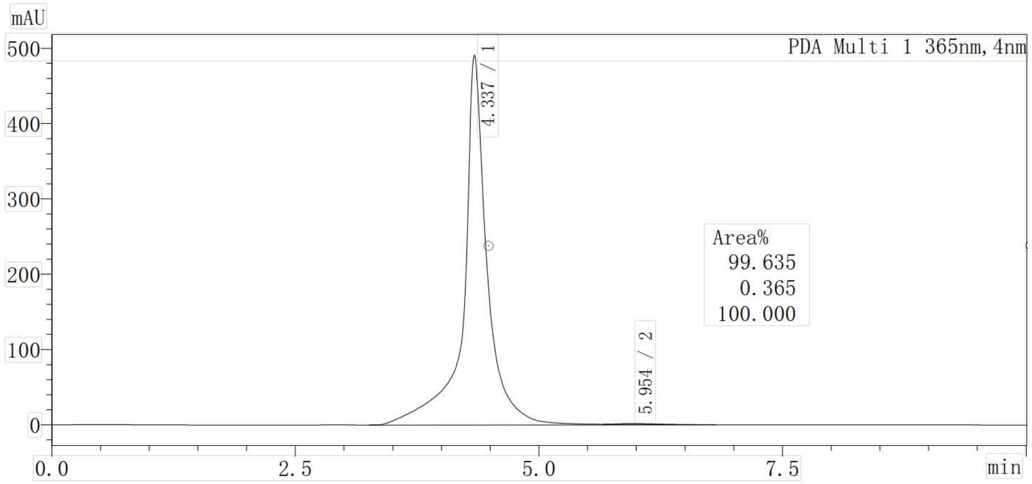

Figure S12. The NMR, HRMS and HPLC spectra of compound **6k**.

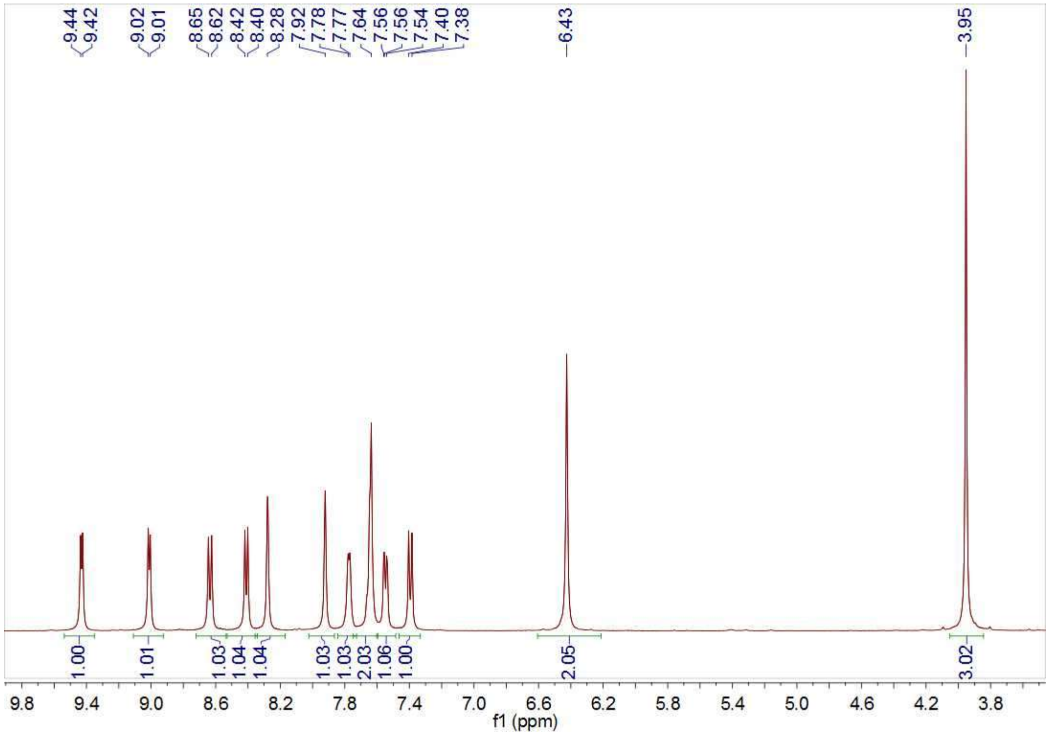

9.44
9.42
9.02
9.01
8.65
8.62
8.42
8.40
8.28
7.92
7.78
7.77
7.64
7.56
7.56
7.54
7.40
7.38
6.43
3.95
1.00
1.01
1.03
1.04
1.04
1.03
1.03
2.03
1.06
1.00
2.05
3.02
9.8
9.4
9.0
8.6
8.2
7.8
7.4
7.0
6.6
6.2
5.8
5.4
5.0
4.6
4.2
3.8
f1 (ppm)

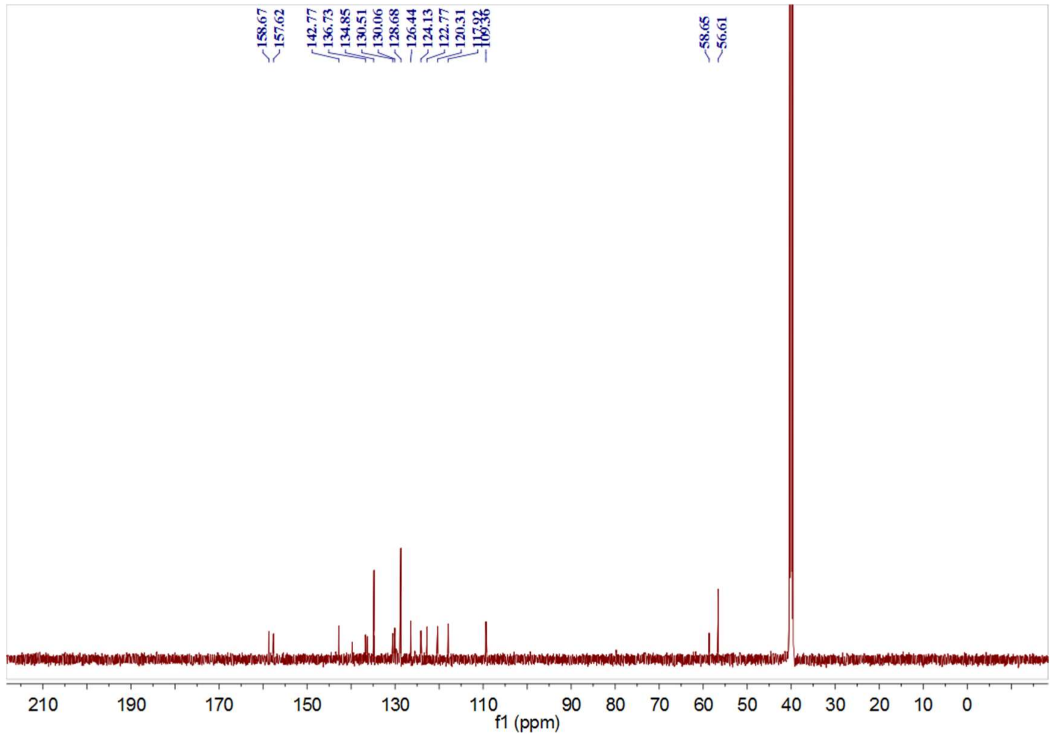

158.67
157.62
142.77
136.73
134.85
130.51
130.06
128.68
126.44
124.13
122.77
120.31
117.92
109.36
58.65
56.61
210
190
170
150
130
110
90
80
70
60
50
40
30
20
10
0
f1 (ppm)

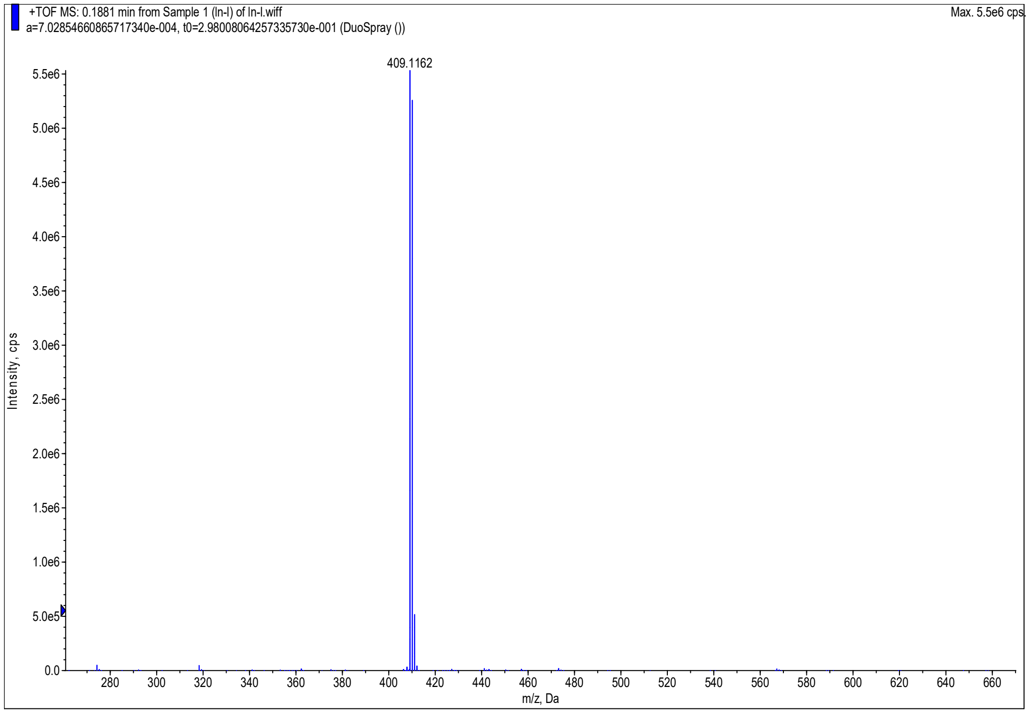
+TOF MS: 0.1881 min from Sample 1 (In-I) of In-I.wiff
a=7.02854660865717340e-004, t0=2.98008064257335730e-001 (DuoSpray ())
Max. 5.5e6 cps.
409.1162
Intensity, cps
5.5e6
5.0e6
4.5e6
4.0e6
3.5e6
3.0e6
2.5e6
2.0e6
1.5e6
1.0e6
5.0e5
0.0
280
300
320
340
360
380
400
420
440
460
480
500
520
540
560
580
600
620
640
660
m/z, Da

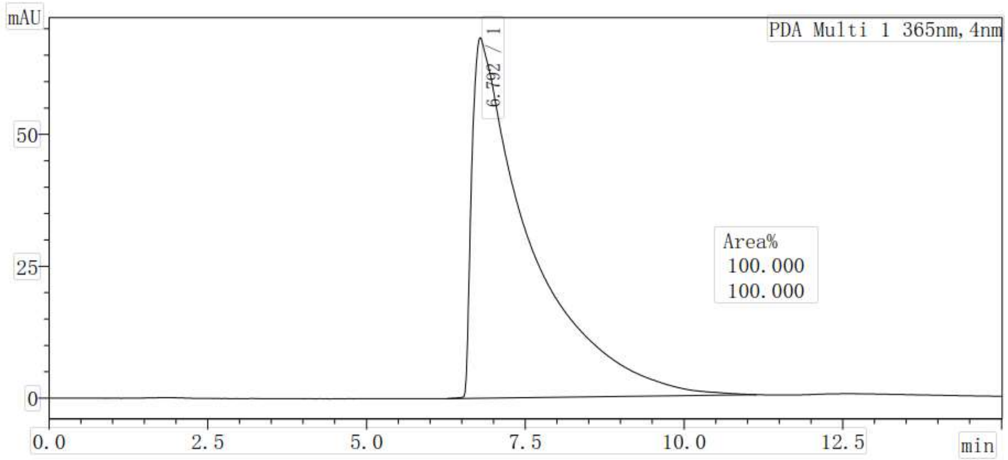

Figure S13. The NMR, HRMS and HPLC spectra of compound **6l**.

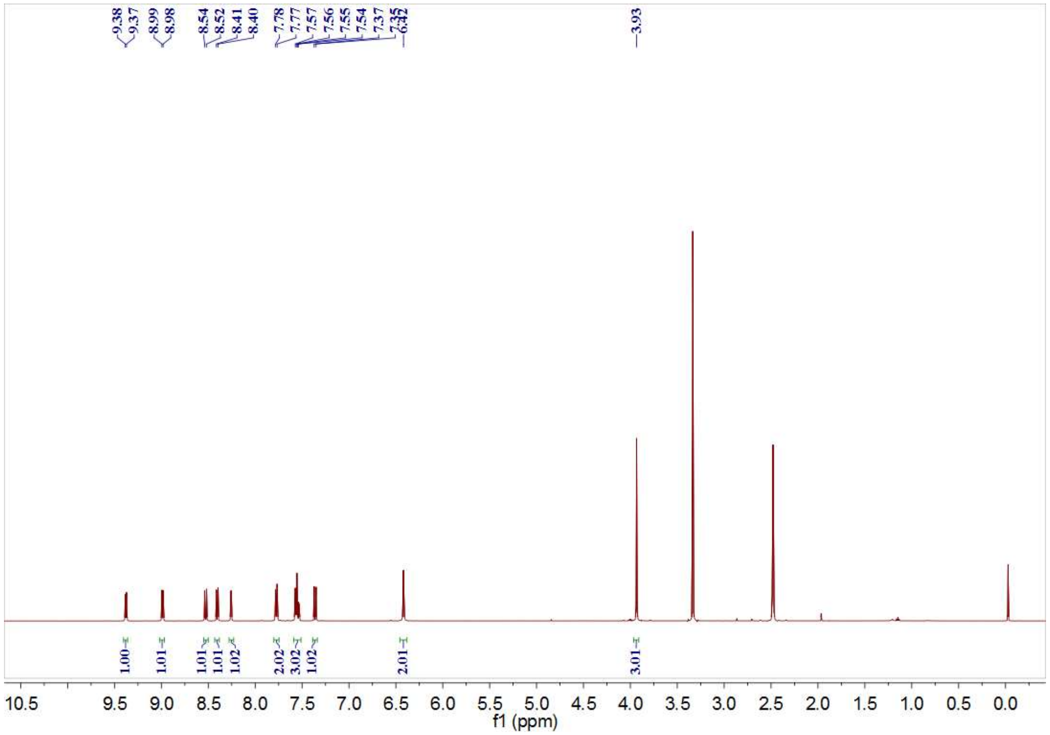
9.38
9.37
8.99
8.98
8.54
8.52
8.41
8.40
7.78
7.77
7.57
7.56
7.55
7.54
7.37
7.35
6.42
3.93
1.00
1.01
1.01
1.01
1.02
2.02
3.02
1.02
2.01
3.01
10.5
9.5
9.0
8.5
8.0
7.5
7.0
6.5
6.0
5.5
5.0
4.5
4.0
3.5
3.0
2.5
2.0
1.5
1.0
0.5
0.0
f1 (ppm)

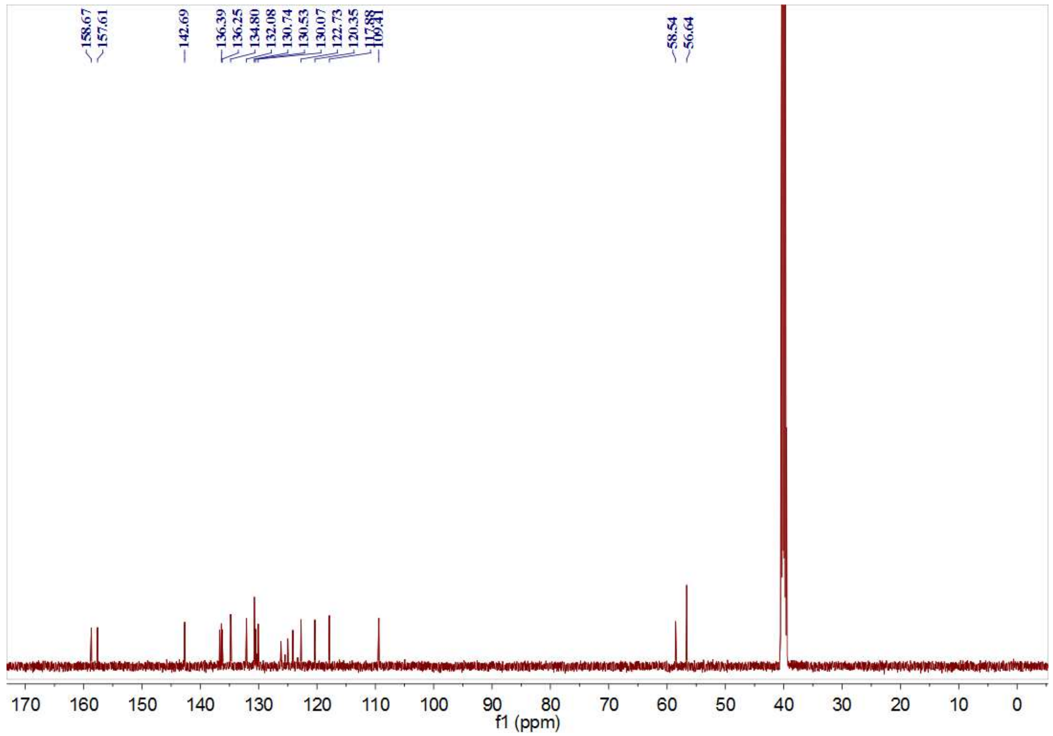

158.67
157.61
142.69
136.39
136.25
134.80
132.08
130.74
130.53
130.07
122.73
120.35
117.88
109.41
58.54
56.64
170
160
150
140
130
120
110
100
90
80
70
60
50
40
30
20
10
0
f1 (ppm)

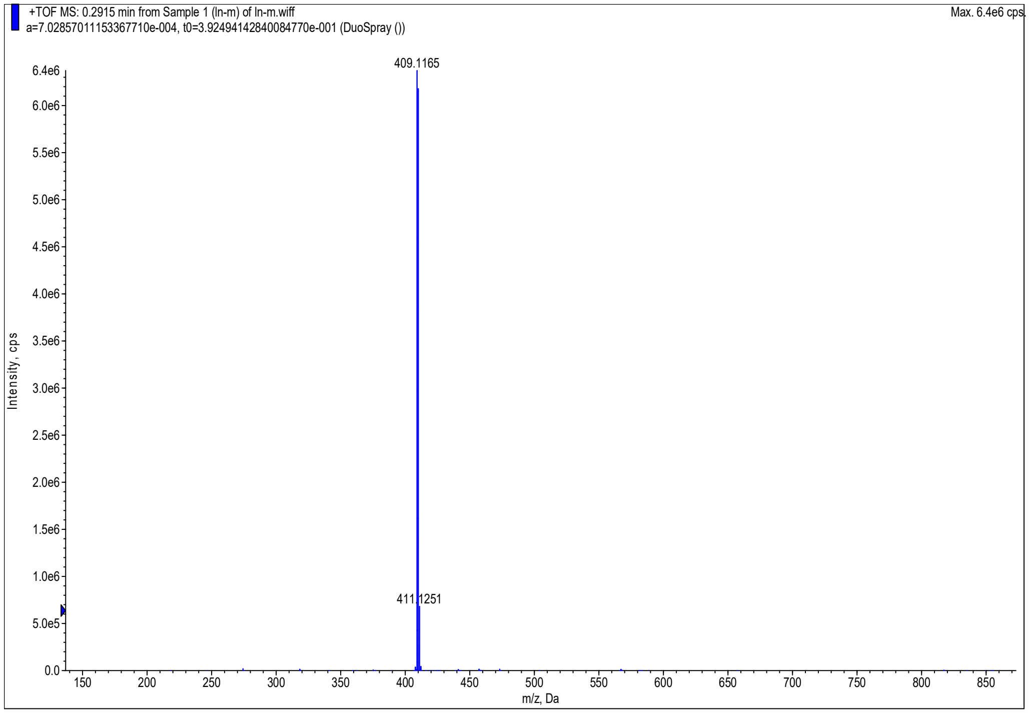

+TOF MS: 0.2915 min from Sample 1 (ln-m) of ln-m.wiff
a=7.02857011153367710e-004, t0=3.92494142840084770e-001 (DuoSpray ())
Max. 6.4e6 cps.
409.1165
411.1251
Intensity, cps
m/z, Da

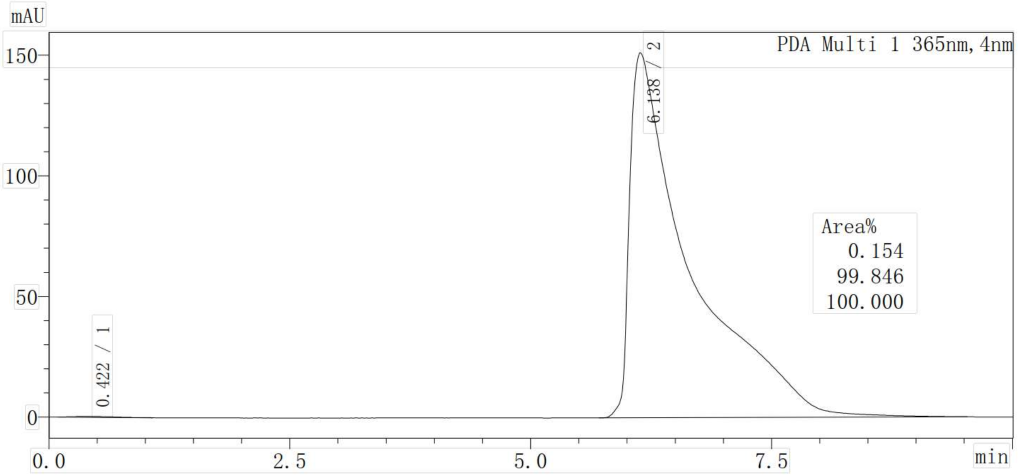

Figure S14. The NMR, HRMS and HPLC spectra of compound **6m**.

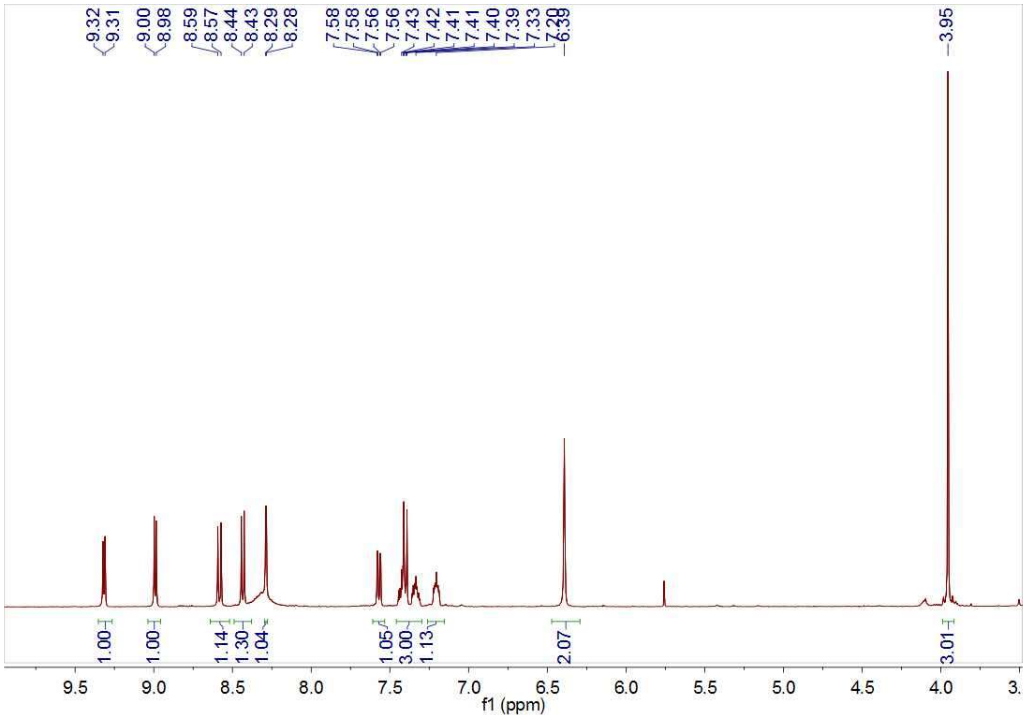
9.32
9.31
9.00
8.98
8.59
8.57
8.44
8.43
8.29
8.28
7.58
7.58
7.56
7.56
7.43
7.42
7.41
7.41
7.40
7.39
7.33
7.20
6.39
3.95
1.00
1.00
1.14
1.30
1.04
1.05
3.00
1.13
2.07
3.01
9.5
9.0
8.5
8.0
7.5
7.0
6.5
6.0
5.5
5.0
4.5
4.0
f1 (ppm)

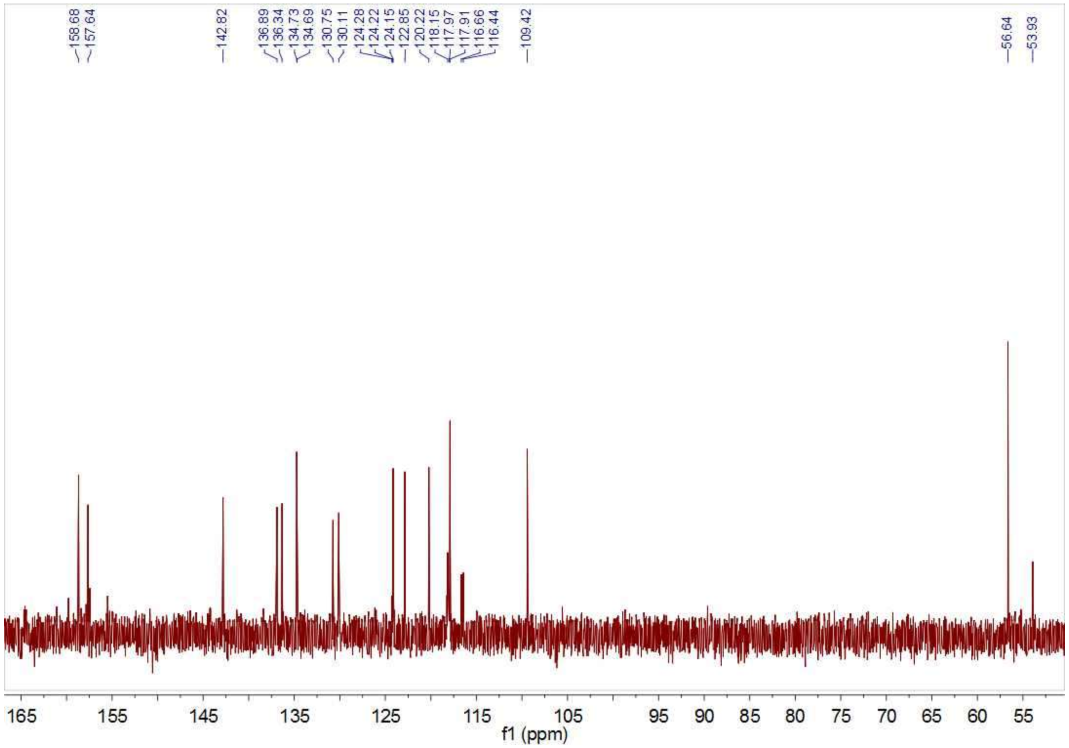
158.68
157.64
142.82
136.89
136.34
134.73
134.69
130.75
130.11
124.28
124.22
124.15
122.85
120.22
118.15
117.97
117.91
116.66
116.44
109.42
56.64
53.93
165
155
145
135
125
115
105
95
90
85
80
75
70
65
60
55
f1 (ppm)

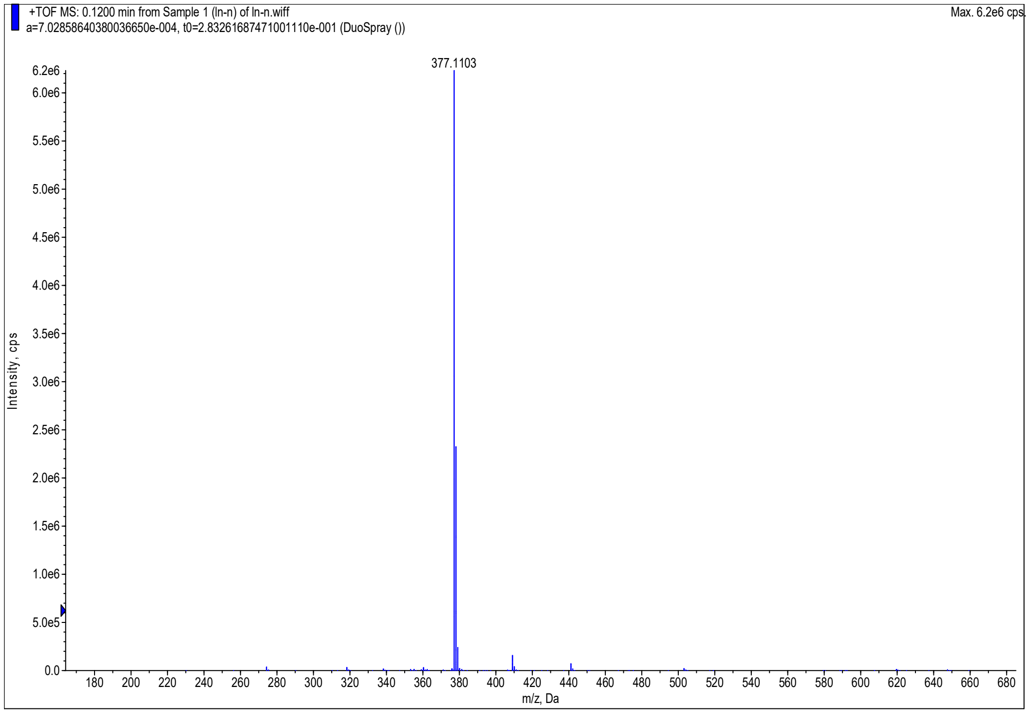
+TOF MS: 0.1200 min from Sample 1 (ln-n) of ln-n.wiff
a=7.02858640380036650e-004, t0=2.83261687471001110e-001 (DuoSpray ())
Max. 6.2e6 cps.
377.1103
Intensity, cps
m/z, Da

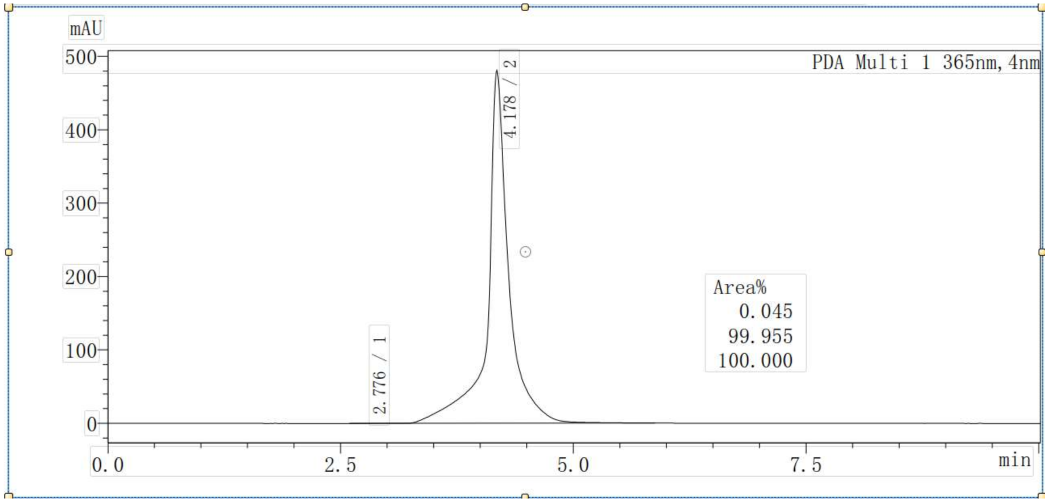

Figure S15. The NMR, HRMS and HPLC spectra of compound **6n**.

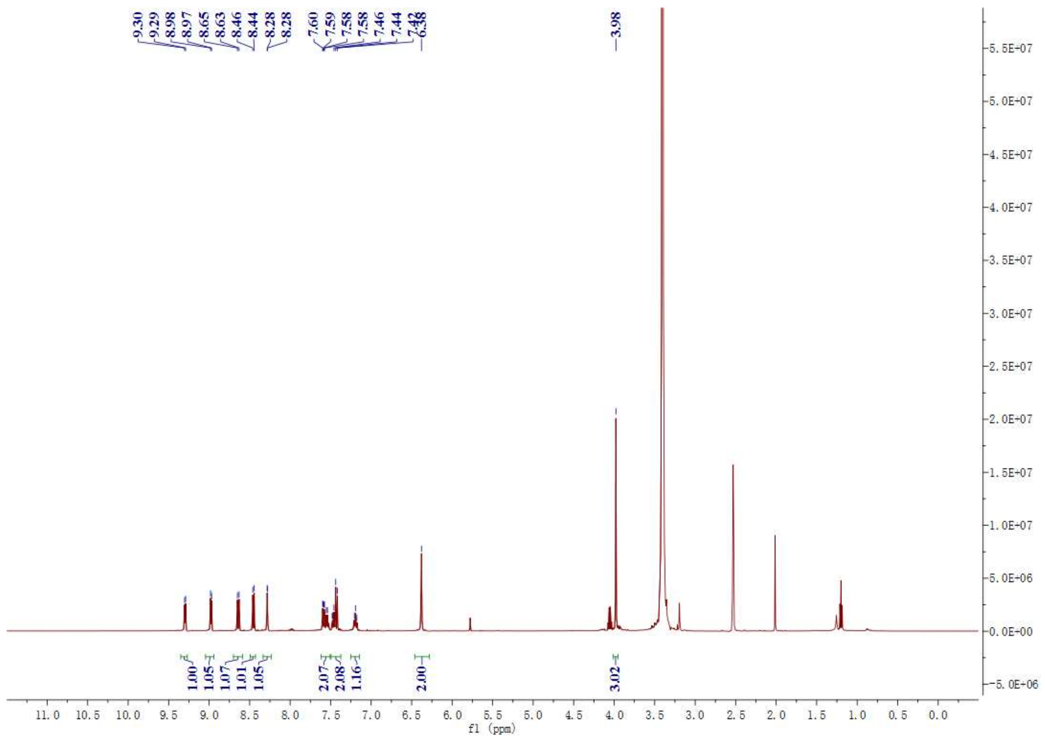

9.30
9.29
8.98
8.97
8.65
8.63
8.46
8.44
8.28
8.28
7.60
7.59
7.58
7.58
7.46
7.44
7.42
6.38
3.98
1.00
1.05
1.07
1.01
1.05
2.07
2.08
1.16
2.00
3.02
11.0 10.5 10.0 9.5 9.0 8.5 8.0 7.5 7.0 6.5 6.0 5.5 5.0 4.5 4.0 3.5 3.0 2.5 2.0 1.5 1.0 0.5 0.0
f1 (ppm)
5.5E+07
5.0E+07
4.5E+07
4.0E+07
3.5E+07
3.0E+07
2.5E+07
2.0E+07
1.5E+07
1.0E+07
5.0E+06
0.0E+00
-5.0E+06

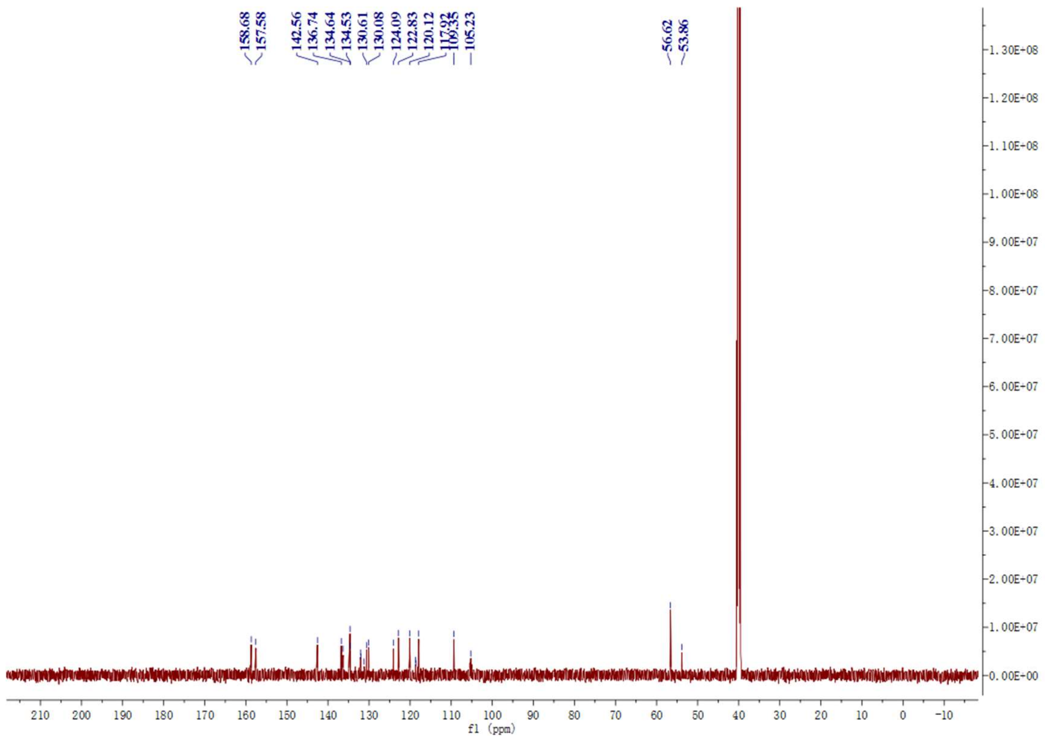
158.68
157.58
142.56
136.74
134.64
134.53
130.61
130.08
124.09
122.83
120.12
117.92
109.35
105.23
56.62
53.86
f1 (ppm)

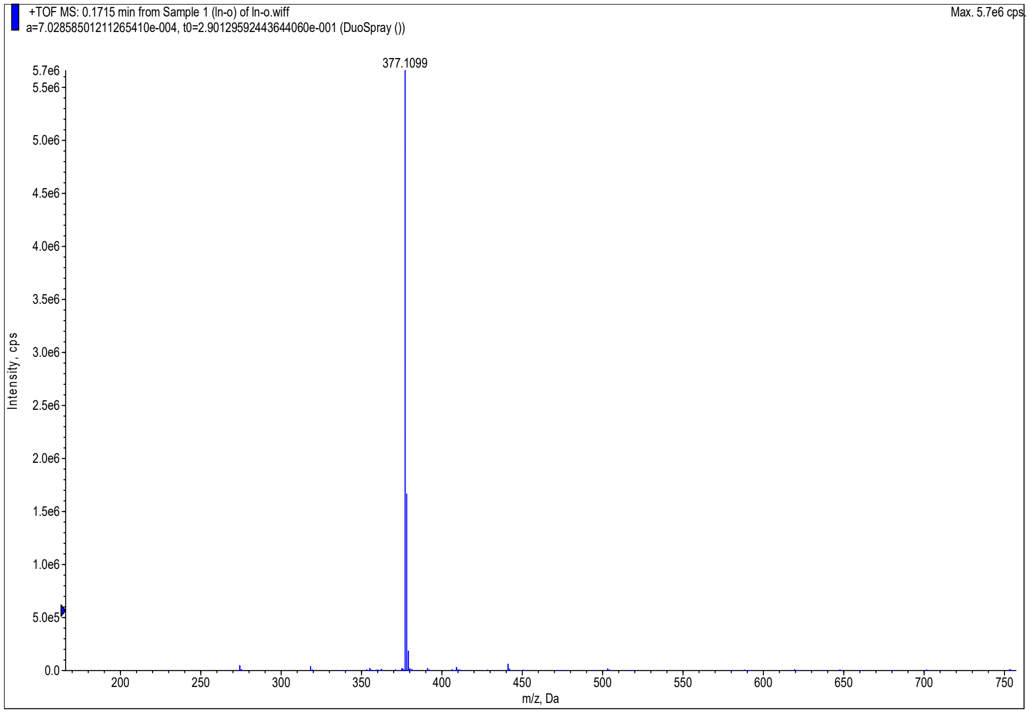
+TOF MS: 0.1715 min from Sample 1 (ln-o) of ln-o.wiff
a=7.02858501211265410e-004, t0=2.90129592443644060e-001 (DuoSpray ())
Max. 5.7e6 cps.
377.1099
Intensity, cps
5.7e6
5.5e6
5.0e6
4.5e6
4.0e6
3.5e6
3.0e6
2.5e6
2.0e6
1.5e6
1.0e6
5.0e5
0.0
200
250
300
350
400
450
500
550
600
650
700
750
m/z, Da

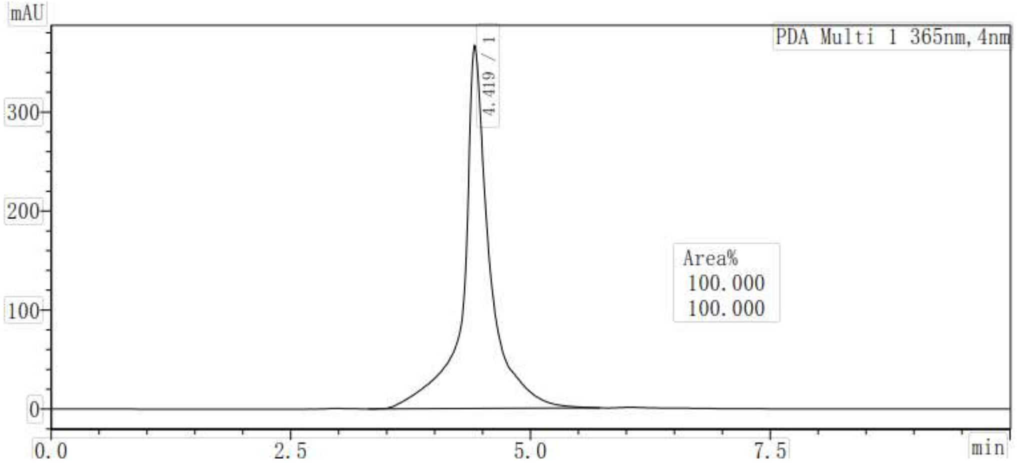

Figure S16. The NMR, HRMS and HPLC spectra of compound **6o**.

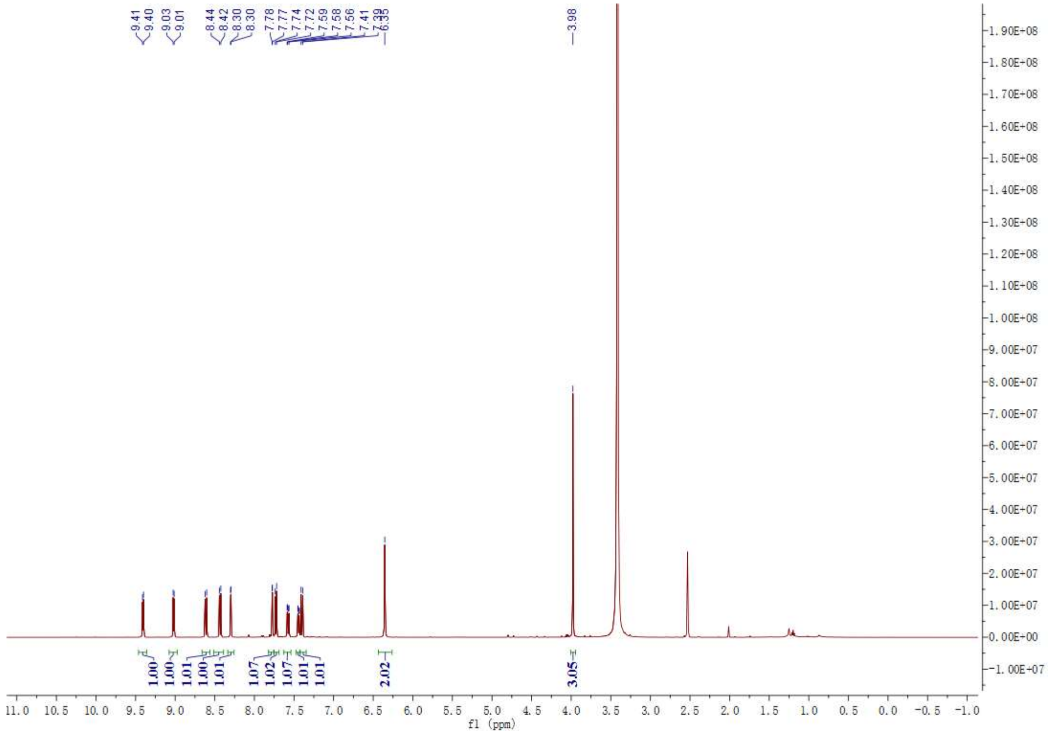

9.41
9.40
9.03
9.01
8.44
8.42
8.30
8.30
7.78
7.77
7.74
7.72
7.59
7.58
7.56
7.41
7.39
6.35
3.98
1.00
1.00
1.01
1.00
1.01
1.07
1.02
1.07
1.01
1.01
2.02
3.05
f1 (ppm)

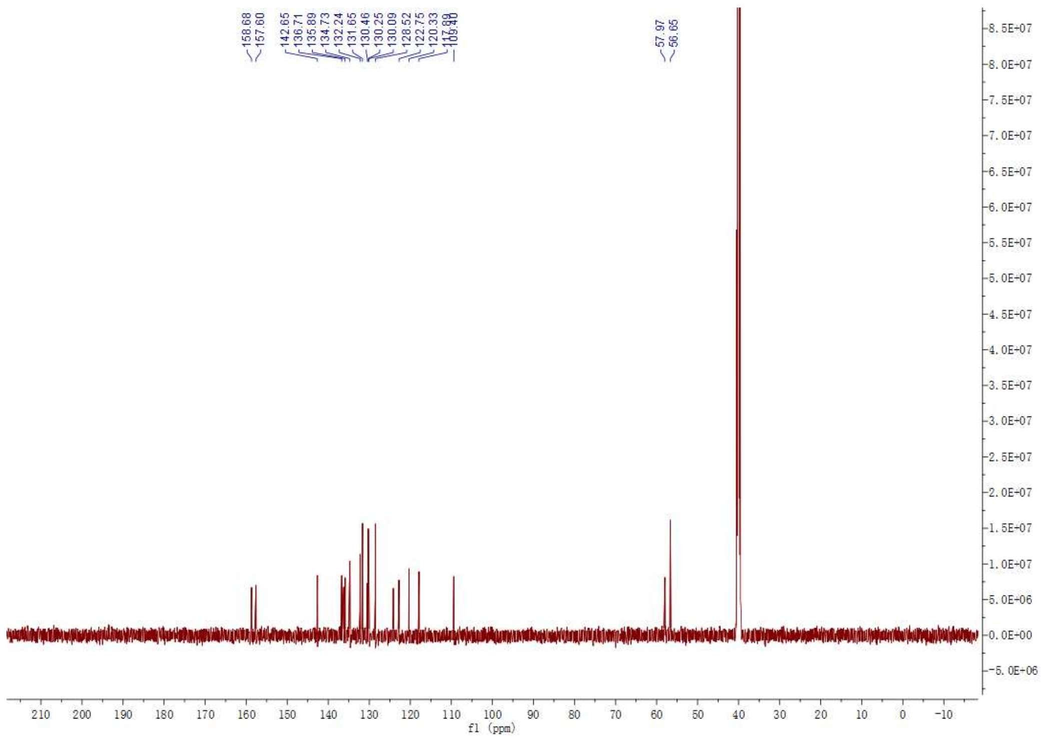
158.68
157.60
142.65
136.71
135.89
134.73
132.24
131.65
130.46
130.25
130.09
128.52
122.75
120.33
117.89
109.40
57.97
56.65
8.5E+07
8.0E+07
7.5E+07
7.0E+07
6.5E+07
6.0E+07
5.5E+07
5.0E+07
4.5E+07
4.0E+07
3.5E+07
3.0E+07
2.5E+07
2.0E+07
1.5E+07
1.0E+07
5.0E+06
0.0E+00
-5.0E+06
210 200 190 180 170 160 150 140 130 120 110 100 90 80 70 60 50 40 30 20 10 0 -10
f1 (ppm)

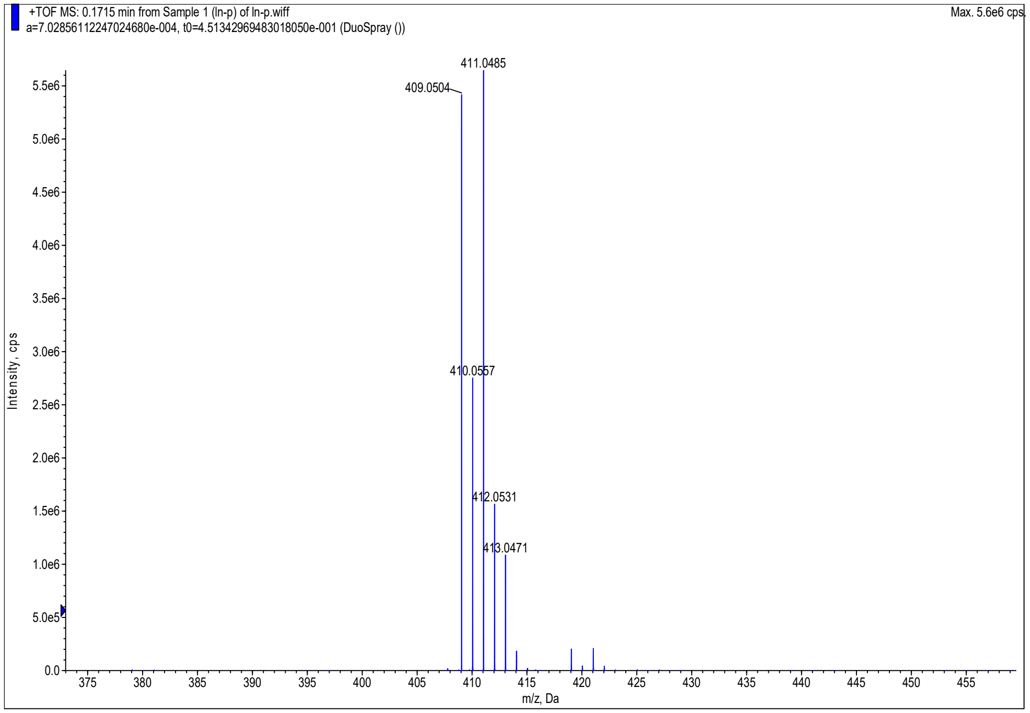
+TOF MS: 0.1715 min from Sample 1 (In-p) of In-p.wiff
a=7.02856112247024680e-004, t0=4.51342969483018050e-001 (DuoSpray ())
Max. 5.6e6 cps.
411.0485
409.0504
410.0557
412.0531
413.0471
Intensity, cps
m/z, Da

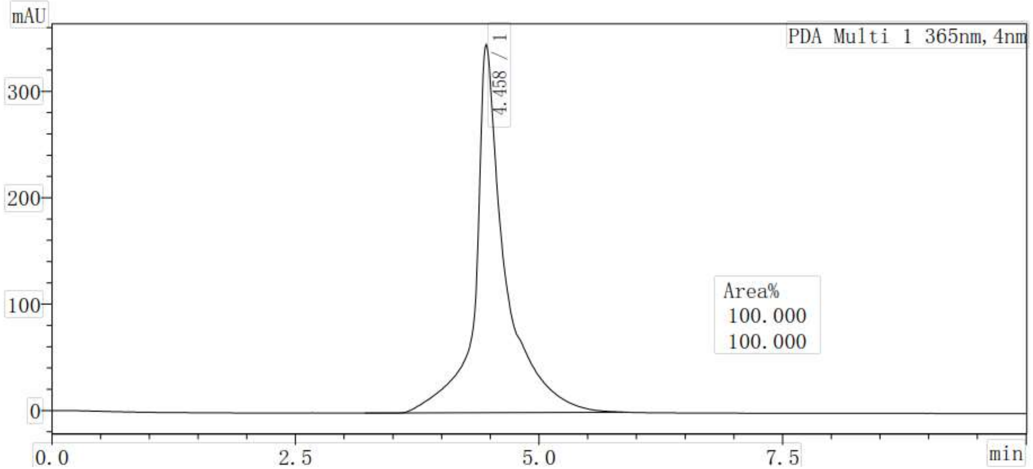

Figure S17. The NMR, HRMS and HPLC spectra of compound **6p**.

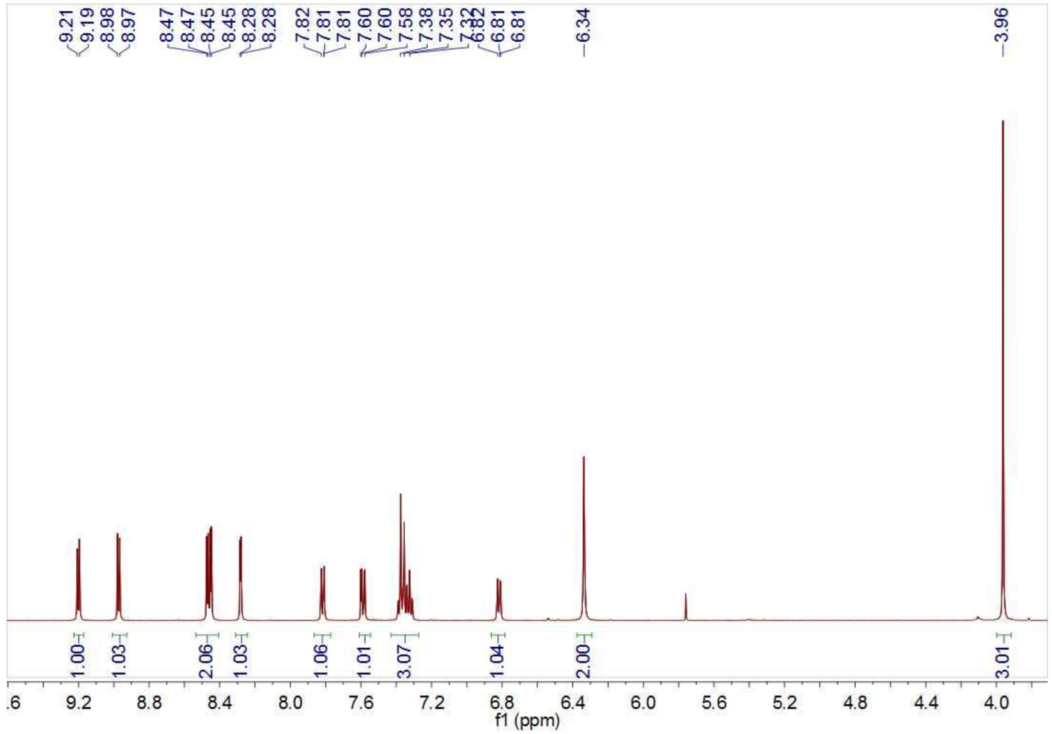
9.21
9.19
8.98
8.97
8.47
8.47
8.45
8.45
8.28
8.28
7.82
7.81
7.81
7.60
7.60
7.58
7.38
7.35
7.32
6.82
6.81
6.81
6.34
3.96
1.00
1.03
2.06
1.03
1.06
1.01
3.07
1.04
2.00
3.01
9.2
8.8
8.4
8.0
7.6
7.2
6.8
6.4
6.0
5.6
5.2
4.8
4.4
4.0
f1 (ppm)

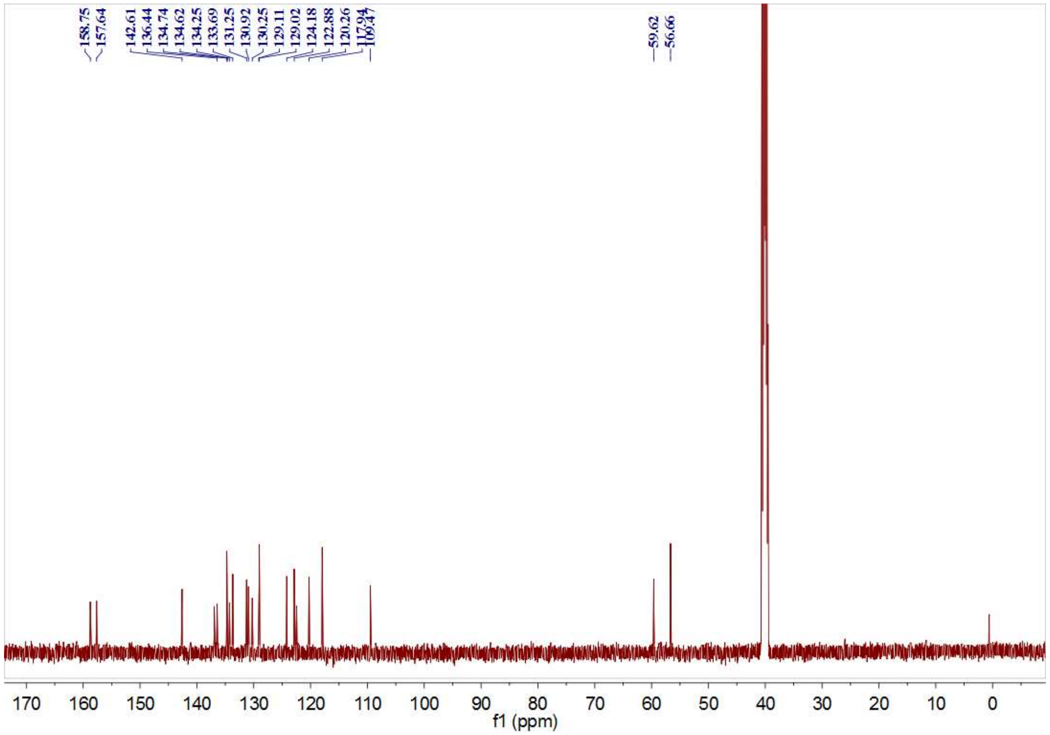
158.75
157.64
142.61
136.44
134.74
134.62
134.25
133.69
131.25
130.92
130.25
129.11
129.02
124.18
122.88
120.26
117.94
109.47
59.62
56.66
170 160 150 140 130 120 110 100 90 80 70 60 50 40 30 20 10 0
f1 (ppm)

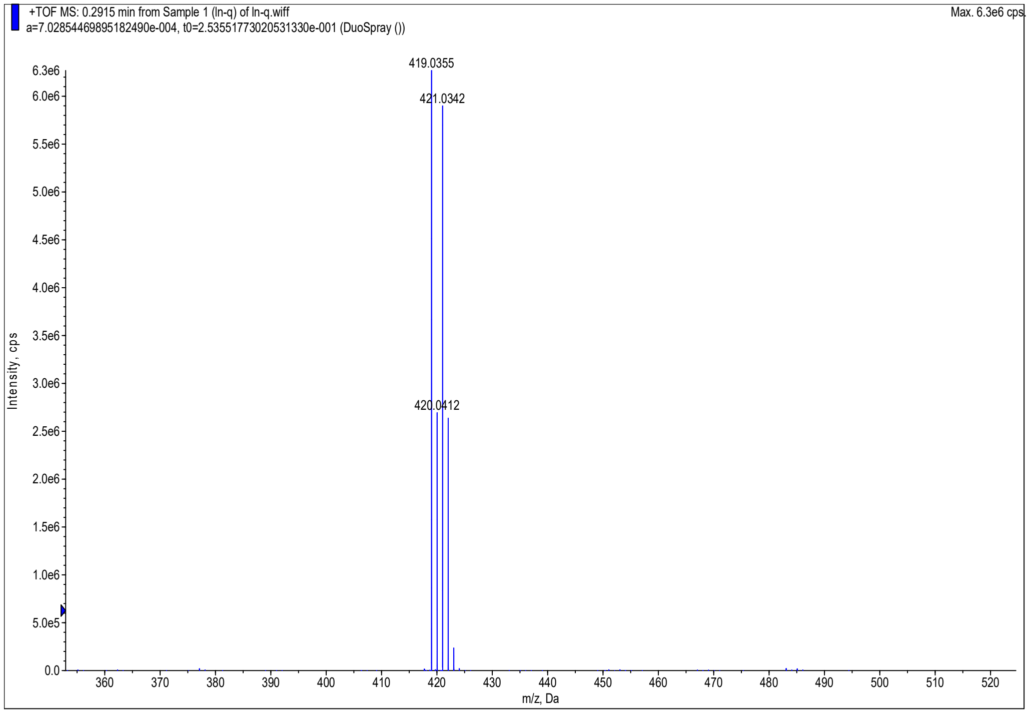

+TOF MS: 0.2915 min from Sample 1 (ln-q) of ln-q.wiff
a=7.02854469895182490e-004, t0=2.53551773020531330e-001 (DuoSpray ())
Max. 6.3e6 cps.
419.0355
421.0342
420.0412
Intensity, cps
m/z, Da

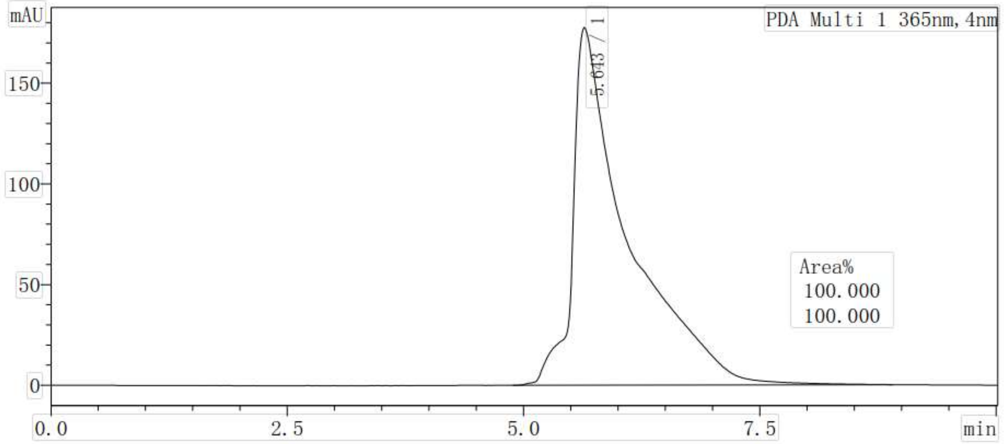

Figure S18. The NMR, HRMS and HPLC spectra of compound **6q**.

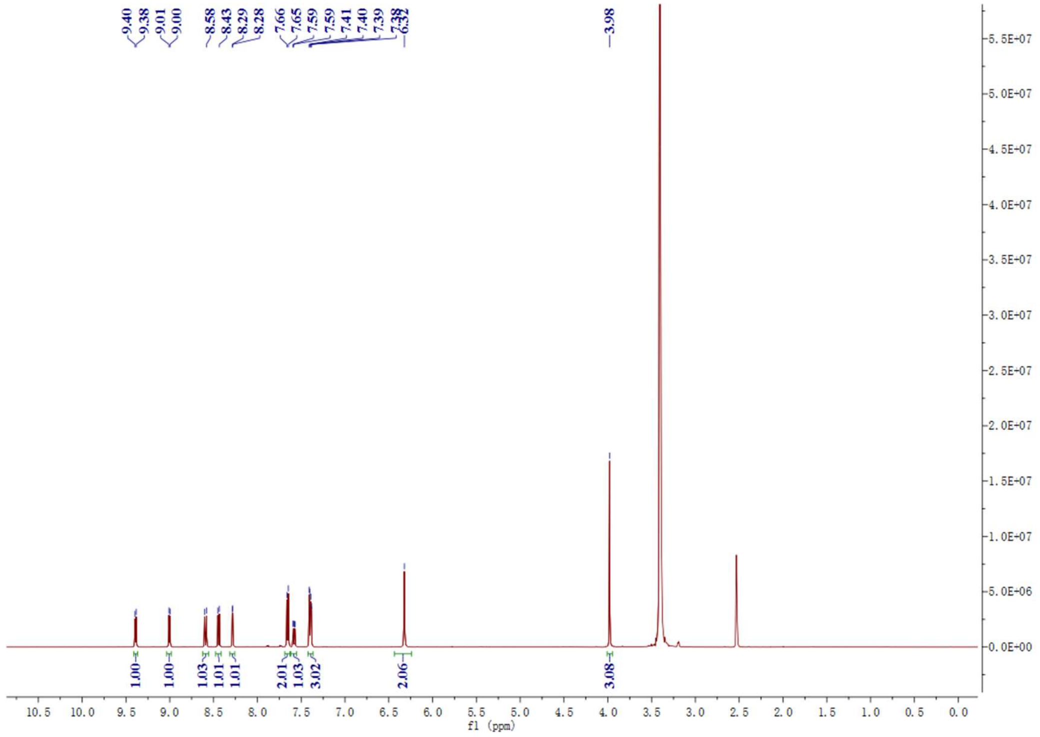
9.40
9.38
9.01
9.00
8.58
8.43
8.29
8.28
7.66
7.65
7.59
7.59
7.41
7.40
7.39
7.38
6.32
3.98
1.00
1.00
1.03
1.01
1.01
2.01
1.03
3.02
2.06
3.08
f1 (ppm)

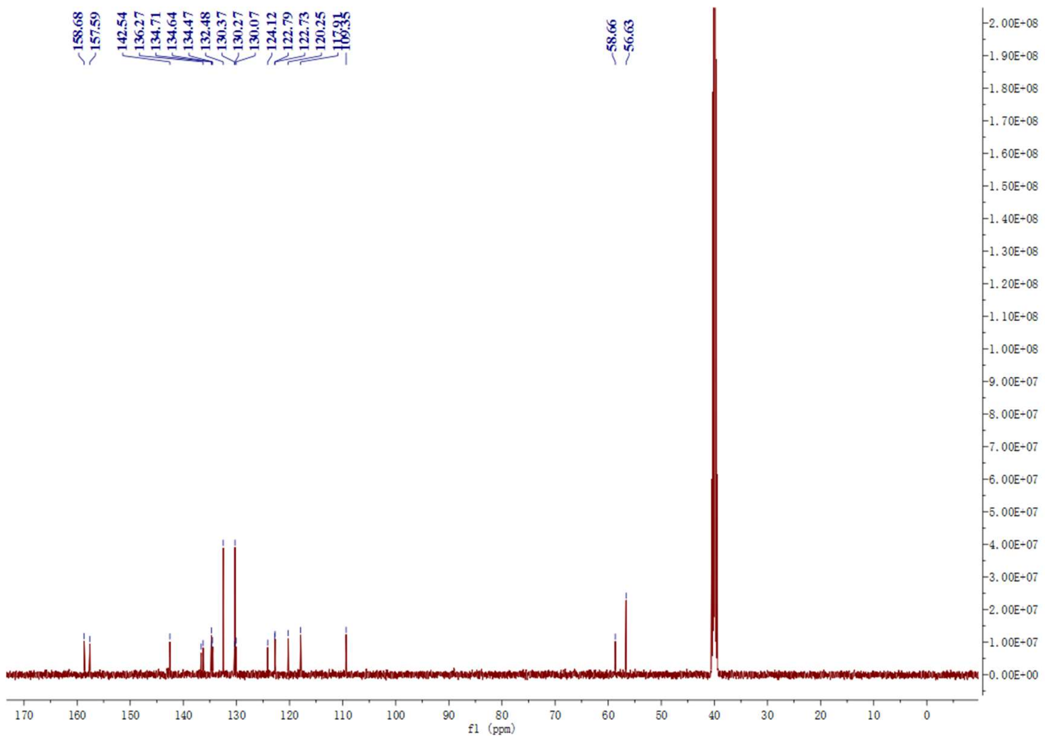
158.68
157.59
142.54
136.27
134.71
134.64
134.47
132.48
130.37
130.27
130.07
124.12
122.79
122.73
120.25
109.35
58.66
56.63
f1 (ppm)

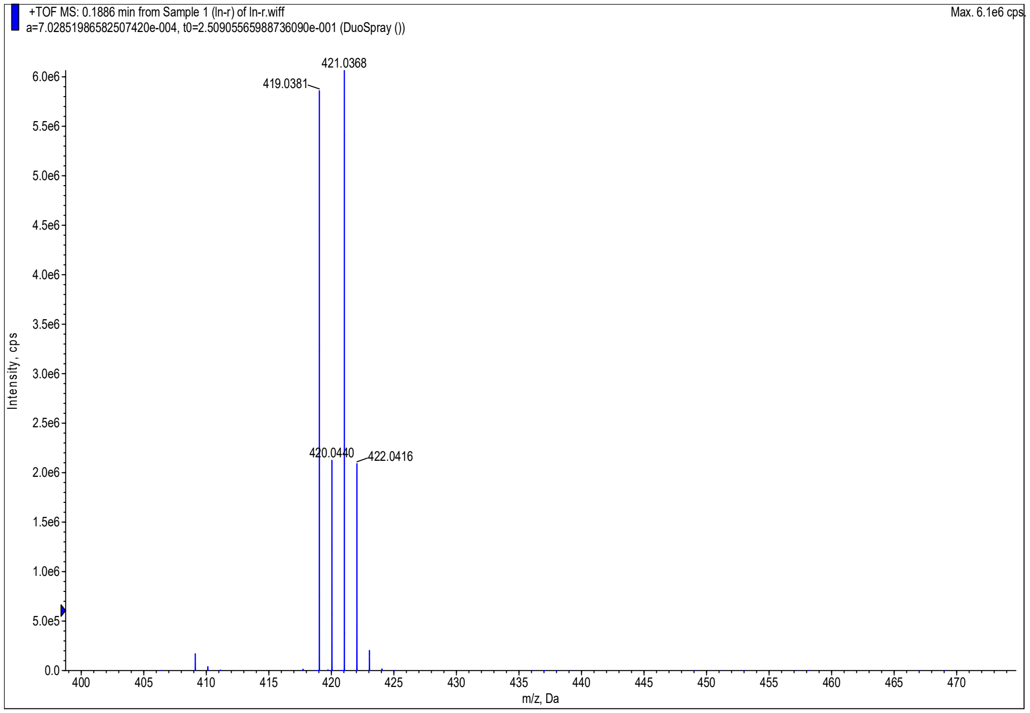
+TOF MS: 0.1886 min from Sample 1 (In-r) of In-r.wiff
a=7.02851986582507420e-004, t0=2.50905565988736090e-001 (DuoSpray ())
Max. 6.1e6 cps.
421.0368
419.0381
420.0440
422.0416
Intensity, cps
m/z, Da
6.0e6
5.5e6
5.0e6
4.5e6
4.0e6
3.5e6
3.0e6
2.5e6
2.0e6
1.5e6
1.0e6
5.0e5
0.0
400
405
410
415
420
425
430
435
440
445
450
455
460
465
470

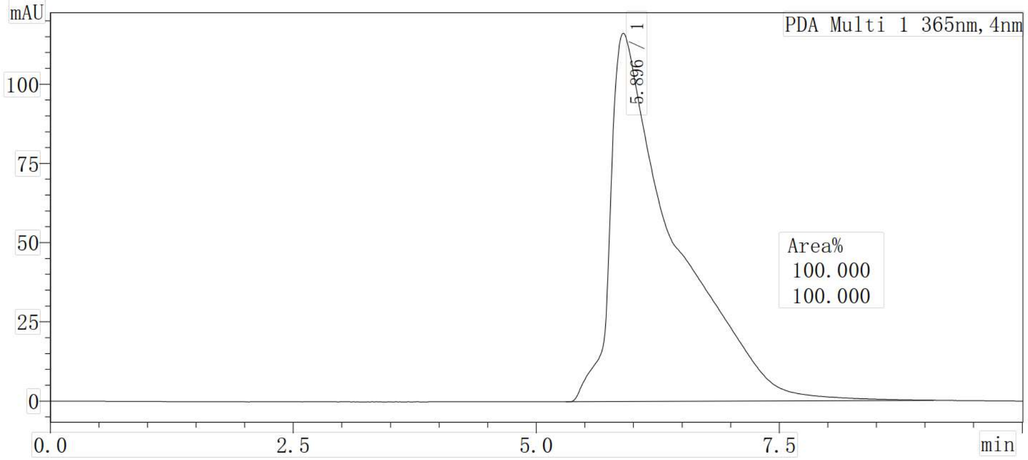

Figure S19. The NMR, HRMS and HPLC spectra of compound **6r**.

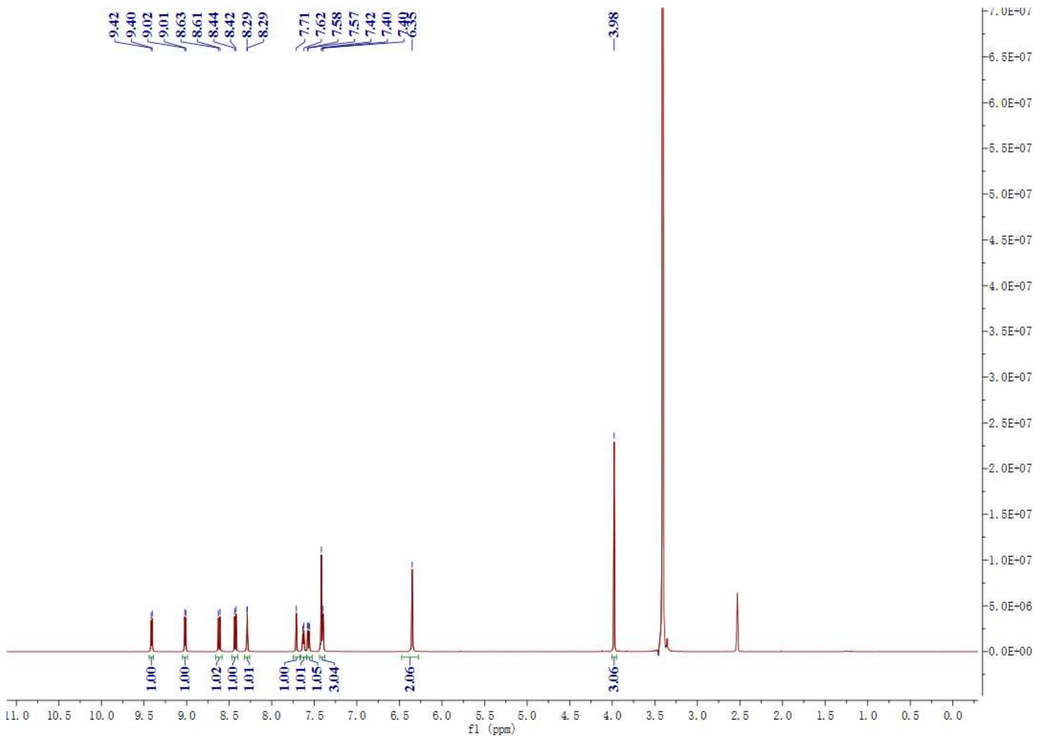

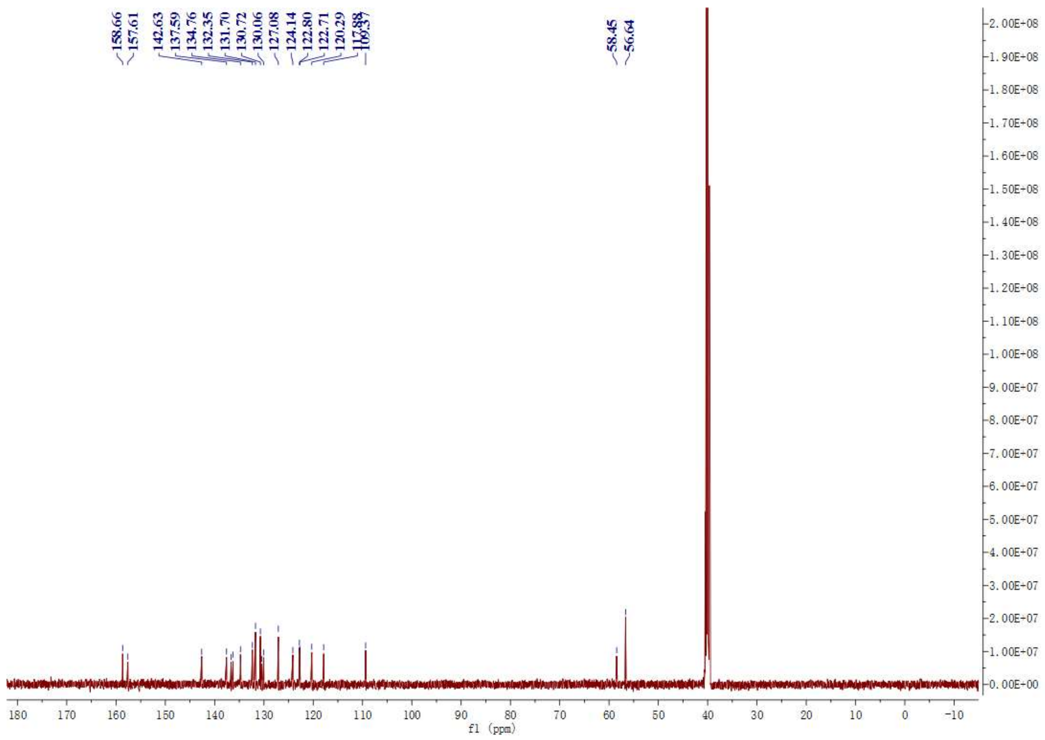

158.66
157.61
142.63
137.59
134.76
132.35
131.70
130.72
130.06
127.08
124.14
122.80
122.71
120.29
117.88
109.37
58.45
56.64
2.00E+08
1.90E+08
1.80E+08
1.70E+08
1.60E+08
1.50E+08
1.40E+08
1.30E+08
1.20E+08
1.10E+08
1.00E+08
9.00E+07
8.00E+07
7.00E+07
6.00E+07
5.00E+07
4.00E+07
3.00E+07
2.00E+07
1.00E+07
0.00E+00
180 170 160 150 140 130 120 110 100 90 80 70 60 50 40 30 20 10 0 -10
f1 (ppm)

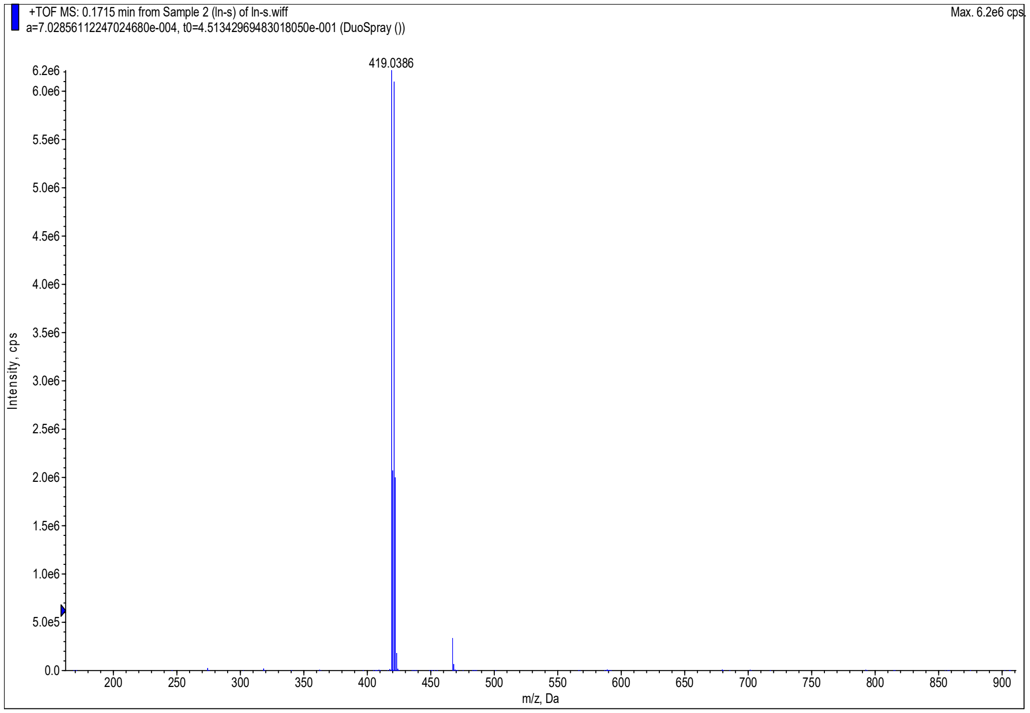

+TOF MS: 0.1715 min from Sample 2 (ln-s) of ln-s.wiff
a=7.02856112247024680e-004, t0=4.51342969483018050e-001 (DuoSpray ())
Max. 6.2e6 cps.
419.0386
Intensity, cps
m/z, Da

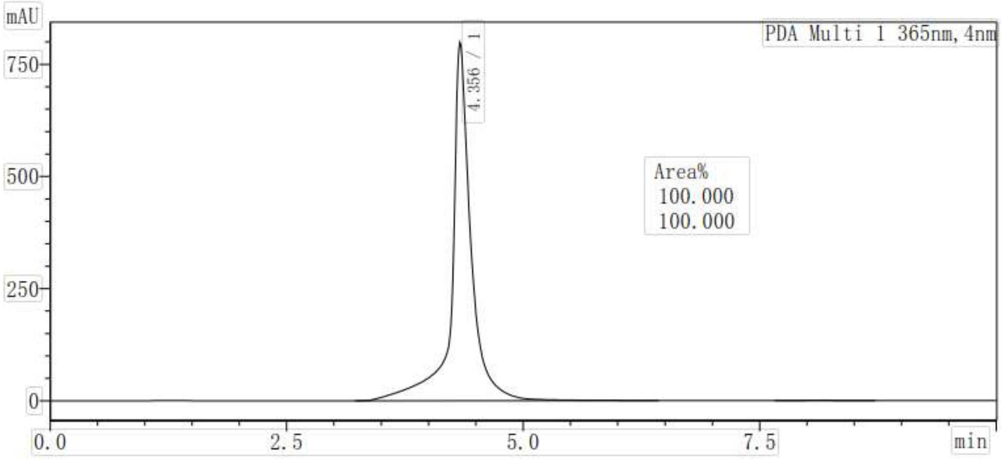

Figure S20. The NMR, HRMS and HPLC spectra of compound **6s**.

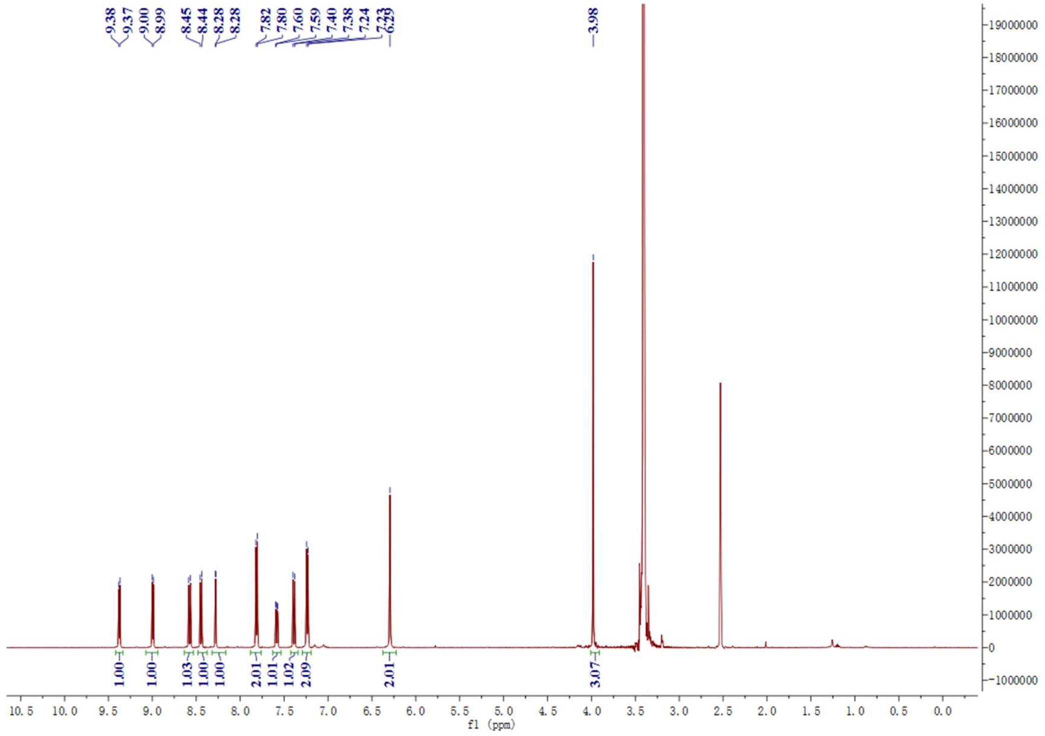

9.38
9.37
9.00
8.99
8.45
8.44
8.28
8.28
7.82
7.80
7.60
7.59
7.40
7.38
7.24
7.23
6.29
3.98
1.00
1.00
1.03
1.00
1.00
2.01
1.01
1.02
2.09
2.01
3.07
f1 (ppm)

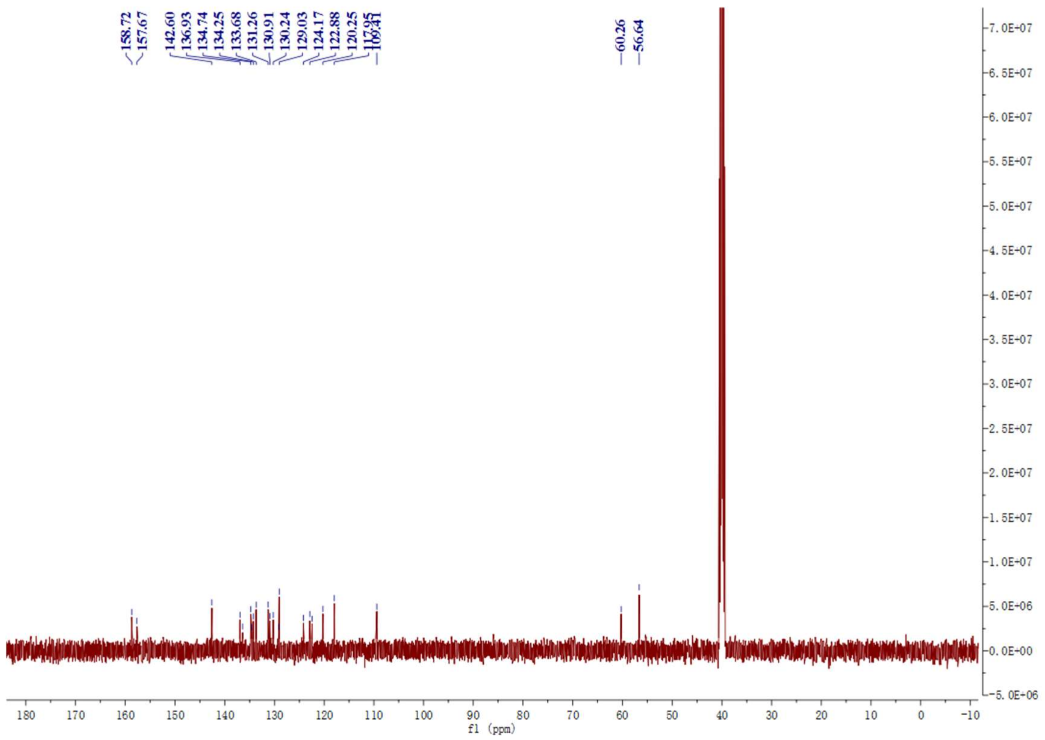

158.72
157.67
142.60
136.93
134.74
134.25
133.68
131.26
130.91
130.24
129.03
124.17
122.88
120.25
117.95
109.41
60.26
56.64
f1 (ppm)

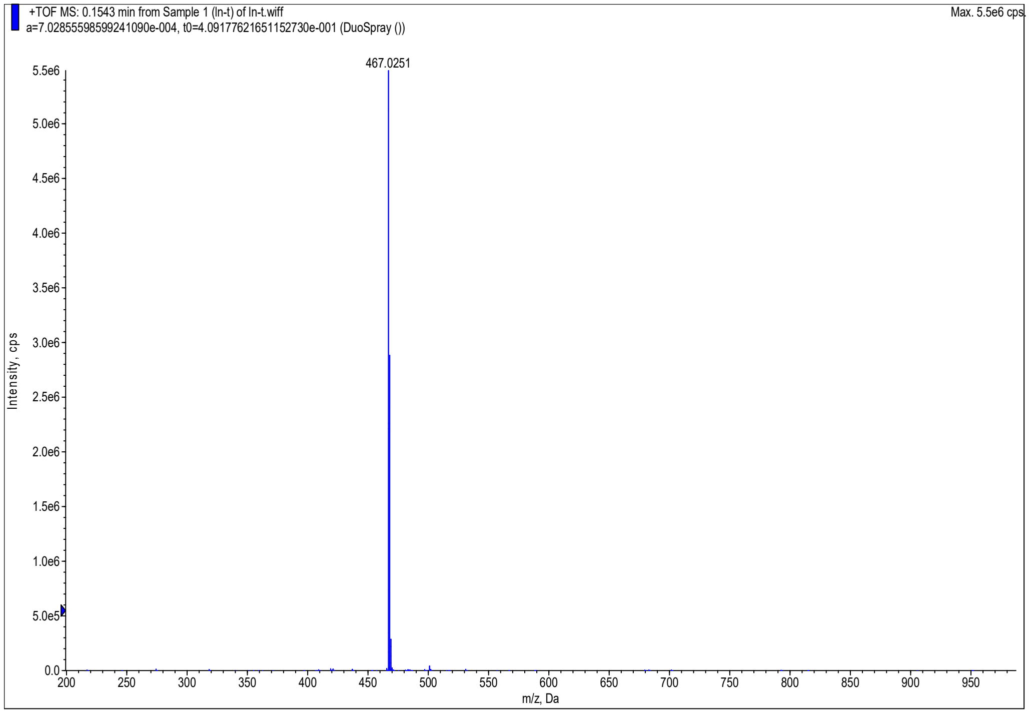

+TOF MS: 0.1543 min from Sample 1 (In-t) of In-t.wiff
a=7.02855598599241090e-004, t0=4.09177621651152730e-001 (DuoSpray ())
Max. 5.5e6 cps.
467.0251
Intensity, cps
5.5e6
5.0e6
4.5e6
4.0e6
3.5e6
3.0e6
2.5e6
2.0e6
1.5e6
1.0e6
5.0e5
0.0
200
250
300
350
400
450
500
550
600
650
700
750
800
850
900
950
m/z, Da

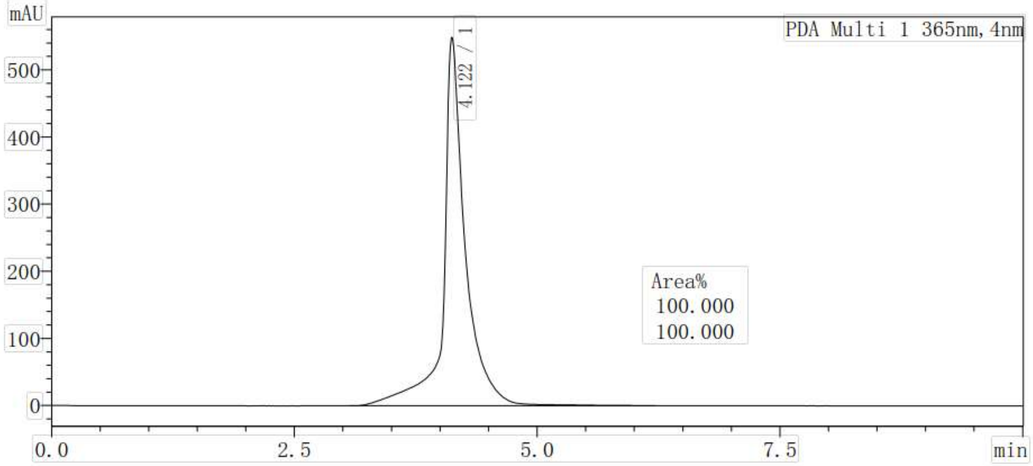

Figure S21. The NMR, HRMS and HPLC spectra of compound **6t**.

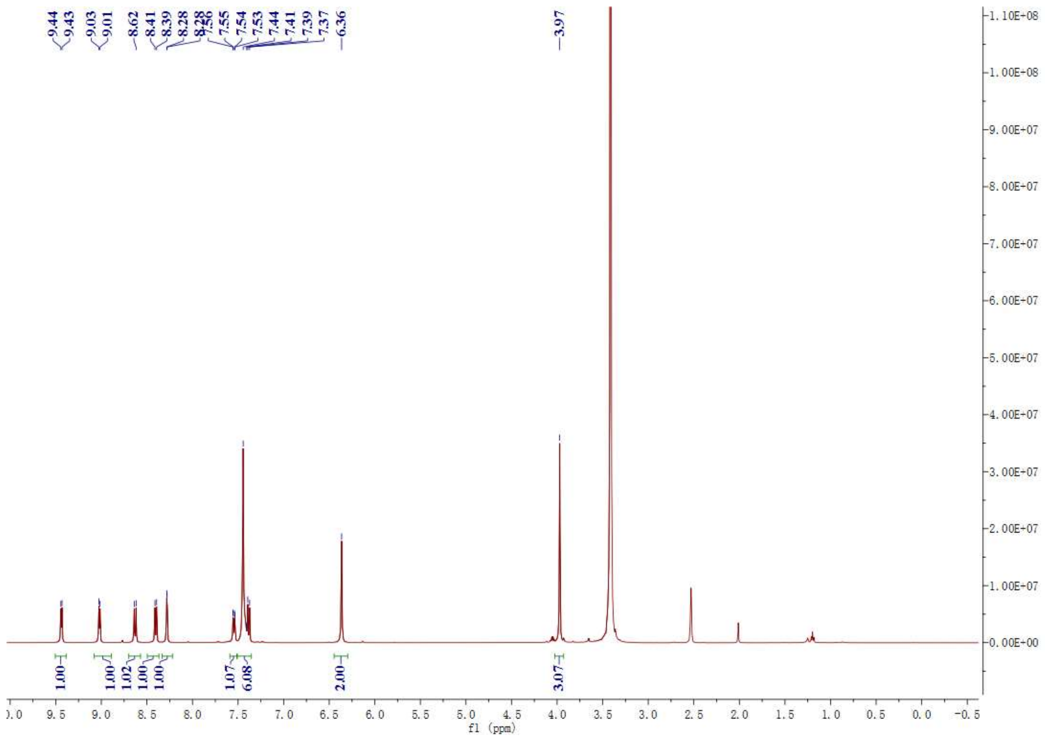
9.44
9.43
9.03
9.01
8.62
8.41
8.39
8.28
8.28
7.56
7.55
7.54
7.53
7.44
7.41
7.39
7.37
6.36
3.97
1.00
1.00
1.02
1.00
1.00
1.07
6.08
2.00
3.07
f1 (ppm)

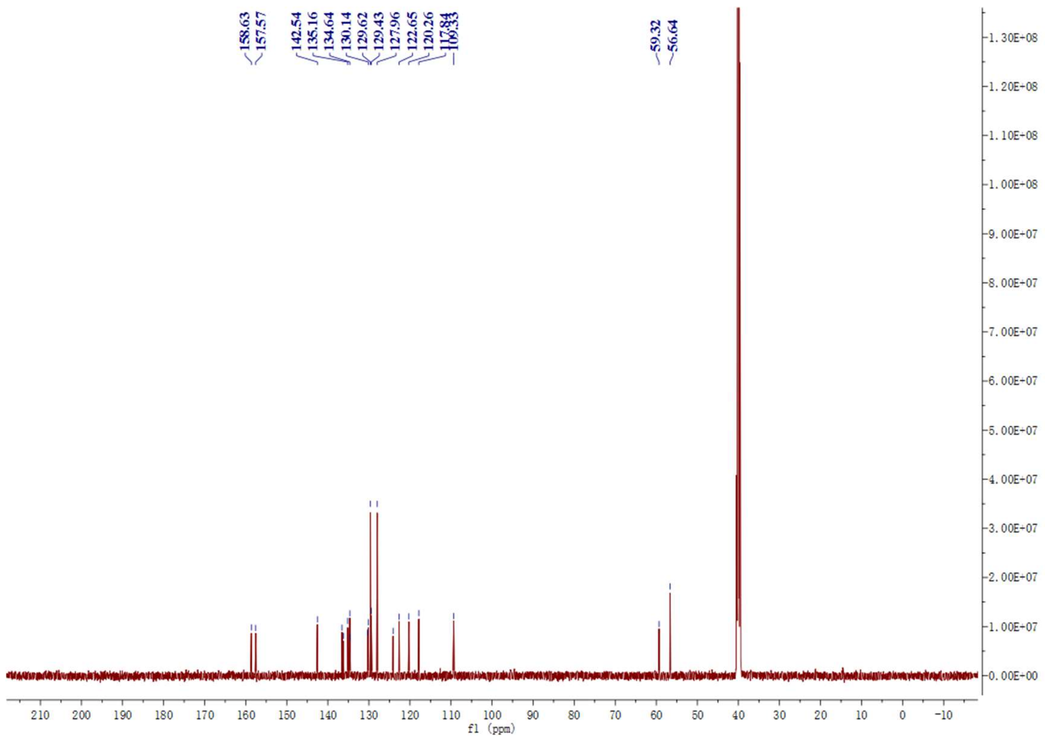
158.63
157.57
142.54
135.16
134.64
130.14
129.62
129.43
127.96
122.65
120.26
117.84
109.33
59.32
56.64
1.30E+08
1.20E+08
1.10E+08
1.00E+08
9.00E+07
8.00E+07
7.00E+07
6.00E+07
5.00E+07
4.00E+07
3.00E+07
2.00E+07
1.00E+07
0.00E+00
210 200 190 180 170 160 150 140 130 120 110 100 90 80 70 60 50 40 30 20 10 0 -10
f1 (ppm)

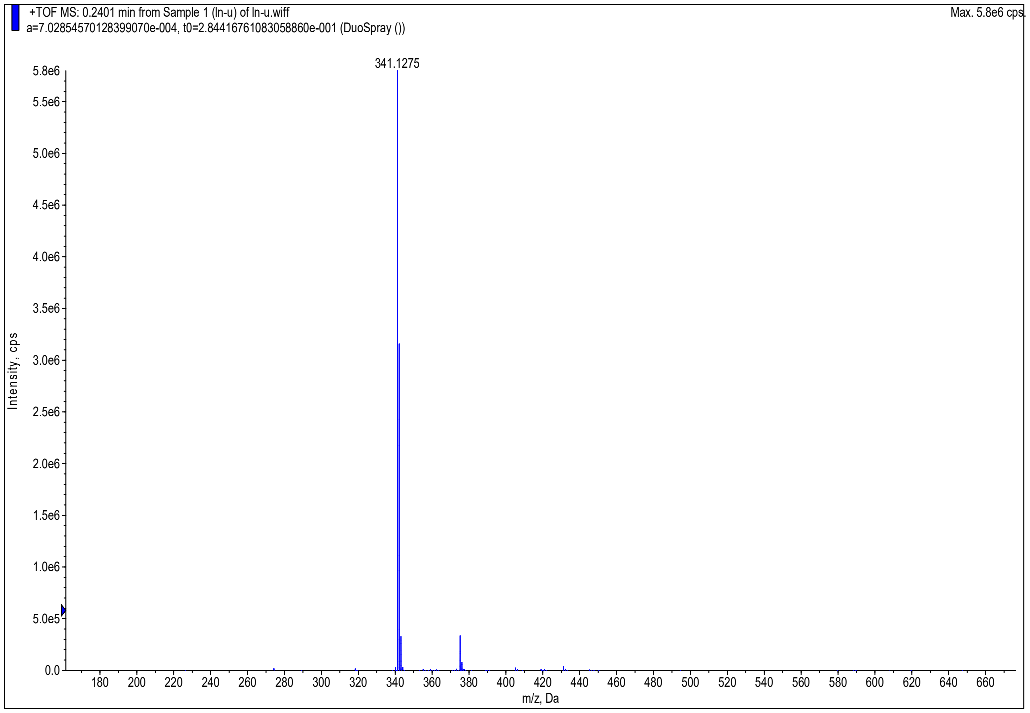
+TOF MS: 0.2401 min from Sample 1 (ln-u) of ln-u.wiff
a=7.02854570128399070e-004, t0=2.84416761083058860e-001 (DuoSpray ())
Max. 5.8e6 cps.
341.1275
Intensity, cps
5.8e6
5.5e6
5.0e6
4.5e6
4.0e6
3.5e6
3.0e6
2.5e6
2.0e6
1.5e6
1.0e6
5.0e5
0.0
180 200 220 240 260 280 300 320 340 360 380 400 420 440 460 480 500 520 540 560 580 600 620 640 660
m/z, Da

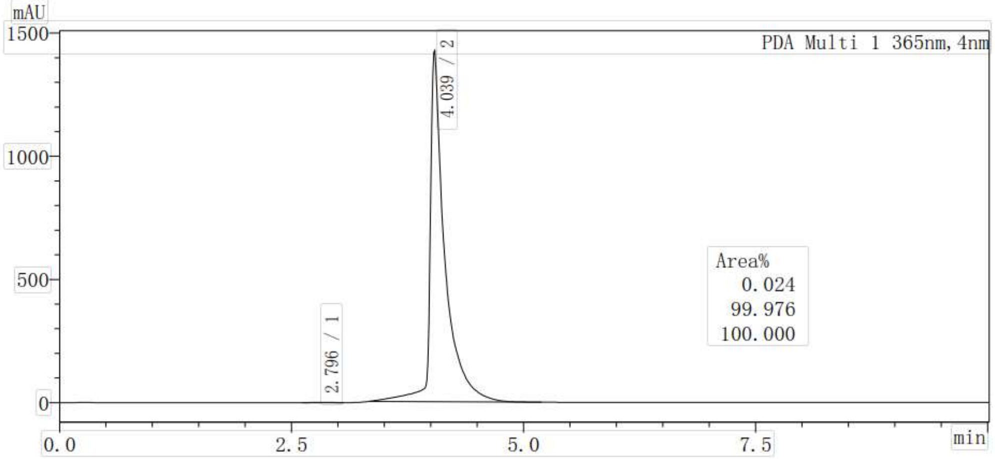

Figure S22. The NMR, HRMS and HPLC spectra of compound **6u**.

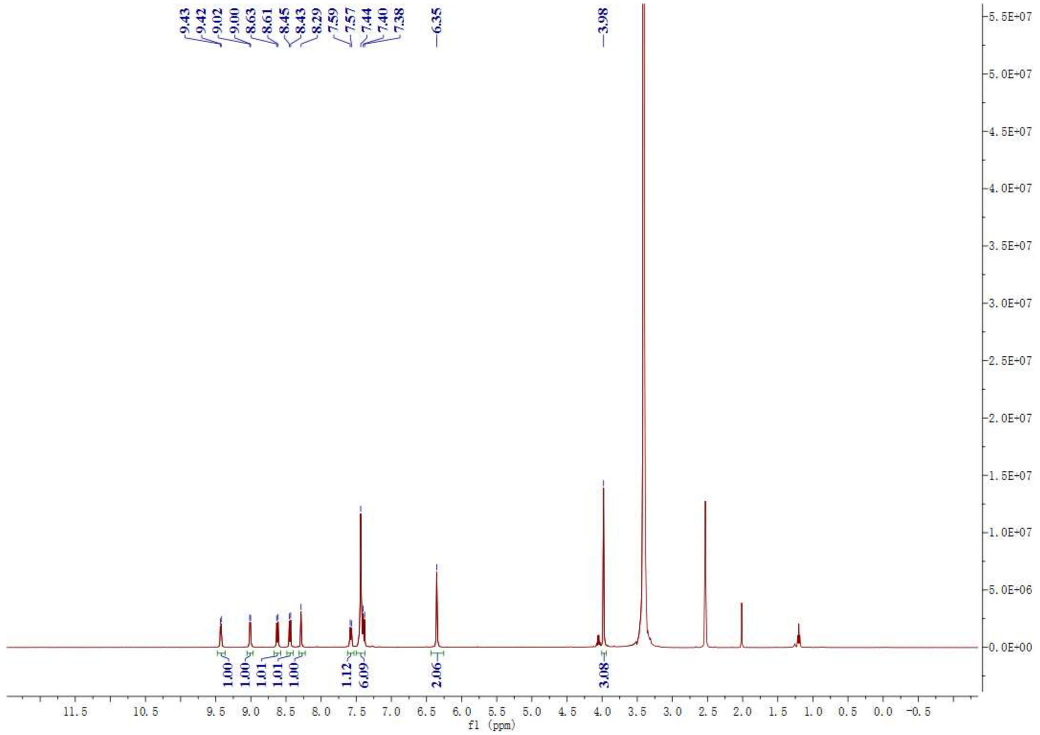

9.43
9.42
9.02
9.00
8.63
8.61
8.45
8.43
8.29
7.59
7.57
7.44
7.40
7.38
6.35
3.98
1.00
1.00
1.01
1.01
1.00
1.12
6.09
2.06
3.08
f1 (ppm)

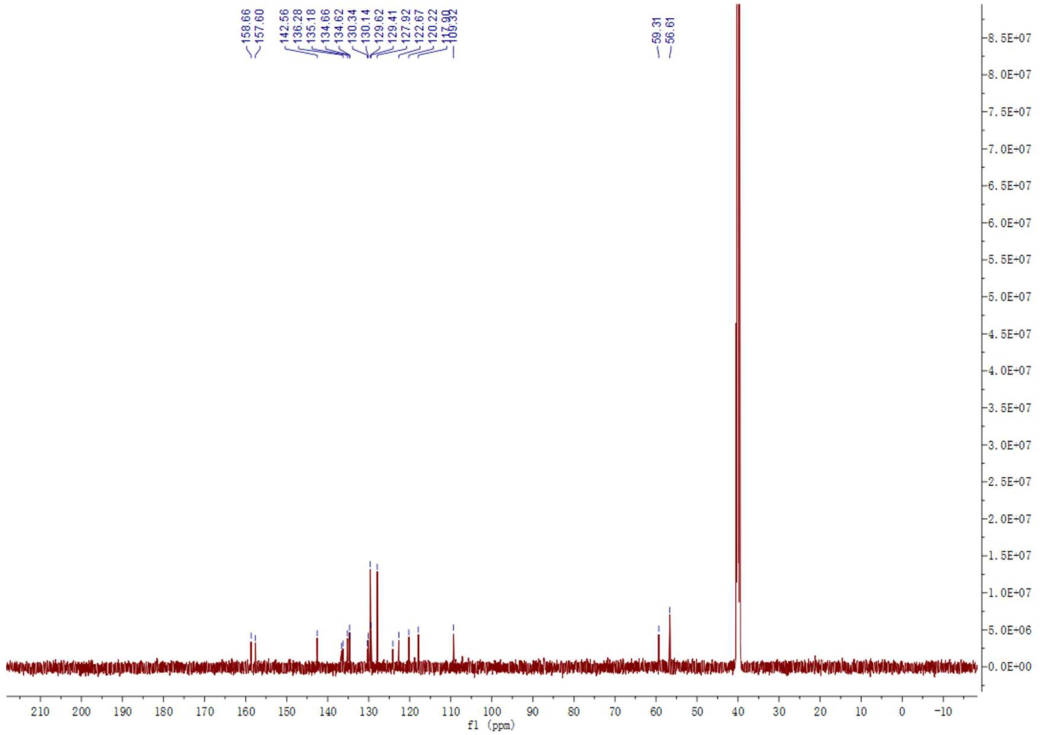
158.66
157.60
142.56
136.28
135.18
134.66
134.62
130.34
130.14
129.62
129.41
127.92
122.67
120.22
117.90
109.32
59.31
56.61
8.5E+07
8.0E+07
7.5E+07
7.0E+07
6.5E+07
6.0E+07
5.5E+07
5.0E+07
4.5E+07
4.0E+07
3.5E+07
3.0E+07
2.5E+07
2.0E+07
1.5E+07
1.0E+07
5.0E+06
0.0E+00
210 200 190 180 170 160 150 140 130 120 110 100 90 80 70 60 50 40 30 20 10 0 -10
f1 (ppm)

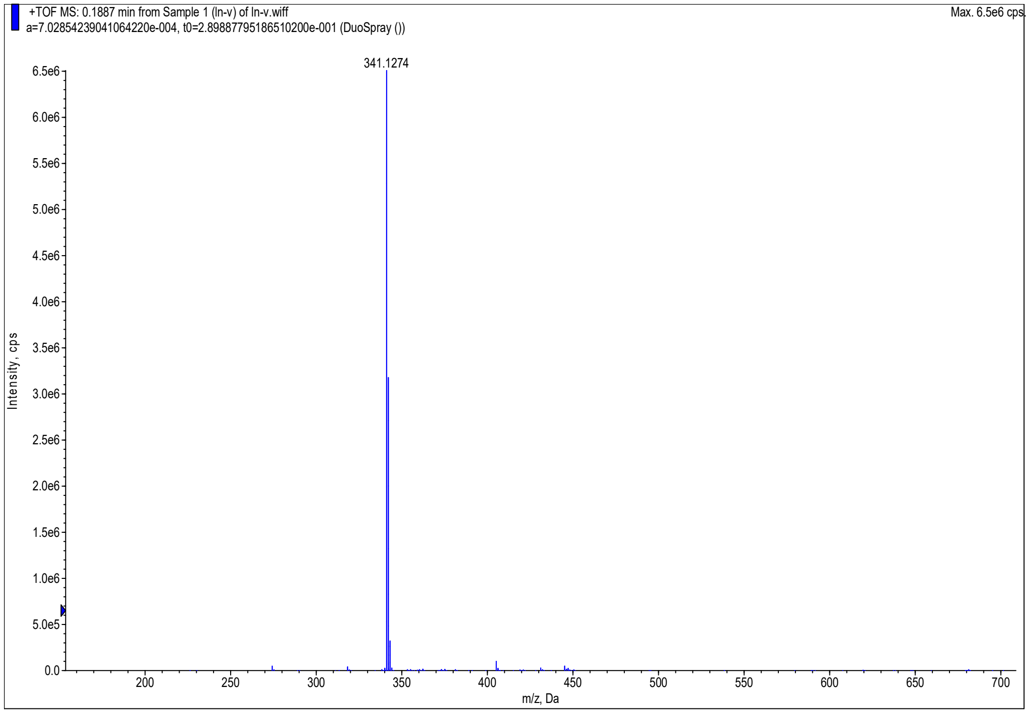
+TOF MS: 0.1887 min from Sample 1 (In-v) of In-v.wiff
a=7.02854239041064220e-004, t0=2.89887795186510200e-001 (DuoSpray ())
Max. 6.5e6 cps.
341.1274
Intensity, cps
m/z, Da

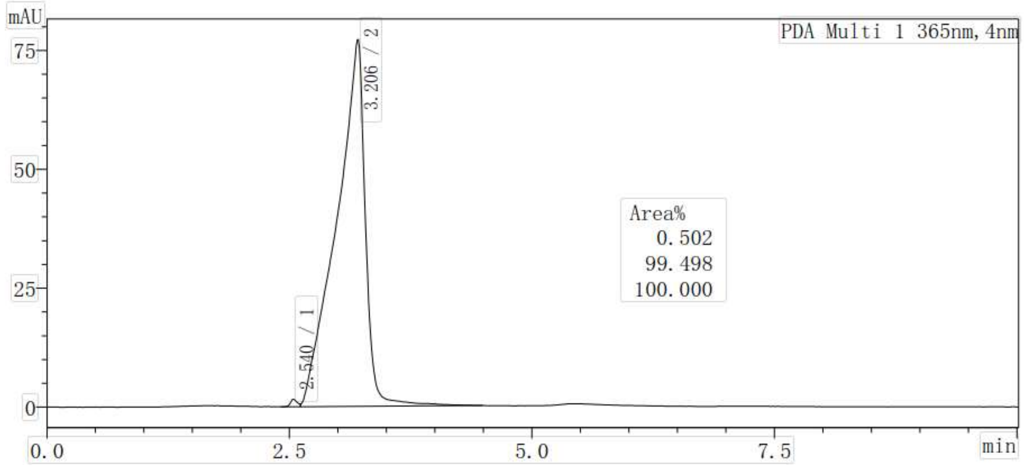

Figure S23. The NMR, HRMS and HPLC spectra of compound **6v**.
